# Learnable latent embeddings for joint behavioural and neural analysis

**DOI:** 10.1038/s41586-023-06031-6

**Published:** 2023-05-03

**Authors:** Steffen Schneider, Jin Hwa Lee, Mackenzie Weygandt Mathis

**Affiliations:** Brain Mind Institute & Neuro X Institute, École Polytechnique Fédérale de Lausanne, Geneva, Switzerland

**Keywords:** Neural decoding, Machine learning

## Abstract

Mapping behavioural actions to neural activity is a fundamental goal of neuroscience. As our ability to record large neural and behavioural data increases, there is growing interest in modelling neural dynamics during adaptive behaviours to probe neural representations^1–3^. In particular, although neural latent embeddings can reveal underlying correlates of behaviour, we lack nonlinear techniques that can explicitly and flexibly leverage joint behaviour and neural data to uncover neural dynamics^3–5^. Here, we fill this gap with a new encoding method, CEBRA, that jointly uses behavioural and neural data in a (supervised) hypothesis- or (self-supervised) discovery-driven manner to produce both consistent and high-performance latent spaces. We show that consistency can be used as a metric for uncovering meaningful differences, and the inferred latents can be used for decoding. We validate its accuracy and demonstrate our tool’s utility for both calcium and electrophysiology datasets, across sensory and motor tasks and in simple or complex behaviours across species. It allows leverage of single- and multi-session datasets for hypothesis testing or can be used label free. Lastly, we show that CEBRA can be used for the mapping of space, uncovering complex kinematic features, for the production of consistent latent spaces across two-photon and Neuropixels data, and can provide rapid, high-accuracy decoding of natural videos from visual cortex.

## Abstract (web summary)

A new encoding method, CEBRA, jointly uses behavioural and neural data in a (supervised) hypothesis- or (self-supervised) discovery-driven manner to produce both consistent and high-performance latent spaces.

## Main

A central quest in neuroscience is the neural origin of behaviour^1,2^. Nevertheless, we are still limited in both the number of neurons and length of time we can record from behaving animals in a session. Therefore, we need new methods that can combine data across animals and sessions with minimal assumptions, thereby generating interpretable neural embedding spaces^1,3^. Current tools for representation learning are either linear or, if nonlinear, typically rely on generative models and they do not yield consistent embeddings across animals (or repeated runs of the algorithm). Here, we combine recent advances in nonlinear disentangled representation learning and self-supervised learning to develop a new dimensionality reduction method that can be applied jointly to behavioural and neural recordings to show meaningful lower-dimensional neural population dynamics^3–5^.

From data visualization (clustering) to discovery of latent spaces that explain neural variance, dimensionality reduction of behaviour or neural data has been impactful in neuroscience. For example, complex three-dimensional (3D) forelimb reaching can be reduced to between only eight and twelve dimensions^6,7^, and low-dimensional embeddings show some robust aspects of movements (for example, principal component analysis (PCA)-based manifolds in which the neural state space can easily be constrained and is stable across time^8–10^). Linear methods such as PCA are often used to increase interpretability, but this comes at the cost of performance^1^. Uniform manifold approximation and projection (UMAP)^11^ and *t*-distributed stochastic neighbour embedding (*t*-SNE)^12^ are excellent nonlinear methods but they lack the ability to explicitly use time information, which is always available in neural recordings, and they are not as directly interpretable as PCA. Nonlinear methods are desirable for use in high-performance decoding but often lack identifiability—the desirable property that true model parameters can be determined, up to a known indeterminacy^13,14^. This is critical because it ensures that the learned representations are uniquely determined and thus facilitates consistency across animals and/or sessions.

There is recent evidence that label-guided variational auto-encoders (VAEs) could improve interpretability^5,15,16^. Namely, by using behavioural variables, such algorithms can learn to project future behaviour onto past neural activity^15^, or explicitly to use label priors to shape the embedding^5^. However, these methods still have restrictive explicit assumptions on the underlying statistics of the data and they do not guarantee consistent neural embeddings across animals^5,17,18^, which limits both their generalizability and interpretability (and thereby affects accurate decoding across animals).

We address these open challenges with CEBRA, a new self-supervised learning algorithm for obtaining interpretable, consistent embeddings of high-dimensional recordings using auxiliary variables. Our method combines ideas from nonlinear independent component analysis (ICA) with contrastive learning^14,19–21^, a powerful self-supervised learning scheme, to generate latent embeddings conditioned on behaviour (auxiliary variables) and/or time. CEBRA uses a new data-sampling scheme to train a neural network encoder with a contrastive optimization objective to shape the embedding space. It can also generate embeddings across multiple subjects and cope with distribution shifts among experimental sessions, subjects and recording modalities. Importantly, our method relies on neither data augmentation (as does SimCLR^22^) nor a specific generative model, which would limit its range of use.

## Joint behavioural and neural embeddings

**Fig. 1:**
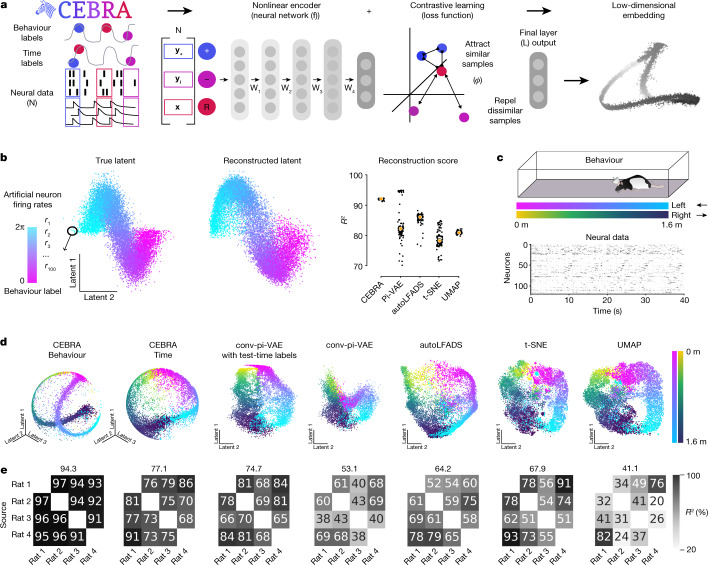
Use of CEBRA for consistent and interpretable embeddings. **a**, CEBRA allows for self-supervised, supervised and hybrid approaches for both hypothesis-and discovery-driven analysis. Overview of pipeline: collect data (for example, pairs of behaviour (or time) and neural data (**x**,**y**)), determine positive and negative pairs, train CEBRA and produce embeddings. W_1,...4_ represent the neural network weights. **b**, Left, true 2D latent, where each point is mapped to the spiking rate of 100 neurons. Middle, CEBRA embedding after linear regression to the true latent. Right, reconstruction score is *R*^2^ of linear regression between the true latent and resulting embedding from each method. The ‘behaviour label’ is a 1D random variable sampled from uniform distribution of [0, 2π] that is assigned to each time bin of synthetic neural data, as visualized by the colour map. The orange line represents the median and each black dot an individual run (*n* = 100). CEBRA-Behaviour shows a significantly higher reconstruction score compared with pi-VAE, *t*-SNE and UMAP (one-way ANOVA, *F*(4, 495) = 251, *P* = 1.12 × 10^−117^ with post hoc Tukey’s honest significant difference *P* < 0.001). **c**, Rat hippocampus data derived from ref. ^26^. Cartoon from scidraw.io. Electrophysiology data were collected while a rat traversed a 1.6 m linear track ‘leftwards’ or ‘rightwards’. **d**, We benchmarked CEBRA against conv-pi-VAE (both with labels and without), autoLFADS, *t*-SNE and unsupervised UMAP. Note: for performance against the original pi-VAE see Extended Data Fig. 1. We plot the three latents (all CEBRA-embedding figures show the first three latents). The dimensionality of the latent space is set to the minimum and equivalent dimension per method (3D for CEBRA and 2D for others) for fair comparison. Note: higher dimensions for CEBRA can yield higher consistency values (Extended Data Fig. 7). **e**, Correlation matrices show *R*^2^ values after fitting a linear model between behaviour-aligned embeddings of pairs of rats, one as the target and the other as the source (mean, *n* = 10 runs). Parameters were picked by optimization of average run consistency across rats.

We propose a framework for jointly trained latent embeddings. CEBRA leverages user-defined labels (supervised, hypothesis-driven) or time-only labels (self-supervised, discovery-driven; Fig. 1a and Supplementary Note 1) to obtain consistent embeddings of neural activity that can be used for both visualization of data and downstream tasks such as decoding. Specifically, it is an instantiation of nonlinear ICA based on contrastive learning^14^. Contrastive learning is a technique that leverages contrasting samples (positive and negative) against each other to find attributes in common and those that separate them. We can use discrete and continuous variables and/or time to shape the distribution of positive and negative pairs, and then use a nonlinear encoder (here, a convolutional neural network but can be another type of model) trained with a new contrastive learning objective. The encoder features form a low-dimensional embedding of the data (Fig. 1a). Generation of consistent embeddings is highly desirable and closely linked to identifiability in nonlinear ICA^14,23^. Theoretical work has shown that the use of contrastive learning with auxiliary variables is identifiable for bijective neural networks using a noise contrastive estimation (NCE) loss^14^, and that with an InfoNCE loss this bijectivity assumption can sometimes be removed^24^ (see also our theoretical generalization in Supplementary Note 2). InfoNCE minimization can be viewed as a classification problem such that, given a reference sample, the correct positive sample needs to be distinguished from multiple negative samples.

CEBRA optimizes neural networks **f**, **f**′ that map neural activity to an embedding space of a defined dimension (Fig. 1a). Pairs of data (**x**, **y**) are mapped to this embedding space and then compared with a similarity measure *ϕ*(⋅,⋅). Abbreviating this process with $\psi \left({\bf{x}},{\bf{y}}\right)=\varphi \left({\bf{f}}\left({\bf{x}}\,\right),{{\bf{f}}}^{{\prime} }\left({\bf{y}}\right)\right)/\tau$ and a temperature hyperparameter, *τ*, the full criterion for optimization is

$$\mathop{{\mathbb{E}}}\limits_{\begin{array}{c}{\bf{x}}\sim p({\bf{x}}),\,{{\bf{y}}}_{+}\sim p({\bf{y}}|{\bf{x}})\\ {{\bf{y}}}_{1},\ldots ,{{\bf{y}}}_{n}\sim q({\bf{y}}|{\bf{x}})\end{array}}\,[\,-\psi ({\bf{x}},{{\bf{y}}}_{+})+\log \mathop{\sum }\limits_{i=1}^{n}{e}^{\psi ({\bf{x}},{{\bf{y}}}_{i})}],$$

which, depending on the dataset size, can be optimized with algorithms for either batch or stochastic gradient descent.

In contrast to other contrastive learning algorithms, the positive-pair distribution *p* and negative-pair distribution *q* can be systematically designed and allow the use of time, behaviour and other auxiliary information to shape the geometry of the embedding space. If only discrete labels are used, this training scheme is conceptually similar to supervised contrastive learning^21^.

CEBRA can leverage continuous behavioural (kinematics, actions) as well as other discrete variables (trial ID, rewards, brain-area ID and so on). Additionally, user-defined information about desired invariances in the embedding is used (across animals, sessions and so on), allowing for flexibility in data analysis. We group this information into task-irrelevant and -relevant variables, and these can be leveraged in different contexts. For example, to investigate trial-to-trial variability or learning across trials, information such as a trial ID would be considered a task-relevant variable. On the contrary, if we aim to build a robust brain machine interface that should be invariant to such short-term changes, we would include trial information as a task-irrelevant variable and obtain an embedding space that no longer carries this information. Crucially, this allows inference of latent embeddings without explicit modelling of the data-generating process (as done in pi-VAE^5^ and latent factor analysis via dynamical systems (LFADS)^17^). Omitting the generative model and replacing it by a contrastive learning algorithm facilitates broader applicability without modifications.

## Robust and decodable latent embeddings

**Extended Data Fig. 1:**
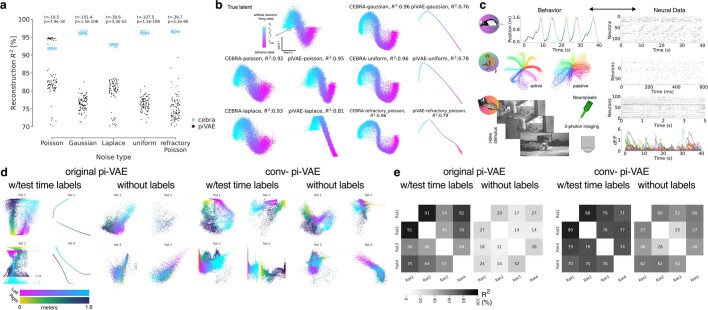
Overview of datasets, synthetic data, & original pi-VAE implementation vs. modified conv-pi-VAE. **a**, We generated synthetic datasets similar to Fig. 1b with additional variations in the noise distributions in the generative process. We benchmarked the reconstruction score of the true latent using CEBRA and pi-VAE (n = 100 seeds) on the generated synthetic datasets. CEBRA showed higher and less variable reconstruction scores than pi-VAE in all noise types (one-sided Welch’s t-test, corrected using the Holm-Bonferroni method, t and p-values indicated on the plot). **b**, Example visualization of the reconstructed latents from CEBRA and pi-VAE on different synthetic dataset types. **c**, We benchmarked and demonstrate the abilities of CEBRA on four datasets. Rat-based electrophysiology data^26^, where the animal transverse a 1.6 meter linear track “leftwards” or “rightwards”. Two mouse-based datasets: one 2-photon calcium imaging passively viewing dataset^46^, and one with the same stimulus but recorded with Neuropixels^47^. A monkey-based electrophysiology dataset of centre out reaching from Chowdhury et al.^34^, and processed to trial data as in ref. ^52^. **d**, Conv-pi-VAE showed improved performance, both with labels (Wilcoxon signed-rank test, *P* = 0.0341) and without labels Wilcoxon signed-rank test, *P*= 0.0005). Example runs/embeddings the consistency across rats, with **e**, consistency across rats, from target to source, as computed in Fig. 1. Cartoon animals are adapted from scidraw.io.

We first demonstrate that CEBRA significantly outperforms *t*-SNE, UMAP, automatic LFADS (autoLFADS)^25^ and pi-VAE (the latter was shown to outperform PCA, LFADS, demixed PCA and PfLDS (Poisson feed-forward neural network linear dynamical system) on some tasks) in the reconstruction of ground truth synthetic data (one-way analysis of variance (ANOVA), *F*(4, 495) = 251, *P* = 1.12 × 10^−117^; Fig. 1b and Extended Data Fig. 1a,b).

**Extended Data Fig. 2:**
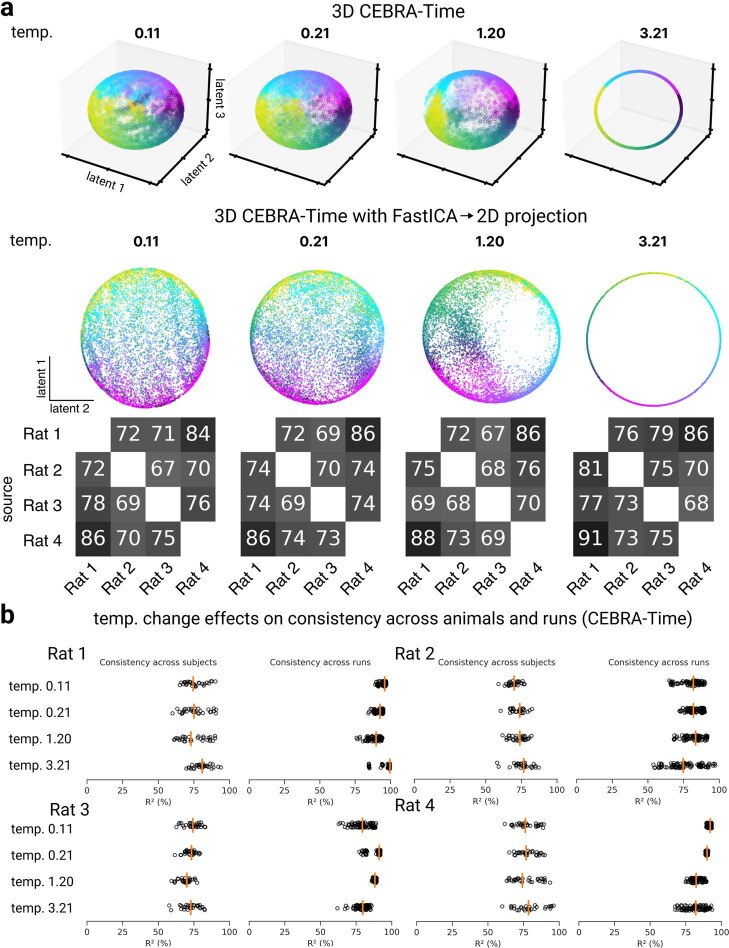
Hyperparameter changes on visualization and consistency. **a**, Temperature has the largest effect on visualization (vs. consistency) of the embedding as shown by a range from 0.1 to 3.21 (highest consistency for rat 1), as can be appreciated in 3D (top) and post FastICA into a 2D embedding (middle). Bottom row shows the corresponding change on mean consistency, and in **b**, the variance can be noted. Orange line denotes the median and black dots are individual runs (subject consistency: 10 runs with 3 comparisons per rat; run consistency: 10 runs, each compared to 9 remaining runs).

**Extended Data Fig. 3:**
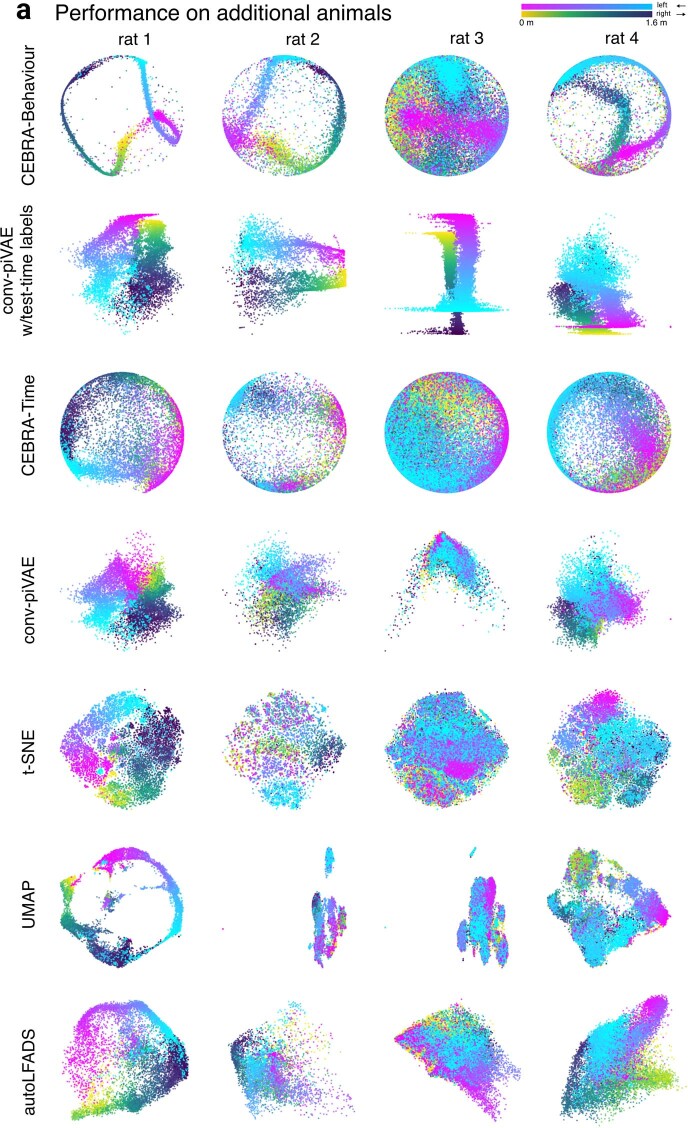
CEBRA produced consistent, highly decodable embeddings. **a**, Additional rat data shown for all algorithms we benchmarked (see Methods). For CEBRA-Behaviour, we used temperature 1, time offset 10, batch size 512 and 10k training steps. For CEBRA-Time, we used temperature 2.25, time offset 10, batch size 512 and 4k training steps. For UMAP, we used the cosine metric and *min*_*dist* of 0.99 and *n*_*neighbors* of 31. For t-SNE we used cosine metric and *perplexity* of 29. For conv-pi-VAE, we trained 1000 epochs with learning rate 2.5 x 10^*−*4^. For autoLFADS we used the in-built ray-tune framework for finding optimal hyperparameters. CEBRA was trained with output latent 3D (the minimum) and all other methods were trained with a 2D latent.

We then turned to a hippocampus dataset that was used to benchmark neural embedding algorithms^5,26^ (Extended Data Fig. 1c and Supplementary Note 1). Of note, we first significantly improved pi-VAE by the addition of a convolutional neural network (conv-pi-VAE), thereby allowing this model to leverage multiple time steps, and used this for further benchmarking (Extended Data Fig. 1d,e). To test our methods, we first considered the correlation of the resulting embedding space across subjects (does it produce similar latent spaces?), and the correlation across repeated runs of the algorithm (how consistent are the results?). We found that CEBRA significantly outperformed other algorithms in the production of consistent embeddings, and it produced visually informative embeddings (Fig. 1c–e and Extended Data Figs. 2 and 3; for each embedding a single point represents the neural population activity over a specified time bin).

**Extended Data Fig. 4:**
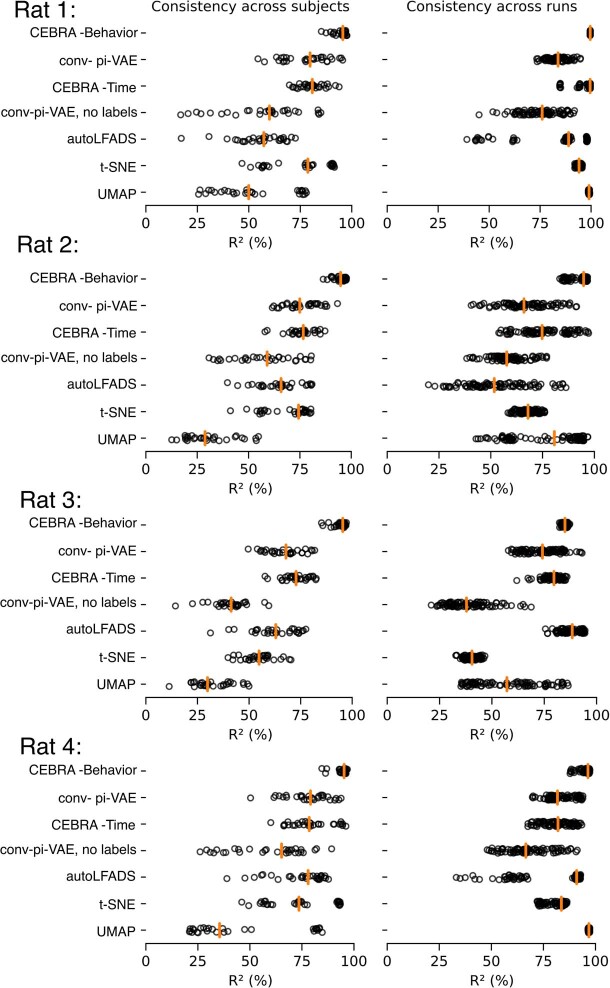
Additional metrics used for benchmarking consistency. Comparisons of all algorithms along different metrics for rats 1, 2, 3, 4. The orange line is median across n = 10 runs, black circles denote individual runs. Each run is the average over three non-overlapping test splits.

When using CEBRA-Behaviour, the consistency of the resulting embedding space across subjects is significantly higher compared with autoLFADS and conv-pi-VAE, with or without test-time labels (one-way ANOVA *F*(25.4) *P* = 1.92 × 10^−16^; Supplementary Table 1 and Fig. 1d,e). Qualitatively, it can be appreciated that both CEBRA-Behaviour and -Time have similar output embeddings whereas the latents from conv-pi-VAE, either with label priors or without labels, are not consistent (CEBRA does not need test-time labels), suggesting that the label prior strongly shapes the output embedding structure of conv-pi-VAE. We also considered correlations across repeated runs of the algorithm, and found higher consistency and lower variability with CEBRA (Extended Data Fig. 4).

## Hypothesis-driven and discovery-driven analyses

**Fig. 2:**
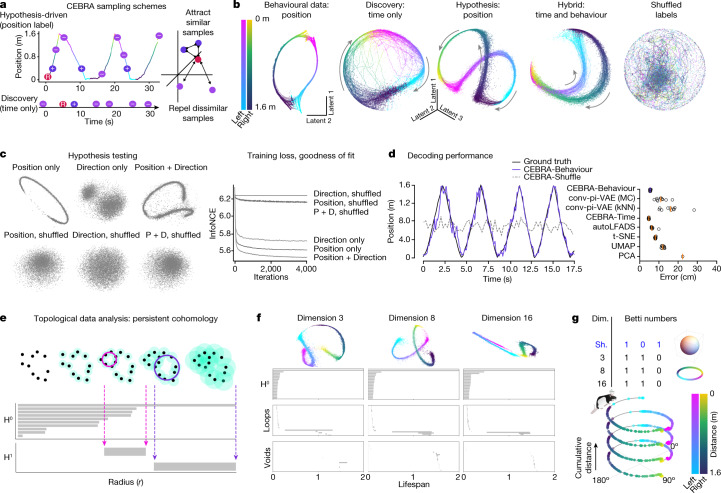
Hypothesis- and discovery-driven analysis with CEBRA. **a**, CEBRA can be used in any of three modes: hypothesis-driven mode, discovery-driven mode, or hybrid mode, which allows for weaker priors on the latent embedding. **b**, Left to right, CEBRA on behavioural data using position as a label, CEBRA-Time, CEBRA-Behaviour (on neural data) with position hypothesis, CEBRA-Hybrid (a five-dimensional space was used, in which 3D is first guided by both behaviour + time and the final 2D is guided by time) and shuffled (erroneous). **c**, Embeddings with position (P) only, direction (D) only and P + D only, and shuffled position only, direction only and P + D only, for hypothesis testing. The loss function can be used as a metric for embedding quality. **d**, Left, we utilized either hypothesis-driven P + D or shuffle (erroneous) to decode the position of the rat, which yielded a large difference in decoding performance: P + D *R*^2^ = 73.35 versus −49.90% for shuffled, and median absolute error 5.8 versus 44.7 cm. Purple line represents decoding from the 3D hypothesis-based latent space; dashed line is shuffled. Right, performance across additional methods (orange bars indicate the median of individual runs (*n* = 10), indicated by black circles. Each run is averaged over three splits of the dataset). MC, Monte Carlo. **e**, Schematic showing how persistent cohomology is computed. Each data point is thickened to a ball of gradually expanding radius (*r*) while tracking the birth and death of ‘cycles’ in each dimension. Prominent lifespans, indicated by pink and purple arrows, are considered to determine Betti numbers. **f**, Top, visualization of neural embeddings computed with different input dimensions. Bottom, related persistent cohomology lifespan diagrams. **g**, Betti numbers from shuffled embeddings (sh.) and across increasing dimensions (dim.) of CEBRA, and the topology-preserving circular coordinates using the first cocycle from persistent cohomology analysis (Methods).

Among the advantages of CEBRA are its collective flexibility, limited assumptions, and ability to test hypotheses. For the hippocampus, one can hypothesize that these neurons represent space^27,28^ and therefore the behavioural label could be either position or velocity (Fig. 2a). In addition, considering structure in only the behavioural data (with CEBRA) could help refine which behavioural labels to use jointly with neural data (Fig. 2b). Conversely, for the sake of argument, we could have an alternative hypothesis: that the hippocampus does not map space, but simply maps the direction of travel or some other feature. Using the same model but hypothesis free, and using time for selection of contrastive pairs, is also possible, and/or a hybrid thereof (Fig. 2a,b).

We trained hypothesis-guided (supervised), time-only (self-supervised) and hybrid models across a range of input dimensions and embedded the neural latents into a 3D space for visualization. Qualitatively, we find that the position-based model produces a highly smooth embedding that shows the position of the animal—namely, there is a continuous ‘loop’ of latent dynamics around the track (Fig. 2b). This is consistent with what is known about the hippocampus^26^ and shows the topology of the linear track with direction specificity whereas shuffling the labels, which breaks the correlation between neural activity and direction and position, produces an unstructured embedding (Fig. 2b).

CEBRA-Time produces an embedding that more closely resembles that of position (Fig. 2b). This also suggests that time contrastive learning captured the major latent space structure, independent of any label input, reinforcing the idea that CEBRA can serve both discovery- and hypothesis-driven questions (and that running both variants can be informative). The hybrid design, whose goal is to disentangle the latent to subspaces that are relevant to the given behavioural and residual temporal variance and noise, showed a structured embedding space similar to behaviour (Fig. 2b).

**Extended Data Fig. 5:**
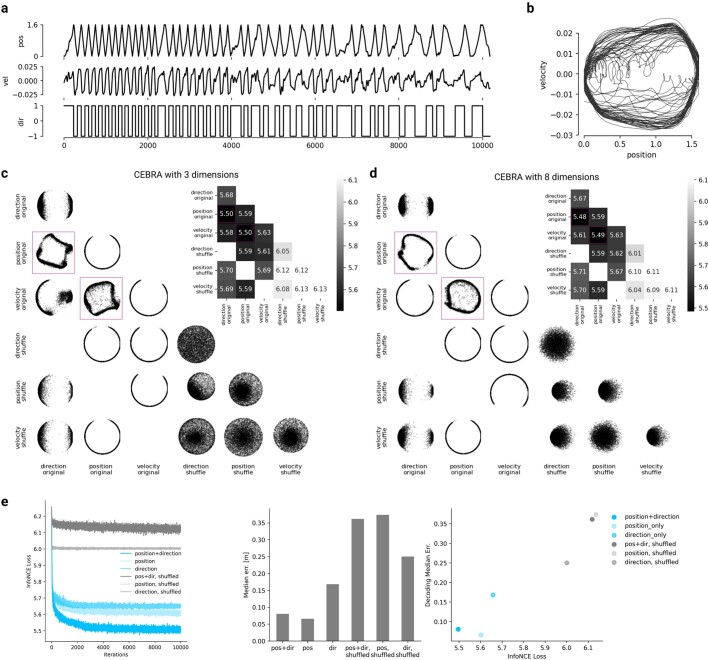
Hypothesis testing with CEBRA. **a**, Example data from a hippocampus recording session (rat 1). We tested possible relationships between three experimental variables (rat location, velocity, movement direction) and the neural recordings (120 neurons, not shown). **b**, Relationship between velocity and position. **c**, We trained CEBRA with three-dimensional outputs on every single experimental variable (main diagonal) and every combination of two variables. All variables are treated as ‘continuous’ in this experiment. We compared original to shuffled variables (shuffling is done by permuting all samples over the time dimension) as a control. We projected the original three-dimensional space onto the first principal components. We show the minimum value of the InfoNCE loss on the trained embedding for all combinations in the confusion matrix (lower number is better). Either velocity or direction, paired with position information is needed for maximum structure in the embedding (highlighted, coloured), yielding lowest InfoNCE error. **d**, Using an eight-dimensional CEBRA embedding did not qualitatively alter the results. We again report the first two principal components as well as InfoNCE training error upon convergence, and find non-trivial embeddings with lowest training error for combinations of direction/velocity and position. **e**, The InfoNCE metric can serve as the goodness of fit metric, both for hypothesis testing and identifying decodable embeddings. We trained CEBRA in discovery-driven mode with 32 latent dimensions. We compared the InfoNCE loss (left, middle) between various hypotheses. Low InfoNCE was correlated with low decoding error (right).

To quantify how CEBRA can disentangle which variable had the largest influence on embedding, we tested for encoding position, direction and combinations thereof (Fig. 2c). We find that position plus direction is the most informative label^29^ (Fig. 2c and Extended Data Fig. 5a–d). This is evident both in the embedding and the value of the loss function on convergence, which serves as a ‘goodness of fit’ metric to select the best labels—that is, which label(s) produce the lowest loss at the same point in training (Extended Data Fig. 5e). Note that erroneous (shuffled) labels converge to considerably higher loss values.

To measure performance, we consider how well we could decode behaviour from the embeddings. As an additional baseline we performed linear dimensionality reduction with PCA. We used a *k*-nearest-neighbour (kNN) decoder for position and direction and measured the reconstruction error. We find that CEBRA-Behaviour has significantly better decoding performance (Fig. 2d and Supplementary Video 1) compared with both pi-VAE and our conv-pi-VAE (one-way ANOVA, *F* = 131, *P* = 3.6 × 10^−24^), and also CEBRA-Time compared with unsupervised methods (autoLFADS, *t*-SNE, UMAP and PCA; one-way ANOVA, *F* = 1,983, *P* = 6 × 10^−50^; Supplementary Table 2). Zhou and Wei^5^ reported a median absolute decoding error of 12 cm error whereas we achieved approximately 5 cm (Fig. 2d). CEBRA therefore allows for high-performance decoding and also ensures consistent embeddings.

## Cohomology as a metric for robustness

**Extended Data Fig. 6:**
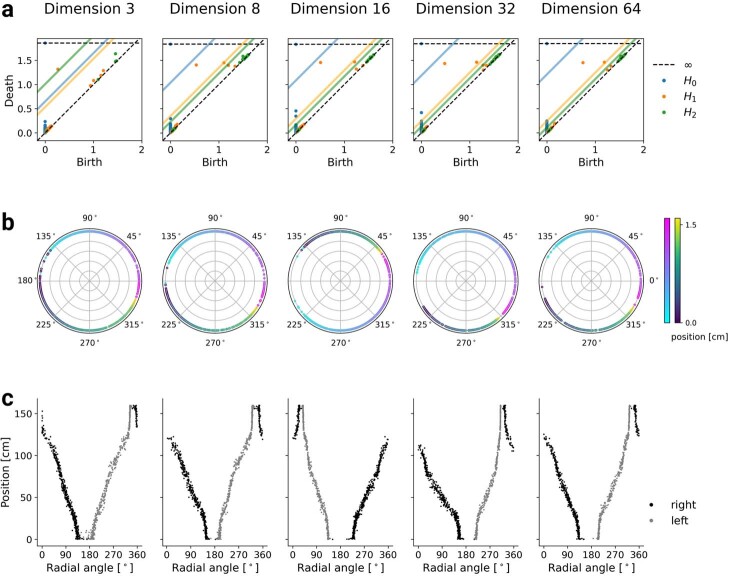
Persistence across dimensions. **a**, For each dimension of CEBRA-Behaviour embedding from the rat hippocampus dataset Betti numbers were computed by applying persistent cohomology. The coloured dots are lifespans observed in hypothesis based CEBRA-Behaviour. To rule out noisy lifespans, we set a threshold (coloured diagonal lines) as maximum lifespan based on 500 seeds of shuffled-CEBRA embedding for each dimension. **b**, The topology preserving circular coordinates using the first co-cycle from persistent cohomology analysis on the CEBRA embedding of each dimension is shown (see Methods). The colours indicate position and direction of the rat at the corresponding CEBRA embedding points. **c**, The radial angle of each embedding point obtained from **b** and the corresponding position and direction of the rat.

Although CEBRA can be trained across a range of dimensions, and models can be selected based on decoding, goodness of fit and consistency, we also sought to find a principled approach to verify the robustness of embeddings that might yield insight into neural computations^30,31^ (Fig. 2e). We used algebraic topology to measure the persistent cohomology as a comparison in regard to whether learned latent spaces are equivalent. Although it is not required to project embeddings onto a sphere, this has the advantage that there are default Betti numbers (for a *d*-dimensional uniform embedding, ${H}^{0}=1,{H}^{1}=0,\cdots ,{H}^{d-1}=1$—that is, 1,0,1 for the two-sphere). We used the distance from the unity line (and threshold based on a computed null shuffled distribution in Births versus Deaths to compute Betti numbers; Extended Data Fig. 6). Using CEBRA-Behaviour or -Time we find a ring topology (1,1,0; Fig. 2f), as one would expect from a linear track for place cells. We then computed the Eilenberg–MacLane coordinates for the identified cocycle (H^1^) for each model^32,33^—this allowed us to map each time point to topology-preserving coordinates—and indeed we find that the ring topology for the CEBRA models matches space (position) across dimensions (Fig. 2g and Extended Data Fig. 6). Note that this topology differs from (1,0,1)—that is, Betti numbers for a uniformly covered sphere—which in our setting would indicate a random embedding as found by shuffling (Fig. 2g).

## Multi-session, multi-animal CEBRA

**Extended Data Fig. 7:**
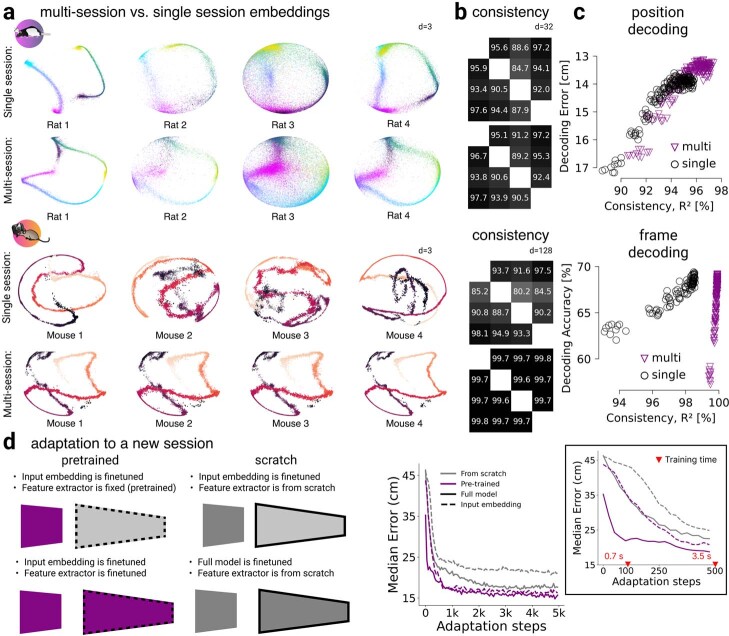
Multi-session training and rapid decoding. **a**, Top: hippocampus dataset, single animal vs. multi-animal training shows an increase in consistency across animals. Bottom: same for Allen dataset, 4 mice. **b**, Consistency matrix single vs. multi-session training for hippocampus (32D embedding) and Allen data (128D embedding) respectively. Consistency is reported at the point in training where the average position decoding error is less than 14 cm (corresponds to 7 cm error for rat 1), and a decoding accuracy of 60% on the Allen dataset. **c**, Comparison of decoding metrics for single or multi-session training at various consistency levels (averaged across all 12 comparisons). Models were trained for 5,000 (single) or 10,000 (multi-session) steps with a 0.003 learning rate; batch size was 7,200 samples per session. Multi-session training requires longer training or higher learning rates to obtain the same accuracy due to the 4-fold larger batch size, but converges to same decoding accuracy. We plot points at intervals of 500 steps (*n* = 10 seeds); training progresses from lower right to upper left corner within both plots. **d**, We demonstrate that we could also adapt to an unseen dataset; here, 3 rats were used for pretraining, and rat 4 was used as a held-out test. The grey lines indicate models trained from scratch (random initialization). We also tested fine-tuning only the input embedding (first layer) or the full model, as the diagram, left, describes. We measured the average time (mean ± s.d.) to adapt 100 steps (0.65 ± 0.13 s) and 500 steps (3.07 ± 0.61 s) on 40 repeated experiments.

CEBRA can also be used to jointly train across sessions and different animals, which can be highly advantageous when there is limited access to simultaneously recorded neurons or when looking for animal-invariant features in the neural data. We trained CEBRA across animals within each multi-animal dataset and find that this joint embedding allows for even more consistent embeddings across subjects (Extended Data Fig. 7a–c; one-sided, paired *t*-tests; Allen data: *t* = −5.80, *P* = 5.99 × 10^−5^; hippocampus: *t* = −2.22, *P* = 0.024).

Although consistency increased, it is not a priori clear that decoding from ‘pseudosubjects’ would be equally good because there could be session- or animal-specific information that is lost in pseudodecoding (because decoding is usually performed within the session). Alternatively, if this joint latent space was as high performance as the single subject, that would suggest that CEBRA is able to produce robust latent spaces across subjects. Indeed, we find no loss in decoding performance (Extended Data Fig. 7c).

It is also possible to rapidly decode from a new session that is unseen during training, which is an attractive setting for brain machine interface deployment. We show that, by pretraining on a subset of the subjects, we can apply and rapidly adapt CEBRA-Behaviour on unseen data (that is, it runs at 50–100 steps s^–1^, and positional decoding error already decreased by 10 cm after adapting the pretrained network for one step). Lastly, we can achieve a lower error more rapidly compared with training fully on the unseen individual (Extended Data Fig. 7d). Collectively, this shows that CEBRA can rapidly produce high-performance, consistent and robust latent spaces.

## Latent dynamics during a motor task

**Fig. 3:**
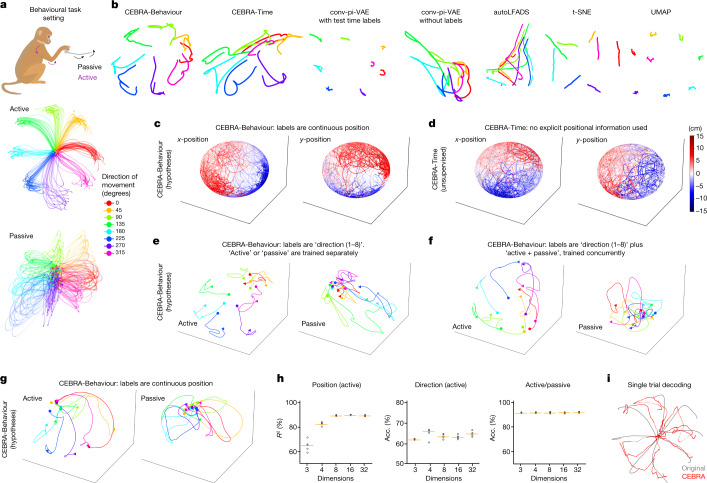
Forelimb movement behaviour in a primate. **a**, The monkey makes either active or passive movements in eight directions. Data derived from area 2 of primary S1 from Chowdhury et al.^34^. Cartoon from scidraw.io. **b**, Embeddings of active trials generated with 4D CEBRA-Behaviour, 4D CEBRA-Time, 4D autoLFADS, 4D conv-pi-VAE variants, 2D *t*-SNE and 2D UMAP. The embeddings of trials (*n* = 364) for each direction are post hoc averaged. **c**, CEBRA-Behaviour trained with (*x*,*y*) position of the hand. Left, colour coded to *x* position; right, colour coded to *y* position. **d**, CEBRA-Time with no external behaviour variables. Colour coded as in **c**. **e**, CEBRA-Behaviour embedding trained using a 4D latent space, with discrete target direction as behaviour labels, trained and plotted separately for active and passive trials. **f**, CEBRA-Behaviour embedding trained using a 4D latent space, with discrete target direction and active and passive trials as auxiliary labels plotted separately, active versus passive trials. **g**, CEBRA-Behaviour embedding trained with a 4D latent space using active and passive trials with continuous (*x*,*y*) position as auxiliary labels plotted separately, active versus passive trials. The trajectory of each direction is averaged across trials (*n* = 18–30 each, per direction) over time. Each trajectory represents 600 ms from −100 ms before the start of the movement. **h**, Left to right, decoding performance of: position using CEBRA-Behaviour trained with (*x*,*y*) position (active trials); target direction using CEBRA-Behaviour trained with target direction (active trials); and active versus passive accuracy (Acc.) using CEBRA-Behaviour trained with both active and passive movements. In each case we trained and evaluated five seeds, represented by black dots; orange line represents the median. **i**, Decoded trajectory of hand position using CEBRA-Behaviour trained on active trial with (*x*,*y*) position of the hand. The grey line represents a true trajectory and the red line represents a decoded trajectory.

**Extended Data Fig. 8:**
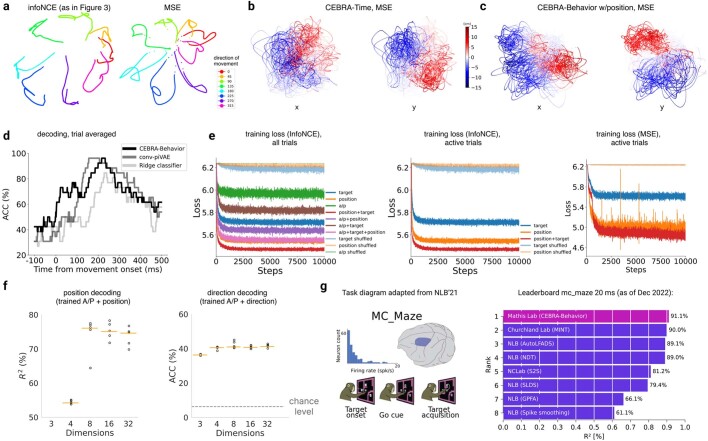
Somatosensory cortex decoding from primate recordings. **a**, We compare CEBRA-Behaviour with the cosine similarity and embeddings on the sphere reproduced from Fig. 3b (left) against CEBRA-Behaviour trained with the MSE loss and unnormalized embeddings. The embeddings of trials (*n* = 364) of each direction were post-hoc averaged. **b**, CEBRA-Behaviour trained with *x,y* position of the hand. Left panel is colour- coded to changes in *x* position and right panel is color-coded to changes in *y* position. **c**, CEBRA-Time without any external auxiliary variables. As in **b**, left and right are colour-coded to *x* and *y* position, respectively. **d**, Decoding performance of target direction using CEBRA-Behaviour, conv-pi-VAE and a linear classifier. CEBRA-Behaviour shows significantly higher decoding performance than the linear classifier (one-way ANOVA, *F*(2,75) = 3.37, *P* < 0.05 with Post Hoc Tukey significant difference *P*< 0.05). **e**, Loss (InfoNCE) vs. training iteration for CEBRA-Behaviour with position, direction, active or passive, and position+direction labels (and shuffled labels) for all trials (left) or only active trials (right), or active trials with a MSE loss. **f**, Additional decoding performance results on position and direction-trained CEBRA models with all trial types. For each case, we trained and evaluated 5 seeds represented by black dots and the orange line represents the median. **g**, Results on the mc-maze 20 ms benchmark.

We next consider an eight-direction ‘centre-out’ reaching task paired with electrophysiology recordings in primate somatosensory cortex (S1)^34^ (Fig. 3a). The monkey performed many active movements, and in a subset of trials experienced randomized bumps that caused passive limb movement. CEBRA produced highly informative visualizations of the data compared with other methods (Fig. 3b), and CEBRA-Behaviour can be used to test the encoding properties of S1. Using either position or time information showed embeddings with clear positional encoding (Fig. 3c,d and Extended Data Fig. 8a–c).

To test how directional information and active versus passive movements influence population dynamics in S1 (refs. ^34–36^), we trained embedding spaces with directional information and then either separated the trials into active and passive for training (Fig. 3e) or trained jointly and post hoc plotted separately (Fig. 3f). We find striking similarities suggesting that active versus passive strongly influences the neural latent space: the embeddings for active trials show a clear start and stop whereas for passive trials they show a continuous trajectory through the embedding, independently of how they are trained. This finding is confirmed in embeddings that used only the continuous position of the end effector as the behavioural label (Fig. 3g). Notably, direction is a less prominent feature (Fig. 3g) although they are entangled parameters in this task.

As the position and active or passive trial type appear robust in the embeddings, we further explored the decodability of the embeddings. Both position and trial type were readily decodable from 8D+ embeddings with a kNN decoder trained on position only, but directional information was not as decodable (Fig. 3h). Here too, the loss function value is informative for goodness of fit during hypothesis testing (Extended Data Fig. 8d–f). Notably, we could recover the hand trajectory with *R*^2^ = 88% (concatenated across 26 held-out test trials; Fig. 3i) using a 16D CEBRA-Behaviour model trained on position (Fig. 3i). For comparison, an L1 regression using all neurons achieved *R*^2^ = 74% and 16D conv-pi-VAE achieved *R*^2^ = 82%. We also tested CEBRA on an additional monkey dataset (mc-maze) presented in the Neural Latent Benchmark^37^, in which it achieved state-of-the-art behaviour (velocity) decoding performance (Extended Data Fig. 8).

## Consistent embeddings across modalities

Although CEBRA is agnostic to the recording modality of neural data, do different modalities produce similar latent embeddings? Understanding the relationship of calcium signalling and electrophysiology is a debated topic, yet an underlying assumption is that they inherently represent related, yet not identical, information. Although there is a wealth of excellent tools aimed at inferring spike trains from calcium data, currently the pseudo-*R*^2^ of algorithms on paired spiking and calcium data tops out at around 0.6 (ref. ^38^). Nonetheless, it is clear that recording with either modality has led to similar global conclusions—for example, grid cells can be uncovered in spiking or calcium signals^33,39^, reward prediction errors can be found in dopamine neurons across species and recording modalities^40–42^, and visual cortex shows orientation tuning across species and modalities^43–45^.

**Fig. 4:**
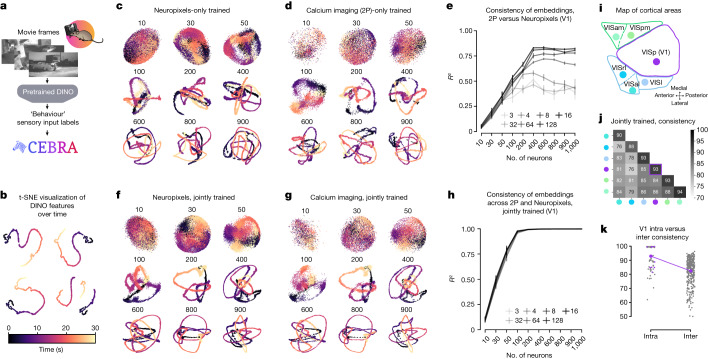
Spikes and calcium signalling show similar CEBRA embeddings. **a**, CEBRA-Behaviour can use frame-by-frame video features as a label of sensory input to extract the neural latent space of the visual cortex of mice watching a video. Cartoon from scidraw.io. **b**, *t*-SNE visualization of the DINO features of video frames from four different DINO configurations (latent size, model size), all showing continuous evolution of video frames over time. **c**,**d**, Visualization of trained 8D latent CEBRA-Behaviour embeddings with Neuropixels (NP) data (**c**) or calcium imaging (2P) (**d**). Numbers above each embedding indicate neurons subsampled from the multi-session concatenated dataset. Colour map as in **b**. **e**, Linear consistency between embeddings trained with either calcium imaging or Neuropixels data (*n* = 10–1,000 neurons, across *n*  =  5 shuffles of neural data; mean values ± s.e.m.). **f**,**g**, Visualization of CEBRA-Behaviour embedding (8D) trained jointly with Neuropixels (**f**) and calcium imaging (**g**). Colour map as in **b**. **h**, Linear consistency between embeddings of calcium imaging and Neuropixels trained jointly using a multi-session CEBRA model (*n* = 10–1000 neurons, across *n* = 5 shuffles of neural data; mean values ±  s.e.m.). **i**, Diagram of mouse primary visual cortex (V1, VISp) and higher visual areas. **j**, CEBRA-Behaviour 32D model jointly trained with 2P + NP incorporating 400 neurons, followed by measurement of consistency within or across areas (2P versus NP) across two unique sets of disjoint neurons for three seeds and averaged. **k**, Models trained as in **h**, with intra-V1 consistency measurement versus all interarea versus V1 comparison. Purple dots indicate mean of V1 intra-V1 consistency (across *n* = 120 runs) and inter-V1 consistency (*n* = 120 runs). Intra-V1 consistency is significantly higher than interarea consistency (one-sided Welch’s *t*-test, *t*(12.30) = 4.55, *P* = 0.00019).

We aimed to formally study whether CEBRA could capture the same neural population dynamics either from spikes or calcium imaging. We utilized a dataset from the Allen Brain Observatory where mice passively watched three videos repeatedly. We focused on paired data from ten repeats of ‘Natural Movie 1’ where neural data were recorded with either Neuropixels (NP) probes or calcium imaging with a two-photon (2P) microscope (from separate mice)^46,47^. Note that, although the data we have considered thus far have goal-driven actions of the animals (such as running down a linear track or reaching for targets), this visual cortex dataset was collected during passive viewing (Fig. 4a).

**Extended Data Fig. 9:**
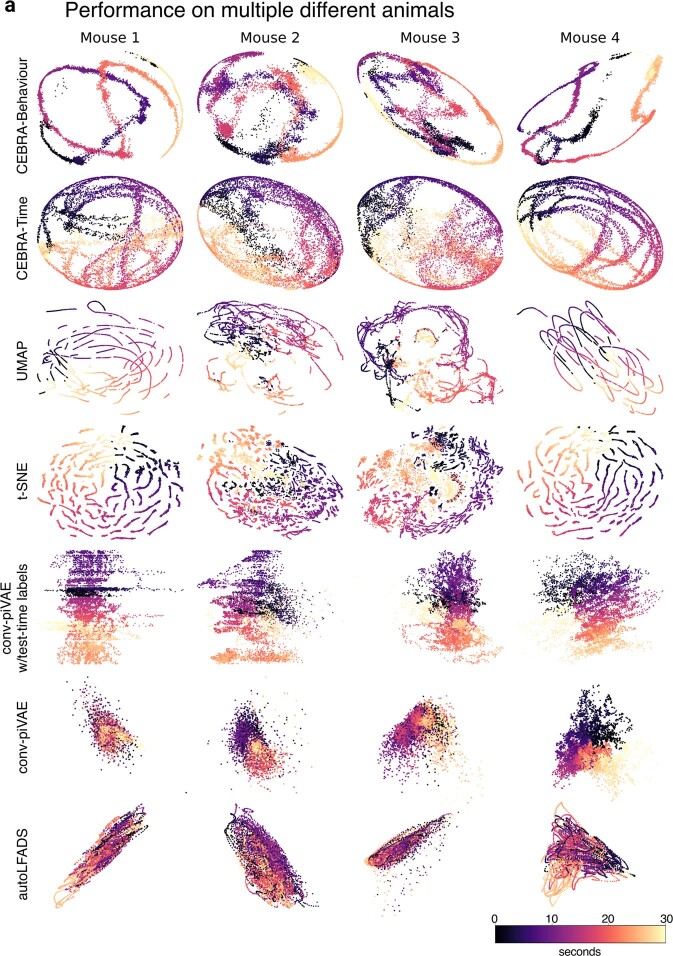
CEBRA produces consistent, highly decodable embeddings. **a**, Additional 4 sessions with the most neurons in the Allen visual dataset calcium recording shown for all algorithms we benchmarked (see Methods). For CEBRA-Behaviour and CEBRA-Time, we used 3D, temperature 1, time offset 10, batch size 512 and 10k training steps. For UMAP, we used a cosine metric and *n*_*neighbors* 15 and *min*_*dist* 0.1. For t-SNE, we used a cosine metric and *perplexity 30*. For conv-pi-VAE, we trained with 600 epochs, a batch size of 200 and a learning rate 5 x 10^*−*4^. autoLFADS was trained with ray-tune parameter selection and the resulting factors were transformed with PCA to generate the visualization. All methods used 10-time-bins input. CEBRA was trained with 3D latent and all other methods were obtained with an equivalent 2D latent dimension. To align for visualization, we aligned to mouse 1, except for conv-pi-VAE without labels and for autoLFADS, which visually looked best when aligned to mouse 4.

We used the video features as ‘behaviour’ labels by extracting high-level visual features from the video on a frame-by-frame basis with DINO, a powerful vision transformer model^48^. These were then used to sample the neural data with feature-labels (Fig. 4b). Next, we used either Neuropixels or 2P data (each with multi-session training) to generate (from 8D to 128D) latent spaces from varying numbers of neurons recorded from primary visual cortex (V1) (Fig. 4c,d). Visualization of CEBRA-Behaviour showed trajectories that smoothly capture the video of either modality with an increasing number of neurons. This is reflected quantitatively in the consistency metric (Fig. 4e). Strikingly, CEBRA-Time efficiently captured the ten repeats of the video (Extended Data Fig. 9), which was not captured by other methods. This result demonstrates that there is a highly consistent latent space independent of the recording method.

**Extended Data Fig. 10:**
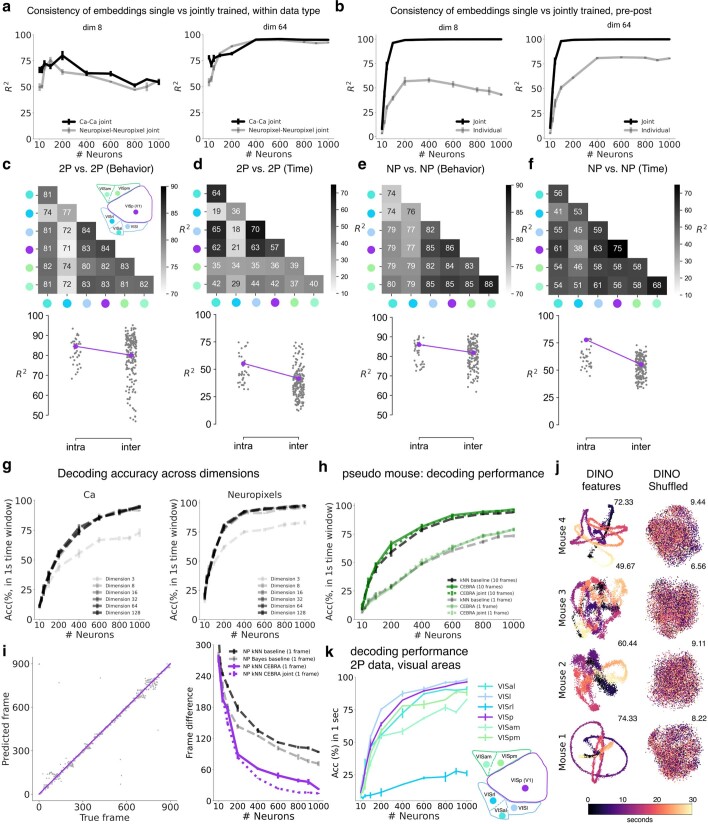
Spikes and calcium signalling reveal similar embeddings. **a**, Consistency between the single and jointly trained embeddings. **b**, Consistency of embeddings from two recording modalities, when a single modality was trained independently and/or jointly trained. CEBRA can find ‘common latents’ even without joint training. Data is also presented in Fig. 4e, h, but here plotted together to show improvement with joint training; for **a** and **b**, for each neuron number we have *n* = 5 shuffles, mean ± s.e.m. **c**-**f**, Consistency across modalities and areas for CEBRA-Behaviour and -Time (as computed in Fig. 4i–k). The purple dots indicate mean of intra-V1 scores and inter-V1 scores (inter-V1 vs intra-V1 one-sided Welch’s t-test; 2P (Behaviour): *t*(10.6) = 1.52, *P* = 0.081, 2P (Time): *t*(44.3) = 4.26, *P* = 0.0005, NP (Behaviour): *t*(11.6) = 2.83, *P* = 0.0085, NP (Time): *t*(8.9) = 15.51, *P* < 0.00001) **g**, CEBRA + kNN decoding performance (see Methods) of CEBRA embeddings of different output embedding dimensions, from calcium (2P) data or Neuropixels (NP), as denoted; for each neuron number we have *n* = 5 shuffles, mean  ± s.e.m. **h**, Decoding accuracy measured by considering predicted frame being within 1 s difference to true frame using CEBRA (2P only), jointly trained (2P+NP), or a baseline population vector kNN decoder (using time window 33 ms (single frame), or 330 ms (10 frame receptive field)); for each neuron number we have *n *= 5 shuffles, mean  ± s.e.m. (**i**): Single frame performance and quantification using CEBRA 1 frame receptive field (NP data), or baseline models, *n* = 900 video frames. **j**, CEBRA-Behaviour used the DINO features as auxiliary labels and DINO-shuffled used the shuffled DINO features. We shuffled the frame order of DINO features within a repeat. Same shuffled order was use for all repeats. Colour code is frame number from the movie. The prediction is considered as true if the predicted frame is within 1 s from the true frame, and the accuracy (%) is noted next to the embedding. For mouse ID 1–4: 337, 353, 397, 475 neurons were recorded, respectively. **k**, Decoding performance from 2P data from different visual cortical areas from different layers using a 10-frame-window, 128D CEBRA-Behaviour model using DINO features; for each neuron number we have *n* = 5 shuffles, mean± s.e.m.

Next, we stacked neurons from different mice and modalities and then sampled random subsets of V1 neurons to construct a pseudomouse. We did not find that joint training lowered consistency within modality (Extended Data Fig. 10a,b) and, overall, we found considerable improvement in consistency with joint training (Fig. 4f–h).

Using CEBRA-Behaviour or -Time, we trained models on five higher visual areas and measured consistency with and without joint training, and within or across areas. Our results show that, with joint training, intra-area consistency is higher compared with other areas (Fig. 4i–k), suggesting that CEBRA is not removing biological differences across areas, which have known differences in decodability and feature representations^49,50^. Moreover, we tested within modality and find a similar effect for CEBRA-Behaviour and -Time within recording modality (Extended Data Fig. 10c–f).

## Decoding of natural videos from cortex

**Fig. 5:**
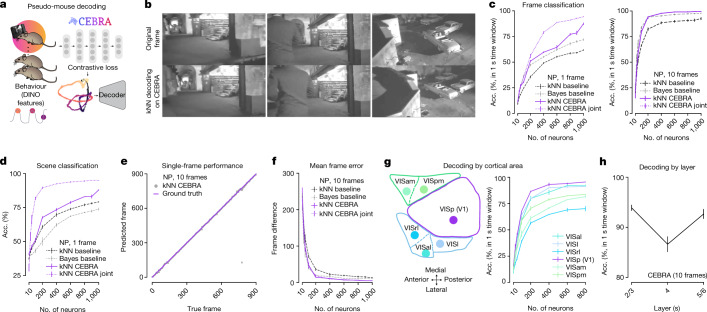
Decoding of natural video features from mouse visual cortical areas. **a**, Schematic of the CEBRA encoder and kNN (or naive Bayes) decoder. **b**, Examples of original frames (top row) and frames decoded from CEBRA embedding of V1 calcium recording using kNN decoding (bottom row). The last repeat among ten was used as the held-out test. **c**, Decoding accuracy measured by considering a predicted frame being within 1 s of the true frame as a correct prediction using CEBRA (NP only), jointly trained (2P + NP) or a baseline population vector plus kNN or naive Bayes decoder using either a one-frame (33 ms) receptive field (left) or ten frames (330 ms) (right); results shown for Neuropixels dataset (V1 data); for each neuron number we have *n* = 5 shuffles, mean ± s.e.m. **d**, Decoding accuracy measured by correct scene prediction using either CEBRA (NP only), jointly trained (2P + NP) or baseline population vector plus kNN or Bayes decoder using a one-frame (33 ms) receptive field (V1 data); *n* = 5 shuffles per neuron number, mean ± s.e.m. **e**, Single-frame ground truth frame ID versus predicted frame ID for Neuropixels using a CEBRA-Behaviour model trained with a 330 ms receptive field (1,000 V1 neurons across mice used). **f**, Mean absolute error of the correct frame index; shown for baseline and CEBRA models as computed in **c**–**e**. **g**, Diagram of the cortical areas considered and decoding performance from CEBRA (NP only), ten-frame receptive field; *n* = 3 shuffles for each area and number of neurons, mean ± s.e.m. **h**, V1 decoding performance versus layer category using 900 neurons with a 330 ms receptive field CEBRA-Behaviour model; *n* = 5 shuffles for each layer, mean ± s.e.m.

We performed V1 decoding analysis using CEBRA models that are either joint-modality trained, single-modality trained or with a baseline population vector paired with a simple kNN or naive Bayes decoder. We aimed to determine whether we could decode, on a frame-by-frame basis, the natural video watched by the mice. We used the final video repeat as a held-out test set and nine repeats as the training set. We achieved greater than 95% decoding accuracy, which is significantly better than baseline decoding methods (naive Bayes or kNN) for Neuropixels recordings, and joint-training CEBRA outperformed Neuropixels-only CEBRA-based training (single frame: one-way ANOVA, *F*(3,197) = 5.88, *P* = 0.0007; Supplementary Tables 3–5, Fig. 5a–d and Extended Data Fig. 10g,h). Accuracy was defined by either the fraction of correct frames within a 1 s window or identification of the correct scene. Frame-by-frame results also showed reduced frame ID errors (one-way ANOVA, *F*(3,16) = 20.22, *P* = 1.09 × 10^−5^, *n* = 1,000 neurons; Supplementary Table 6), which can be seen in Fig. 5e,f, Extended Data Fig. 10i and Supplementary Video 2. The DINO features themselves did not drive performance, because shuffling of features showed poor decoding (Extended Data Fig. 10j).

Lastly, we tested decoding from other higher visual areas using DINO features. Overall, decoding from V1 had the highest performance and VISrl the lowest (Fig. 5g and Extended Data Fig. 10k). Given the high decoding performance of CEBRA, we tested whether there was a particular V1 layer that was most informative. We leveraged CEBRA-Behaviour by training models on each category and found that layers 2/3 and 5/6 showed significantly higher decoding performance compared with layer 4 (one-way ANOVA, *F*(2,12) = 9.88, *P* = 0.003; Fig. 5h). Given the known cortical connectivity, this suggests that the nonthalamic input layers render frame information more explicit, perhaps via feedback or predictive processing.

## Discussion

CEBRA is a nonlinear dimensionality reduction method newly developed to explicitly leverage auxiliary (behaviour) labels and/or time to discover latent features in time series data—in this case, latent neural embeddings. The unique property of CEBRA is the extension and generalization of the standard InfoNCE objective by introduction of a variety of different sampling strategies tuned for usage of the algorithm in the experimental sciences and for analysis of time series datasets, and it can also be used for supervised and self-supervised analysis, thereby directly facilitating hypothesis- and discovery-driven science. It produces both consistent embeddings across subjects (thus showing common structure) and can find the dimensionality of neural spaces that are topologically robust. Although there remains a gap in our understanding of how these latent spaces map to neural-level computations, we believe this tool provides an advance in our ability to map behaviour to neural populations. Moreover, because pretrained CEBRA models can be used for decoding in new animals within tens of steps (milliseconds), we can thereby obtain equal or better performance compared with training on the unseen animal alone.

Dimensionality reduction is often tightly linked to data visualization, and here we make an empirical argument that ultimately this is useful only when obtaining consistent results and discovering robust features. Unsupervised *t*-SNE and UMAP are examples of algorithms widely used in life sciences for discovery-based analysis. However, they do not leverage time and, for neural recordings, this is always available and can be used. Even more critical is that concatenation of data from different animals can lead to shifted clusters with *t*-SNE or UMAP due to inherent small changes across animals or in how the data were collected. CEBRA allows the user to remove this unwanted variance and discover robust latents that are invariant to animal ID, sessions or any-other-user-defined nuisance variable. Collectively we believe that CEBRA will become a complement to (or replacement for) these methods such that, at minimum, the structure of time in the neural code is leveraged and robustness is prioritized.

## Methods

### Datasets

#### Artificial spiking dataset

The synthetic spiking data used for benchmarking in Fig. 1 were adopted from Zhou and Wei^5^. The continuous 1D behaviour variable $c\in [0,2\pi )$ was sampled uniformly in the interval $[0,2\pi )$. The true 2D latent variable ${\bf{z}}\in {{\mathbb{R}}}^{2}$ was then sampled from a Gaussian distribution ${\mathscr{N}}\left(\mu \left(c\right),\varSigma \left(c\right)\right)$ with mean $\mu \left(c\right)={\left(c,2\sin c\right)}^{\top }$ and covariance $\varSigma \left(c\right)={\rm{diag}}\left(0.6-0.3\left|\sin c\right|,0.3\left|\sin c\right|\right)$. After sampling, the 2D latent variable ${\bf{z}}$ was mapped to the spiking rates of 100 neurons by the application of four randomly initialized RealNVP^51^ blocks. Poisson noise was then applied to map firing rates onto spike counts. The final dataset consisted of 1.5 × 10^4^ data points for 100 neurons ([number of samples, number of neurons]) and was split into train (80%) and validation (20%) sets. We quantified consistency across the entire dataset for all methods. Additional synthetic data, presented in Extended Data Fig. 1, were generated by varying noise distribution in the above generative process. Beside Poisson noise, we used additive truncated ([0,1000]) Gaussian noise with s.d. = 1 and additive uniform noise defined in [0,2], which was applied to the spiking rate. We also adapted Poisson spiking by simulating neurons with a refractory period. For this, we scaled the spiking rates to an average of 110 Hz. We sampled interspike intervals from an exponential distribution with the given rate and added a refractory period of 10 ms.

#### Rat hippocampus dataset

We used the dataset presented in Grosmark and Buzsáki^26^. In brief, bilaterally implanted silicon probes recorded multicellular electrophysiological data from CA1 hippocampus areas from each of four male Long–Evans rats. During a given session, each rat independently ran on a 1.6-m-long linear track where they were rewarded with water at each end of the track. The numbers of recorded putative pyramidal neurons for each rat ranged between 48 and 120. Here, we processed the data as in Zhou and Wei^5^. Specifically, the spikes were binned into 25 ms time windows. The position and running direction (left or right) of the rat were encoded into a 3D vector, which consisted of the continuous position value and two binary values indicating right or left direction. Recordings from each rat were parsed into trials (a round trip from one end of the track as a trial) and then split into train, validation and test sets with a *k* = 3 nested cross-validation scheme for the decoding task.

#### Macaque dataset

We used the dataset presented in Chowdhury et al.^34^ In brief, electrophysiological recordings were performed in Area 2 of somatosensory cortex (S1) in a rhesus macaque (monkey) during a centre-out reaching task with a manipulandum. Specifically, the monkey performed an eight-direction reaching task in which on 50% of trials it actively made centre-out movements to a presented target. The remaining trials were ‘passive’ trials in which an unexpected 2 Newton force bump was given to the manipulandum towards one of the eight target directions during a holding period. The trials were aligned as in Pei et al.^37^, and we used the data for −100 and 500 ms from movement onset. We used 1 ms time bins and convolved the data using a Gaussian kernel with s.d. = 40 ms.

#### Mouse visual cortex datasets

We utilized the Allen Institute two-photon calcium imaging and Neuropixels data recorded from five mouse visual and higher visual cortical areas (VISp, VISl, VISal, VISam, VISpm and VISrl) during presentation of a monochrome video with 30 Hz frame rate, as presented previously^46,47,52^. For calcium imaging (2P) we used the processed dataset from Vries et al.^46^ with a sampling rate of 30 Hz, aligned to the video frames. We considered the recordings from excitatory neurons (Emx1-IRES-Cre, Slc17a7-IRES2-Cre, Cux2-CreERT2, Rorb-IRES2-Cre, Scnn1a-Tg3-Cre, Nr5a1-Cre, Rbp4-Cre_KL100, Fezf2-CreER and Tlx3-Cre_PL56) in the ‘Visual Coding-2P’ dataset. Ten repeats of the first video (Movie 1) were shown in all session types (A, B and C) for each mouse and we used those neurons that were recorded in all three session types, found via cell registration^46^. The Neuropixels recordings were obtained from the ‘Brain Observatory 1.1’ dataset^47^. We used the preprocessed spike timings and binned them to a sampling frequency of 120 Hz, aligned with the video timestamps (exactly four bins aligned with each frame). The dataset contains recordings for ten repeats, and we used the same video (Movie 1) that was used for the 2P recordings. For analysis of consistency across the visual cortical areas we used a disjoint set of neurons for each seed, to avoid higher intraconsistency due to overlapping neuron identities. We made three disjoint sets of neurons by considering only neurons from session A (for 2P data) and nonoverlapping random sampling for each seed.

### CEBRA model framework

#### Notation

We will use **x**,**y** as general placeholder variables and denote the multidimensional, time-varying signal as **s**_*t*_, parameterized by time *t*. The multidimensional, continuous context variable **c**_*t*_ contains additional information about the experimental condition and additional recordings, similar to the discrete categorical variable *k*_*t*_.

The exact composition of **s**, **c** and *k* depends on the experimental context. CEBRA is agnostic to exact signal types; with the default parameterizations, **s**_*t*_ and **c**_*t*_ can have up to an order of hundreds or thousands of dimensions. For even higher-dimensional datasets (for example, raw video, audio and so on) other optimized deep learning tools can be used for feature extraction before the application of CEBRA.

#### Applicable problem setup

We refer to ${\bf{x}}\in X$ as the reference sample and to ${\bf{y}}\in Y$ as a corresponding positive or negative sample. Together, (**x**, **y**) form a positive or negative pair based on the distribution from which **y** is sampled. We denote the distribution and density function of **x** as *p*(**x**), the conditional distribution and density of the positive sample **y** given **x** as $p({\bf{y}}{\rm{| }}{\bf{x}})$ and the conditional distribution and density of the negative sample **y** given **x** as $q\left({\bf{y}}{\rm{| }}{\bf{x}}\right)$.

After sampling—and irrespective of whether we are considering a positive or negative pair—samples ${\bf{x}}\in {{\mathbb{R}}}^{D}$ and ${\bf{y}}\in {{\mathbb{R}}}^{{D}^{{\prime} }}$ are encoded by feature extractors ${\bf{f}}:X\mapsto Z$ and ${{\bf{f}}}^{{\prime} }:Y\mapsto Z$. The feature extractors map both samples from signal space $X\subseteq {{\mathbb{R}}}^{D},Y\subseteq {{\mathbb{R}}}^{{D}^{{\prime} }}$ into a common embedding space $Z\subseteq {{\mathbb{R}}}^{E}$. The design and parameterization of the feature extractor are chosen by the user of the algorithm. Note that spaces *X* and *Y* and their corresponding feature extractors can be the same (which is the case for single-session experiments in this work), but that this is not a strict requirement within the CEBRA framework (for example, in multi-session training across animals or modalities, *X* and *Y* are selected as signals from different mice or modalities, respectively). It is also possible to include the context variable (for example, behaviour) into *X*, or to set **x** to the context variable and **y** to the signal variable.

Given two encoded samples, a similarity measure $\varphi :Z\times Z\mapsto {\mathbb{R}}$ assigns a score to a pair of embeddings. The similarity measure needs to assign a higher score to more similar pairs of points, and to have an upper bound. For this work we consider the dot product between normalized feature vectors, $\varphi ({\bf{z}},{{\bf{z}}}^{{\prime} })={{\bf{z}}}^{{\rm{\top }}}{{\bf{z}}}^{{\prime} }/\tau$, in most analyses (latents on a hypersphere) or the negative mean squared error, $\varphi ({\bf{z}},{{\bf{z}}}^{{\prime} })=-\,\parallel {\bf{z}}-{{\bf{z}}}^{{\prime} }{\parallel }^{2}/\tau$ (latents in Euclidean space). Both metrics can be scaled by a temperature parameter *τ* that is either fixed or jointly learned with the network. Other *L*_*p*_ norms and other similarity metrics, or even a trainable neural network (a so-called projection head commonly used in contrastive learning algorithms^14,22^), are possible choices within the CEBRA software package. The exact choice of *ϕ* shapes the properties of the embedding space and encodes assumptions about distributions *p* and *q*.

The technique requires paired data recordings—for example, as is common in aligned time series. The signal **s**_*t*_, continuous context **c**_*t*_ and discrete context k_*t*_ are synced in their time point *t*. How the reference, positive and negative samples are constructed from these available signals is a configuration choice made by the algorithm user, and depends on the scientific question under investigation.

#### Optimization

Given the feature encoders **f** and **f**′ for the different sample types, as well as the similarity measure *ϕ*, we introduce the shorthand $\psi \left({\bf{x}},{\bf{y}}\right)=\varphi \left({\bf{f}}\left({\bf{x}}\right),{{\bf{f}}}^{{\prime} }\left({\bf{y}}\right)\right)$. The objective function can then be compactly written as:

1 $$\mathop{\int }\limits_{{\bf{x}}\in {\rm{X}}}{\rm{d}}{\bf{x}}{\rm{p}}({\bf{x}})\,[\,-\mathop{\int }\limits_{{\bf{y}}\in {\rm{Y}}}{\rm{d}}{\bf{y}}{\rm{p}}({\bf{y}}|{\bf{x}})\psi ({\bf{x}},{\bf{y}})+\log \mathop{\int }\limits_{{\bf{y}}\in {\rm{Y}}}{\rm{d}}{\bf{y}}{\rm{q}}({\bf{y}}|{\bf{x}}){{\rm{e}}}^{\psi ({\bf{x}},{\bf{y}})}].$$

We approximate this objective by drawing a single positive example **y**_+_, and multiple negative examples **y**_*i*_ from the distributions outlined above, and minimize the loss function

2 $$\mathop{{\mathbb{E}}}\limits_{\begin{array}{c}{\bf{x}}\sim p({\bf{x}}),{{\bf{y}}}_{+}\sim p({\bf{y}}|{\bf{x}})\\ {{\bf{y}}}_{1},\ldots ,{{\bf{y}}}_{n}\sim q({\bf{y}}|{\bf{x}})\end{array}}\,[-\psi ({\bf{x}},{{\bf{y}}}_{+})+\,\log \mathop{\sum }\limits_{i=1}^{n}{e}^{\psi ({\bf{x}},{{\bf{y}}}_{i})}],$$

with a gradient-based optimization algorithm. The number of negative samples is a hyperparameter of the algorithm, and larger batch sizes are generally preferable.

For sufficiently small datasets, as used in this paper, both positive and negative samples are drawn from all available samples in the dataset. This is in contrast to the common practice in many contrastive learning frameworks in which a minibatch of samples is drawn first, which are then grouped into positive and negative pairs. Allowing access to the whole dataset to form pairs gives a better approximation of the respective distributions $p\left({\bf{y}}{\rm{| }}{\bf{x}}\right)$ and $q\left({\bf{y}}{\rm{| }}{\bf{x}}\right)$, and considerably improves the quality of the obtained embeddings. If the dataset is sufficiently small to fit into the memory, CEBRA can be optimized with batch gradient descent—that is, using the whole dataset at each optimizer step.

#### Goodness of fit

Comparing the loss value—at both absolute and relative values across models at the same point in training time— can be used to determine goodness of fit. In practical terms, this means that one can find which hypothesis best fits one’s data, in the case of using CEBRA-Behaviour. Specifically, let us denote the objective in equation(1) as *L*_asympt_ and its approximation in equation (2) with a batch size of *n* as *L*_*n*_. In the limit of many samples, the objective converges up to a constant, ${L}_{{\rm{asympt}}}={{\rm{lim}}}_{n\to \infty }\left[{L}_{n}-\log n\right]$ (Supplementaty Note 2 and ref. ^53^).

The objective also has two trivial solutions: the first is obtained for a constant $\psi \left({\bf{x}},{\bf{y}}\right)=\psi$, which yields a value of *L*_*n*_ = log*n*. This solution can be obtained when the labels are not related to the signal (e.g., with shuffled labels). It is typically not obtained during regular training because the network is initialized randomly, causing the initial embedding points to be randomly distributed in space.

If the embedding points are distributed uniformly in space and *ϕ* is selected such that ${\mathbb{E}}\left[\varphi \left({\bf{x}},{\bf{y}}\right)\right]=0$, we will also get a value that is approximately *L*_*n*_  =  log*n*. This value can be readily estimated by computing $\varphi \left({\bf{u}},{\bf{v}}\right)$ for randomly distributed points.

The minimizer of equation (1) is also clearly defined as ${-D}_{{\rm{KL}}}\left(p\parallel q\right)$ and depends on the positive and negative distribution. For discovery-driven (time contrastive) learning, this value is impossible to estimate because it would require access to the underlying conditional distribution of the latents. However, for training with predefined positive and negative distributions, this quantity can be again numerically estimated.

Interesting values of the loss function when fitting a CEBRA model are therefore

3 $$-{D}_{{\rm{KL}}}\left(p\parallel q\right)\le {L}_{n}-\log n\,\le 0$$

where *L*_*n*_ – log*n* is the goodness of fit (lower is better) of the CEBRA model. Note that the metric is independent of the batch size used for training.

#### Sampling

Selection of the sampling scheme is CEBRA’s key feature in regard to adapting embedding spaces to different datasets and recording setups. The conditional distributions $p\left({\bf{y}}{\rm{| }}{\bf{x}}\right)$ for positive samples and $q\left({\bf{y}}{\rm{| }}{\bf{x}}\right)$ for negative samples, as well as the marginal distribution *p*(**x**) for reference samples, are specified by the user. CEBRA offers a set of predefined sampling techniques but customized variants can be specified to implement additional, domain-specific distributions. This form of training allows the use of context variables to shape the properties of the embedding space, as outlined in the graphical model in Supplementary Note 1.

Through the choice of sampling technique, various use cases can be built into the algorithm. For instance, by forcing positive and negative distributions to sample uniformly across a factor, the model will become invariant to this factor because its inclusion would yield a suboptimal value of the objective function.

When considering different sampling mechanisms we distinguish between single- and multi-session datasets: a single-session dataset consists of samples **s**_*t*_ associated to one or more context variables **c**_*t*_ and/or *k*_*t*_. These context variables allow imposition of the structure on the marginal and conditional distribution used for obtaining the embedding. Multi-session datasets consist of multiple, single-session datasets. The dimension of context variables **c**_*t*_ and/or *k*_*t*_ must be shared across all sessions whereas the dimension of the signal **s**_*t*_ can vary. In such a setting, CEBRA allows learning of a shared embedding space for signals from all sessions.

For single-session datasets, sampling is done in two steps. First, based on a specified ‘index’ (the user-defined context variable **c**_*t*_ and/or *k*_*t*_), locations *t* are sampled for reference, positive and negative samples. The algorithm differentiates between categorical (*k*) and continuous (**c**) variables for this purpose.

In the simplest case, negative sampling (*q*) returns a random sample from the empirical distribution by returning a randomly chosen index from the dataset. Optionally, with a categorical context variable ${k}_{t}\in \left[K\right]$, negative sampling can be performed to approximate a uniform distribution of samples over this context variable. If this is performed for both negative and positive samples, the resulting embedding will become invariant with respect to the variable *k*_*t*_. Sampling is performed in this case by computing the cumulative histogram of *k*_*t*_ and sampling uniformly over *k* using the transformation theorem for probability densities.

For positive pairs, different options exist based on the availability of continuous and discrete context variables. For a discrete context variable ${k}_{t}\in \left[K\right]$ with *K* possible values, sampling from the conditional distribution is done by filtering the whole dataset for the value *k*_*t*_ of the reference sample, and uniformly selecting a positive sample with the same value. For a continuous context variable **c**_*t*_ we can use a set of time offsets *Δ* to specify the distribution. Given the time offsets, the empirical distribution $P\left({{\bf{c}}}_{t+\tau }\,{\rm{| }}\,{{\bf{c}}}_{t}\right)$ for a particular choice of $\tau \in \varDelta$ can be computed from the dataset: we build up a set $D=\{t\in \left[T\right],\tau \in \varDelta :{{\bf{c}}}_{t+\tau }-{{\bf{c}}}_{t}\}$, sample a **d** uniformly from *D* and obtain the sample that is closest to the reference sample’s context variable modified by this distance (**c** + **d**) from the dataset. It is possible to combine a continuous variable **c**_*t*_ with a categorical variable *k*_*t*_ for mixed sampling. On top of the continual sampling step above, it is ensured that both samples in the positive pair share the same value of *k*_*t*_.

It is crucial that the context samples **c** and the norm used in the algorithm match in some way; for simple context variables with predictable conditional distributions (for example, a 1D or 2D position of a moving animal, which can most probably be well described by a Gaussian conditional distribution based on the previous sample), the positive sample distribution can also be specified directly, for example, as a normal distribution centred around **c**_*t*_. An additional alternative is to use CEBRA also to preprocess the original context samples **c** and use the embedded context samples with the metric used for CEBRA training. This scheme is especially useful for higher-dimensional behavioural data, or even for complex inputs such as video.

We next consider the multi-session case in which signals ${{\bf{s}}}_{t}^{\left(i\right)}\in {{\mathbb{R}}}^{{n}_{i}}$ come from *N* different sessions $i\in \left[N\right]$ with session-dependent dimensionality *n*_*i*_. Importantly, the corresponding continuous context variables ${{\bf{c}}}_{t}^{\left(i\right)}\in {{\mathbb{R}}}^{m}$ share the same dimensionality *m*, which makes it possible to relate samples across sessions. The multi-session setup is similar to mixed-session sampling (if we treat the session ID as a categorical variable ${k}_{t}^{(i)}:\,=i$ for all time steps *t* in session *i*). The conditional distribution for both negative and positive pairs is uniformly sampled across sessions, irrespective of session length. Multi-session mixed or discrete sampling can be implemented analogously.

CEBRA is sufficiently flexible to incorporate more specialized sampling schemes beyond those outlined above. For instance, mixed single-session sampling could be extended additionally to incorporate a dimension to which the algorithm should become invariant; this would add an additional step of uniform sampling with regard to this desired discrete variable (for example, via ancestral sampling).

#### Choice of reference, positive and negative samples

Depending on the exact application, the contrastive learning step can be performed by explicitly including or excluding the context variable. The reference sample **x** can contain information from the signal **s**_*t*_, but also from the experimental conditions, behavioural recordings or other context variables. The positive and negative samples **y** are set to the signal variable **s**_*t*_.

#### Theoretical guarantees for linear identifiability of CEBRA models

Identifiability describes the property of an algorithm to give a consistent estimate for the model parameters given that the data distributions match. We here apply the relaxed notion of linear identifiability that was previously discussed and used^13,14^. After training two encoder models **f** and **f**′, the models are linearly identifiable if **f**(**x**) = **L**f(**x**), where **L** is a linear map.

When applying CEBRA, three cases are of potential interest. (1) When applying discovery-driven CEBRA, will two models estimated on comparable experimental data agree in their inferred representation? (2) Under which assumptions about the data will we be able to discover the true latent distribution? (3) In the hypothesis-driven or hybrid application of CEBRA, is the algorithm guaranteed to give a meaningful (nonstandard) latent space when we can find signal within the data?

For the first case, we note that the CEBRA objective with a cosine similarity metric follows the canonical discriminative form for which Roeder et al.^13^ showed linear identifiability: for sufficiently diverse datasets, two CEBRA models trained to convergence on the same dataset will be consistent up to linear transformations. Note that the consistency of CEBRA is independent of the exact data distribution: it is merely required that the embeddings of reference samples across multiple positive pairs, and the embeddings of negative samples across multiple negative pairs, vary in sufficiently numerous linearly independent directions. Alternatively, we can derive linear identifiability from assumptions about data distribution: if the ground truth latents are sufficiently diverse (that is, vary in all latent directions under distributions *p* and *q*), and the model is sufficiently parameterized to fit the data, we will also obtain consistency up to a linear transformation. See Supplementary Note 2 for a full formal discussion and proof.

For the second case, additional assumptions are required regarding the exact form of data-generating distributions. Within the scope of this work we consider ground truth latents distributed on the hypersphere or Euclidean space. The metric then needs to match assumptions about the variation of ground truth latents over time. In discovery-driven CEBRA, using the dot product as the similarity measure then encodes the assumption that latents vary according to a von Mises–Fisher distribution whereas the (negative) mean squared error encodes an assumption that latents vary according to a normal distribution. More broadly, if we assume that the latents have a uniform marginal distribution (which can be ensured by designing unbiased experiments), the similarity measure should be chosen as the log-likelihood of conditional distribution over time. In this case, CEBRA identifies the latents up to an affine transformation (in the most general case).

This result also explains the empirically high performance of CEBRA for decoding applications: if trained for decoding (using the variable to decode for informing the conditional distribution), it is trivial to select matching conditional distributions because both quantities are directly selected by the user. CEBRA then ‘identifies’ the context variable up to an affine transformation.

For the third case, we are interested in hypothesis-testing capabilities. We can show that if a mapping exists between the context variable and the signal space, CEBRA will recover this relationship and yield a meaningful embedding, which is also decodable. However, if such a mapping does not exist we can show that CEBRA will not learn a structured embedding.

### CEBRA models

We chose *X* = *Y* as the neural signal, with varying levels of recorded neurons and channels based on the dataset. We used three types of encoder model based on the required receptive field: a receptive field of one sample was used for the synthetic dataset experiments (Fig. 1b) and a receptive field of ten samples in all other experiments (rat, monkey, mouse) except for the Neuropixels dataset, in which a receptive field of 40 samples was used due to the fourfold higher sampling rate of the dataset.

All feature encoders were parameterized by the number of neurons (input dimension), a hidden dimension used to control model size and capacity, as well as by their output (embedding) dimension. For the model with the receptive field of one, a four-layer MLP was used. The first and second layers map their respective inputs to the hidden dimension whereas the third introduces a bottleneck and maps to half the hidden dimension. The final layer maps to the requested output dimension. For the model with a receptive field of ten, a convolutional network with five time-convolutional layers was used. The first layer had a kernel size of two, and the next three had a kernel size of three and used skip connections. The final layer had a kernel size of three and mapped hidden dimensions to the output dimension. For the model with receptive field 40, we first preprocessed the signal by concatenating a 2× downsampled version of the signal with a learnable downsample operation implemented as a convolutional layer with kernel size four and stride two, directly followed (without activation function between) by another convolutional layer with kernel size three and stride two. After these first layers, the signal was subsampled by a factor of four. Afterwards, similar to the receptive field ten model, we applied three layers with kernel size three and skip connections and a final layer with kernel size three. In all models, Gaussian error linear unit activation functions^54^ were applied after each layer except the last. The feature vector was normalized after the last layer unless a mean squared error-based similarity metric was used (as shown in Extended Data Fig. 8).

Our implementation of the InfoNCE criterion received a minibatch (or the full dataset) of size *n* × *d* for each of the reference, positive and negative samples. *n* dot-product similarities were computed between reference and positive samples and *n* × *n* dot-product similarities were computed between reference and negative samples. Similarities were scaled with the inverse of the temperature parameter *τ*:


from torch import einsum, logsumexp, no_grad*def info_nce(ref, pos, neg, tau = 1.0):*pos_dist = einsum(“nd,nd–>n”, ref, pos)/tauneg_dist = einsum(“nd,md–>nm”, ref, neg)/tauwith no_grad():c, _ = neg_dist.max(dim=1)pos_dist = pos_dist – c.detach()neg_dist = neg_dist – c.detach()pos_loss = –pos_dist.mean()neg_loss = logsumexp(neg_dist, dim = 1).mean()return pos_loss + neg_loss


Alternatively, a learnable temperature can be used. For a numerically stable implementation we store the log inverse temperature $\alpha =-\,\log \tau$ as a parameter of loss function. At each step we scale the distances in loss function with $\min \left(\exp \alpha ,\,1/{\tau }_{\min }\right)$. The additional parameter *τ*_min_ is a lower bound on the temperature. The inverse temperature used for scaling the distances in the loss will hence lie in $(0,1/{\tau }_{\min }]$.

#### CEBRA model parameters used

In the main figures we have used the default parameters (https://cebra.ai/docs/api.html) for fitting CEBRA unless otherwise stated in the text (such as dimension, which varied and is noted in figure legends), or below:

Synthetic data: model_architecture= ‘offset1-model-mse’, conditional= ‘delta’, delta=0.1, distance= ‘euclidean’, batch_size=512, learning_rate=1e-4.

Rat hippocampus neural data: model_architecture= ‘offset10-model’, time_offsets=10, batch_size=512.

Rat behavioural data: model_architecture= ‘offset10-model-mse’, distance= ‘euclidean’, time_offsets=10, batch_size=512.

Primate S1 neural data: model_architecture= ‘offset10-model’, time_offsets=10, batch_size=512.

Allen datasets (2P): model_architecture= ‘offset10-model’, time_offsets=10, batch_size=512.

Allen datasets (NP): model_architecture= ‘offset40-model-4x-subsample’, time_offsets=10, batch_size=512.

#### CEBRA API and example usage

The Python implementation of CEBRA is written in PyTorch^55^ and NumPy^56^ and provides an application programming interface (API) that is fully compatible with scikit-learn^57^, a package commonly used for machine learning. This allows the use of scikit-learn tools for hyperparameter selection and downstream processing of the embeddings—for example, decoding. CEBRA can be used as a dropin replacement in existing data pipelines for algorithms such as *t*-SNE, UMAP, PCA or FastICA. Both CPU and GPU implementations are available.

Using the previously introduced notations, suppose we have a dataset containing signals **s**_*t*_, continuous context variables **c**_*t*_ and discrete context variables *k*_*t*_ for all time steps *t*,


import numpy as npN = 500s = np.zeros((N, 55), dtype = float)k = np.zeros((N,), dtype = int)c = np.zeros((N, 10), dtype = float)


along with a second session of data,


s2 = np.zeros((N, 75), dtype = float)c2 = np.zeros((N, 10), dtype = float)


assert c2.shape[1] == c.shape[1]:note that both the number of samples and the dimension in **s**′ does not need to match **s**. Session alignment leverages the fact that the second dimensions of **c** and **c**′ match. With this dataset in place, different variants of CEBRA can be applied as follows:


import cebramodel = cebra.CEBRA(output_dimension=8,num_hidden_units=32,batch_size=1024,learning_rate=3e-4,max_iterations=1000)


The training mode to use is determined automatically based on what combination of data is passed to the algorithm:


*# time contrastive learningmodel.fit(s)# discrete behaviour contrastive learningmodel.fit(s, k)# continuous behaviour contrastive learningmodel.fit(s, c)# mixed behaviour contrastive learningmodel.fit(s, c, k)# multi-session trainingmodel.fit([s, s2], [c, c2])# adapt to new sessionmodel.fit(s, c)model.fit(s2, c2, adapt = True)*


Because CEBRA is a parametric method training a neural network internally, it is possible to embed new data points after fitting the model:


s_test = np.zeros((N, 55), dtype=float)# obtain and plot embeddingz = model.transform(s_test)plt.scatter(z[:, 0], z[:, 1])plt.show()


Besides this simple-to-use API for end users, our implementation of CEBRA is a modular software library that includes a plugin system, allowing more advanced users to readily add additional model implementations, similarity functions, datasets and data loaders and distributions for sampling positive and negative pairs.

### Consistency of embeddings across runs, subjects, sessions, recording modalities and areas

To measure the consistency of the embeddings we used the *R*^2^ score of linear regression (including an intercept) between embeddings from different subjects (or sessions). Secondly, pi-VAE, which we benchmarked and improved (Extended Data Fig. 1), demonstrated a theoretical guarantee that it can reconstruct the true latent space up to an affine transformation. Across runs, we measured the *R*^2^ score of linear regression between embeddings across ten runs of the algorithms, yielding 90 comparisons. These runs were done with the same hyperparameters, model and training setup.

For the rat hippocampus data, the numbers of neurons recorded were different across subjects. The behaviour setting was the same: the rats moved along a 1.6-meter-long track and, for analysis, behaviour data were binned into 100 bins of equal size for each direction (leftwards, rightwards). We computed averaged feature vectors for each bin by averaging all normalized CEBRA embeddings for a given bin and renormalized the average to lie on the hypersphere. If a bin did not contain any sample, it was filled by samples from the two adjacent bins. CEBRA was trained with latent dimension three (the minimum) such that it was constrained to lie only on a two-sphere (making this ‘3D’ space equivalent to a 2D Euclidean space). All other methods were trained with two latent dimensions in Euclidean space. Note that *n* + 1 dimensions of CEBRA are equivalent to *n* dimensions of other methods that we compared, because the feature space of CEBRA is normalized (that is, the feature vectors are normalized to have unit length).

For Allen visual data in which the number of behavioural data points is the same across different sessions (that is, fixed length of video stimuli), we directly computed the *R*^2^ score of linear regression between embeddings from different sessions and modalities. We surveyed three, four, eight, 32, 64 and 128 latent dimensions with CEBRA.

To compare the consistency of embeddings between or within the areas considered, we computed intra- and interarea consistency within the same recording modality (2P or NP). Within the same modality we sampled 400 neurons from each area. We trained one CEBRA model per area and computed linear consistency between all pairs of embeddings. For intra-area comparison we sampled an additional 400 disjoint neurons. For each area we trained two CEBRA models on these two sets of neurons and computed their linear consistency. We repeated this process three times.

For comparisons across modalities (2P and NP) we sampled 400 neurons from each modality (which are disjoint, as above, because one set was sampled from 2P recordings and the other from NP recordings). We trained a multi-session CEBRA model with one encoder for 2P and one for NP in the same embedding space. For intra-area comparison we computed linear consistency between NP and 2P decoders from the same area. For interarea comparison we computed linear consistency between the NP encoder from one area and the 2P encoder from another and again considered all combinations of areas. We repeated this process three times.

For comparison of single- and multi-session training (Extended Data Fig. 7) we computed embeddings using encoder models with eight, 16, *…*, 128 hidden units to vary the model size, and benchmarked eight, 16, *…*, 128 latent dimensions. Hyperparameters, except for number of optimization steps, were selected according to either validation set decoding *R*^2^ (rat) or accuracy (Allen). Consistency was reported as the point in training at which position decoding error was less than 7 cm for the first rat in the hippocampus dataset, and a decoding accuracy of 60% in the Allen dataset. For single-session training, four embeddings were trained independently on each individual animal whereas for multi-session training the embeddings were trained jointly on all sessions. For multi-session training, the same number of samples was drawn from each session to learn an embedding invariant to the session ID. The consistency versus decoding error trade-off (Extended Data Fig. 7c) was reported as the average consistency across all 12 comparisons (Extended Data Fig. 7b) versus average decoding performance across all rats and data splits.

### Model comparisons

#### pi-VAE parameter selection and modifications to pi-VAE

Because the original implementation of pi-VAE used a single time bin spiking rate as an input, we therefore modified their code to allow for larger time bin inputs and found that time window input with a receptive field of ten time bins (250 ms) gave higher consistency across subjects and better preserved the qualitative structure of the embedding (thereby outperforming the results presented by Zhou and Wei^5^; Extended Data Fig. 1). To do this we used the same encoder neural network architecture as that for CEBRA and modified the decoder to a 2D output (we call our modified version conv-pi-VAE). Note, we used this modified pi-VAE for all experiments except for the synthetic setting, for which there is no time dimension and thus the original implementation is sufficient.

The original implementation reported a median absolute error of 12 cm for rat 1 (the individual considered most in that work), and our implementation of time-windowed input with ten bins resulted in a median absolute error of 11 cm (Fig. 2). For hyperparameters we tested different epochs between 600 (the published value used) and 1,000, and learning rate between 1.0 × 10^−6^ and 5.0 × 10^−4^ via a grid search. We fixed hyperparameters as those that gave the highest consistency across subjects, which were training epochs of 1,000 and learning rate 2.5 × 10^−4^. All other hyperparameters were retained as in the original implementation^5^. Note that the original paper demonstrated that pi-VAE is fairly robust across different hyperparameters. For decoding (Fig. 2) we considered both a simple kNN decoder (that we use for CEBRA) and the computationally more expensive Monte Carlo sampling method originally proposed for pi-VAE^5^. Our implementation of conv-pi-VAE can be found at https://github.com/AdaptiveMotorControlLab/CEBRA.

#### autoLFADS parameter selection

AutoLFADS^25^ includes a hyperparameter selection and tuning protocol, which we used, and we also used the original implementation (https://github.com/snel-repo/autolfads-tf2/, https://github.com/neurallatents/nlb_tools/tree/main/examples/baselines/autolfads). For the rat hippocampus dataset we chopped the continuous spiking rate (25 ms bin size) into 250-ms-long segments with 225 ms overlap between segments to match the training setup for CEBRA, UMAP, *t*-SNE and pi-VAE. We used population-based training (PBT) for hyperparameter searches and constrained the search range to default values given in the original script (initial learning rate between 1.0 × 10^−5^ and 5.0 × 10^−3^, dropout rate 0–0.6, coordinated dropout rate 0.01–0.70, L2 generator weight between 1.0 × 10^−4^ and 1.0, L2 controller weight between 1.0 × 10^−4^ and 1.0, KL controller weight between 1.0 × 10^−6^ and 1.0 × 10^−4^ and KL initial condition weight between 1.0 × 10^−6^ and 1.0 × 10^–3^). The negative log-likelihood metric was used to select the best hyperparameters. Each generation of PBT consisted of 25 training epochs and we trained for a maximum of 5,000 epochs of batch size 100 while executing early stopping after awaiting 50 epochs. The PBT search was done using 20 parallel workers on each rat.

#### UMAP parameter selection

For UMAP^11^, following the parameter guide (umap-learn.readthedocs.io/), we focused on tuning the number of neighbours (*n*_*neighbors*) and minimum distance (*min*_*dist*). The *n*_*components* parameter was fixed to 2 and we used a cosine metric to make a fair comparison with CEBRA, which also used the cosine distance metric for learning. We performed a grid search with 100 total hyperparameter values in the range [2, 200] for *n*_*neighbors* and in the range [0.0001, 0.99] for *min*_*dist*. The highest consistency across runs in the rat hippocampus dataset was achieved with *min*_*dist* of 0.0001 and *n*_*neighbors* of 24. For the other datasets in Extended Data Fig. 3 we used the default value of *n*_*neighbors* as 15 and *min*_*dist* as 0.1.

#### *t*-SNE parameter selection

For *t*-SNE^12^ we used the implementation in openTSNE^58^. We performed a sweep on *perplexity* in the range [5, 50] and *early*_*exaggeration* in the range [12, 32] following the parameter guide, while fixing *n*_*components* as 2 and used a cosine metric for fair comparison with UMAP and CEBRA. We used PCA initialization to improve the run consistency of *t*-SNE^59^. The highest consistency across runs in the rat hippocampus dataset was achieved with *perplexity* of ten and *early*_*exaggeration* of 16.44. For the other datasets in Extended Data Fig. 3 we used the default value for *perplexity* of 30 and for *early*_*exaggeration* of 12.

### Decoding analysis

We primarily used a simple kNN algorithm, a nonparametric supervised learning method, as a decoding method for CEBRA. We used the implementation in scikit-learn^57^. We used a kNN regressor for continuous value regression and a kNN classifier for discrete label classification. For embeddings obtained with cosine metrics we used cosine distance metrics for kNN, and Euclidean distance metrics for those obtained in Euclidean space.

For the rat hippocampus data a kNN regressor, as implemented in scikit-learn^57^, was used to decode position and a kNN classifier to decode direction. The number of neighbours was searched over the range [1, 4, 9, 16, 25] and we used the cosine distance metric. We used the *R*^2^ score of predicted position and direction vector on the validation set as a metric to choose the best *n_neighbours* parameter. We report the median absolute error for positional decoding on the test set. For pi-VAE, we additionally evaluated decoding quality using the originally proposed decoding method based on Monte Carlo sampling, with the settings from the original article^5^. For autoLFADS, use of their default Ridge regression decoder^25^ performed worse than our kNN decoder, which is why we reported all results for the kNN decoder. Note that UMAP, *t*-SNE and CEBRA-Time were trained using the full dataset without label information when learning the embedding, and we used the above split only for training and cross-validation of the decoder.

For direction decoding within the monkey dataset we used a Ridge classifier^57^ as a baseline. The regularization hyperparameter was searched over [10^−6^, 10^2^]. For CEBRA we used a kNN classifier for decoding direction with *k* searched over the range [1, 2500]. For conv-pi-VAE we searched for the best learning rate over [1.0 × 10^−5^, 1.0 × 10^−3^]. For position decoding we used Lasso^57^ as a baseline. The regularization hyperparameter was searched over [10^−6^, 10^2^]. For conv-pi-VAE we used 600 epochs and searched for the best learning rates over [5 × 10^−4^, 2.5 × 10^−4^, 0.125 × 10^−4^, 5 × 10^−5^] via a grid of (*x*,*y*) space in 1 cm bins for each axis as the sampling process for decoding. For CEBRA we used kNN regression, and the number of neighbours *k* was again searched over [1, 2500].

For the Allen Institute datasets we performed decoding (frame number or scene classification) for each frame from Video 1. Here we used a kNN classifier^57^ with a population vector kNN as a baseline, similar to the decoding of orientation grating performed in ref. ^46^. For CEBRA we used the same kNN classifier method as on CEBRA features. In both cases the number of neighbours, *k*, was searched over a range [1, 100] in an exponential fashion. We used neural data recorded during the first eight repeats as the train set, the ninth repeat for validation in choosing the hyperparameter and the last repeat as the test set to report decoding accuracy. We also used a Gaussian naive Bayes decoder^57^ to test linear decoding from the CEBRA model and neural population vector. Here we assumed uniform priors over frame number and searched over the range [10^−10^, 10^3^] in an exponential manner for the *var_smoothing* hyperparameter.

For layer-specific decoding we used data from excitatory neurons in area VISp: layers 2/3 [Emx1-IRES-Cre, Slc17a7-IRES2-Cre]; layer 4 [Cux2-CreERT2, Rorb-IRES2-Cre, Scnn1a-Tg3-Cre]; and layers 5/6 [Nr5a1-Cre, Rbp4-Cre_KL100, Fezf2-CreER, Tlx3-Cre_PL56, Ntrsr1-cre].

#### Neural Latents Benchmark

We tested CEBRA on the *mc-maze* 20 ms task from the Neural Latents Benchmark^37^ (https://eval.ai/web/challenges/challenge-page/1256/leaderboard/3183). We trained the offset10-model with 48 output dimensions and [128, 256, 512] hidden units, as presented throughout the paper. We trained, in total, 48 models by additionally varying the temperature in [0.0001, 0.004] and time offsets from {1, 2}. We performed smoothing of input neural data using a Gaussian kernel with 50 ms s.d. Lastly, we took 45 embeddings from the trained models picked by the validation score, aligned the embeddings (using the Procrustes method^60^) and averaged them.

### Topological analysis

For the persistent cohomology analysis we utilized ripser.py^61^. For the hippocampus dataset we used 1,000 randomly sampled points from CEBRA-Behaviour trained with temperature 1, time offset 10 and minibatch size 512 for 10,000 training steps on the full dataset and then analysed up to 2D cohomology. Maximum distance considered for filtration was set to infinity. To determine the number of cocycles in each cohomology dimension with a significant lifespan we trained 500 CEBRA embeddings with shuffled labels, similar to the approach taken in ref. ^33^. We took the maximum lifespan of each dimension across these 500 runs as a threshold to determine robust Betti numbers, then surveyed the Betti numbers of CEBRA embeddings across three, eight, 16, 32 and 64 latent dimensions.

Next we used DREiMac^62^ to obtain topology-preserving circular coordinates (radial angle) of the first cocycle (H^1^) from the persistent cohomology analysis. Similar to above, we used 1,000 randomly sampled points from the CEBRA-Behaviour models of embedding dimensions 3, 8, 16, 32 and 64.

### Behaviour embeddings for video datasets

High-dimensional inputs, such as videos, need further preprocessing for effective use with CEBRA. First we used the recently presented DINO model^48^ to embed video frames into a 768D feature space. Specifically we used the pretrained ViT/8 vision transformer model, which was trained by a self-supervised learning objective on the ImageNet database. This model is particularly well suited for video analysis and among the state-of-the-art models available for embedding natural images into a space appropriate for a kNN search^48^, a desired property when making the dataset compatible with CEBRA. We obtained a normalized feature vector for each video frame, which was then used as the continuous behaviour variable for all further CEBRA experiments.

For scene labels, three individuals labelled each video frame using eight candidate descriptive labels allowing multilabel classes. We took the majority vote of these three individuals to determine the label of each frame. In the case of multilabels we considered this as a new class label. The above procedure resulted in ten classes of frame annotation.

### Reporting summary

Further information on research design is available in the Nature Portfolio Reporting Summary linked to this article.

## Online content

Any methods, additional references, Nature Portfolio reporting summaries, source data, extended data, supplementary information, acknowledgements, peer review information; details of author contributions and competing interests; and statements of data and code availability are available at 10.1038/s41586-023-06031-6.

## Supplementary information

Supplementary Information Attached in a single PDF are Supplementary Notes 1 and 2, which provide extended discussions on identifiability and the theoretical guarantees of CEBRA, respectively, and Tables 1–6, which provide statistical support to the conclusions drawn in the main manuscript.

Reporting Summary

Supplementary Video 1 Corresponding to Fig. 2d. CEBRA-Behaviour trained with position and direction on rat 1. Video is in 2× real time.

Supplementary Video 2 Corresponding to Fig. 5b. The left-hand panels show example calcium traces from two-photon imaging (top) and spikes from Neuropixels recording (bottom) of primary visual cortex while the video was shown to mice (here, we randomly picked neurons to visualize the pseudomouse). The centre panel shows an embedding space constructed by jointly training a CEBRA-Behaviour model with two-photon and Neuropixels recordings using DINO frame features as labels. The trace is an embedding of a held-out test repeat from the Neuropixels recording. The colour map indicates the frame number of the 30-s-long video (30 Hz). The final panels show true video (top) and predicted frame sequence (bottom) using a kNN decoder on CEBRA-Behaviour embedding from the test set. Video is in real time.

## Data Availability

Hippocampus dataset: https://crcns.org/data-sets/hc/hc-11/about-hc-11, and we used the preprocessing script from https://github.com/zhd96/pi-vae/blob/main/code/rat_preprocess_data.py. Primate dataset: https://gui.dandiarchive.org/#/dandiset/000127. Allen Institute dataset: Neuropixels data are available at https://allensdk.readthedocs.io/en/latest/visual_coding_neuropixels.html. The preprocessed 2P recordings are available at https://github.com/zivlab/visual_drift/tree/main/data.

## Code availability

Code: https://github.com/AdaptiveMotorControlLab/CEBRA. Code used to reproduce the figures: https://github.com/AdaptiveMotorControlLab/CEBRA-figures.
