# Supplementary material for: Learnable latent embeddings for joint behavioural and neural analysis
Source: Nature. 2023 May 3;617(7960):360–8. doi: 10.1038/s41586-023-06031-6 (PMC10172131; doi:10.1038/s41586-023-06031-6)
Supplement: Supplementary file 1 — Attached in a single PDF are Supplementary Notes 1 and 2, which provide extended discussions on identifiability and the theoretical guarantees of CEBRA, respectively, and Tables 1–6, which provide statistical support to the conclusions drawn in the main manuscript. [file 41586_2023_6031_MOESM1_ESM.pdf]

nature portfolio

https://doi.org/10.1038/s41586-023-06031-6

Supplementary information

Learnable latent embeddings for joint behavioural and neural analysis

In the format provided by the authors and unedited

Supplementary Information

Supplementary Note 1

On identifiability and consistency

When learning (non-linear) representations of a dataset, it is highly desirable that embedding algorithms generate *consistent* embedding spaces. Multiple runs of the algorithm on the same data, multiple runs of the algorithm on data produced in the same way, etc., should generate embedding spaces with a meaningful relation to each other. This "meaningful relation" between algorithm runs can be formalized using tools from identifiability in non-linear independent component analysis (ICA). Suppose we are given two models $\mathbf{f}'$ and $\mathbf{f}^*$ trained on the same dataset, and the performance of these models matches in the sense that they represent the same probability distribution $p' = p^*$. Identifiability then entails that both models are the same up to some known class of transformations (e.g., linear or affine transformations, rotations, permutations and sign-flips, etc.).

For example, one option for parameterizing the distributions is as $p'(\mathbf{y}|\mathbf{x}, \mathbf{y}_1 \ldots \mathbf{y}_n) = \exp(\mathbf{f}(\mathbf{x})^\top \mathbf{f}'(\mathbf{y})) / \sum_i \exp(\mathbf{f}(\mathbf{x})^\top \mathbf{f}'(\mathbf{y}_i))$ and respectively for $\tilde{p}'$ defined respectively with $\tilde{\mathbf{f}}$ and $\tilde{\mathbf{f}}'$. Roeder et al. [9] show that if the two distributions match contrastive learning models produce consistent embedding spaces, and it is possible to find a linear mapping $\mathbf{L}$ between the feature spaces, i.e., $\mathbf{L}\mathbf{f}(\mathbf{x}) = \tilde{\mathbf{f}}(\mathbf{x})$ for all $\mathbf{x}$ in the dataset. Other theoretical work has shown that contrastive learning with auxiliary variables is identifiable for bijective neural networks using the noise contrastive estimation (NCE) loss [2], and that with an InfoNCE loss this bijectivity assumption can be removed for certain distributions [13]. We will adapt the underlying proofs to our setup in Suppl. Note 2, and give a high-level outline below.

We will consider two important points in both the context of discovery and hypothesis driven training of CEBRA models. Firstly, when applying discovery-driven CEBRA, will two models estimated on comparable experimental data agree in their inferred representation? Second, under which assumptions about the data will we be able able to discover the *true* latent distribution?

Consistency: For consistency across embedding spaces, we require a dataset with a sufficient amount of variability in time. Intuitively, to estimate a d dimensional embedding that is consistent across runs, points sampled from the embedding via the negative distribution q need to vary in at least d directions for each possible reference sample in the dataset. Interestingly, consistency is mostly independent from the data generating process (i.e., the data modality of the recording) and merely requires a sufficiently varying dataset, as well as a choice of feature encoder that passes this variability on to the embedding space.

For example, consider the reaching dataset in Fig. 3 where we showed embeddings that vary in two dimensions (the direction and distance from the center). In this case, the sampling process needs to be designed such that for each reference point we can draw from the dataset, the embedding of the negative samples will vary in at least two directions. This is clearly the case for our training setup: The neurons encode both position and direction information, this information is transformed by the feature encoder, and the resulting embedding varies in at least two directions when the negative distribution samples uniformly across the dataset.

For the first property, we can leverage previous results on the consistency of contrastive learning models over multiple runs [9]. Consider the case where we train multiple CEBRA models on data originating from the same data distribution, and consider that we can train these models to full convergence. It is then guaranteed that the embedding spaces will agree up to a linear indeterminacy. In other words, it will always be possible to transform one embedding space into the other by applying a linear transformation. Linear consistency of representations is interesting when we consider linear downstream processing of the inferred embedding space, as is common in neuroscience [10]. Such a downstream algorithm (e.g., a linear regression or general linear model) will yield the same performance across different CEBRA models.

Recovering the ground-truth latents: Note that this notion of consistency only makes a statement about the *inferred* latent representation (and identifiability) across multiple runs of the algorithm, but not yet about the relation between this latent representation and the *true* underlying latent variables that generated the data. This is the second property mentioned above, and requires additional assumptions about the data generating process to resolve the ambiguity of what a "latent" underlying a given dataset actually entails. The assumptions concern the injectivity of the data generating process and the positive distribution p. While for discovery driven training, p is an empirical property of the dataset, hypothesis driven training allows to precisely define p based on the observed auxiliary variables. The same theory applies to both cases. Importantly, as for consistency, note that these results are independent from the actual modality of the data and other properties of the signal space we consider. The assumptions are all with respect to the underlying latent distribution.

For the analyses in this paper, the theory for linear identifiability of the underlying latents applies: For time-contrastive training, it is required that the underlying latent distribution is uniform (e.g., there is no inherent bias in the experimental data), and that latents of nearby time steps vary according to a distribution of the form $p(\mathbf{v}|\mathbf{u}) = \exp(\phi(\mathbf{u}, \mathbf{v}))$, where $\mathbf{u}$ and $\mathbf{v}$ are the latents underlying the signal variables $\mathbf{x}$ and $\mathbf{y}$. If these conditions are met, the true underlying latents are recovered up to a linear transformation. For hypothesis testing where the user actually specifies the distribution p, this requirement can be easily validated and met.

Relationship between consistency, identifiability, and the sampling mechanism: The CEBRA software package allows for other choices of similarity measures (potentially learnable), which allows to derive guarantees also for these cases. In the most general case, we pick ϕ as a trainable neural network that factorizes into individual components, $\phi(\mathbf{x}, \mathbf{y}) := \sum_i \phi_i(y_i, \mathbf{x})$ where each ϕ_i is an individually trained neural network. For sufficiently variable distributions, this allows to recover the underlying latents up to permutations and point-wise non-linear, bijective transformations.

Likewise, it is possible to modify the encoding networks $\mathbf{f}$ and $\mathbf{f}'$ for $\mathbf{x}$ and $\mathbf{y}$, respectively. While our experiments used one network $\mathbf{f} = \mathbf{f}'$ with $\mathbf{x}$ and $\mathbf{y}$ representing neural data, it is well possible to encode different aspects of the dataset and/or to break the symmetry between the two encoding networks and to train a separate network for each of $\mathbf{f}$ and $\mathbf{f}'$. For example, neural data $\mathbf{y}$ could be encoded using $\mathbf{f}'$, and behavior $\mathbf{x}$ could be encoded using $\mathbf{f}$. It would also be possible to use a composition of neural and behavioral data for $\mathbf{x}$. In these cases, if $\mathbf{f}$ and $\mathbf{f}'$ are parameterized as two individual neural networks and ϕ is defined as the dot-product as before, if $\mathbf{y}|\mathbf{x}$ follows a conditionally exponential distribution, we are able to recover all sufficient statistics of this distribution up to a linear transformation.

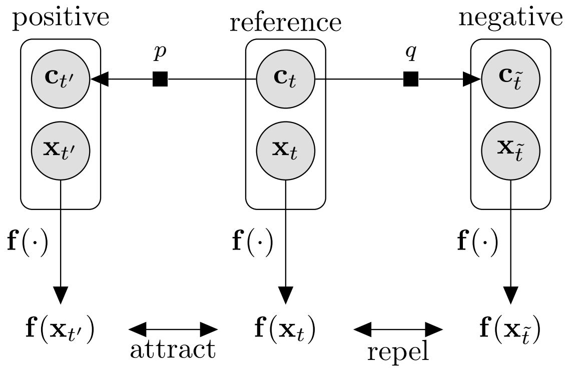

Figure 1: The contrastive learning data sampling scheme used in CEBRA.

Examples: As an example of the aforementioned results, let us consider the rat hippocampus dataset used in Fig. 1 and 2. The auxiliary information in this dataset is the position, velocity, and direction of the rat on the linear track.

We can apply different sampling schemes for investigating this dataset. For example, we can apply discovery-driven, time-contrastive learning. In this setup, we sample time steps uniformly from the dataset to arrive at our reference samples. Given a time offset Δ (that informs the algorithm about the time-scale of interest), we obtain positive samples. The resulting batch will be composed of samples $\mathbf{s}_t$ for the reference, $\mathbf{s}(t + \Delta)$ for the positive, and $\mathbf{s}(t_i)$ with uniformly sampled time steps $t_1, \ldots, t_n$ for the negative samples. This corresponds to an approximation of the distribution $p(\mathbf{u}_{t+\Delta}|\mathbf{u}_t)$ of how the latents vary over the course of time. If sufficient variation is present in the dataset along d latent directions, CEBRA models will become, after training, consistent across runs. If additionally the *true* distribution $p(\mathbf{u}_{t+\Delta}|\mathbf{u}_t)$ follows, e.g., a Gaussian distribution, CEBRA will identify the ground truth latents.

In comparison, our so-called hypothesis-driven, behavior-label guided contrastive learning approach would leverage the continuous position information as well as the movement direction of the rat. In the Methods, we denoted the continuous variable as $\mathbf{c}_t$ and the discrete variables as k_t. To arrive at a behavior contrastive embedding using this auxiliary information, we would build a set of differences. If the variables are independent (or should reflect this in the embedding), we build one set $D = \{\mathbf{c}_{t+\Delta} - \mathbf{c}_t\}_{t=1}^{T}$. For a reference sample at time step t, we sample $\mathbf{d} \sim D$ uniformly, and apply this difference to the position $\mathbf{c}_t$ at step t. We then pick the point closest to $\mathbf{c}_t + \mathbf{d}$ *and* matching the discrete variable k_t as the positive sample.

A lot of variations of this sampling process are possible to embed desirable properties and test hypotheses about the dataset. For instance, consider the primate reaching dataset: Here, $\mathbf{c}_t$ could be selected as the x/y position in space, and the discrete label k_t could denote the reaching direction. However, the 2D differences $\mathbf{c}_{t+\Delta} - \mathbf{c}_t$ will depend on k_t: A reaching direction towards the left will have most variance in negative x-direction $[-1, 0]^\top$, a reaching direction towards the top will have most variance in positive y-direction $[0, 1]^\top$. One way to work around this issue is to consider a polar representation of the position and direction and apply the scheme outlined above. Another alternative is to build the set

of differences conditional on the direction k_t, i.e., $D(k) = \{\mathbf{c}_{t+\Delta} - \mathbf{c}_t\}_{t:k_t=k}$. The sampling process is almost analogous to the rat hippocampus example above: We would sample a time step t, look up the discrete variable k_t, but then only sample from $D(k_t)$ to reflect the conditioning on the direction.

Finally, e.g., for very complex movements, it is simple to adapt additional pre-processing schemes. These could involve other deep learning algorithms like DINO used for pre-processing video data in the Allen dataset (to convert pixel data without a meaningful metric into an embedding space with desirable distance properties); they could also involve simpler processing, such as computing the principal component analysis of a higher dimensional dataset, and using the behavior data in this space.

Many other variations are possible. While the most common use cases are reflected in the CEBRA software toolbox and high-level API and readily usable, more customized use cases can be easily added by the user thanks to a straightforward extension mechanism.

Improving pi-VAE

Zhou et al [12] demonstrate that pi-VAE outperforms LFADS [7], demixed-PCA [4], UMAP [5], PCA, and pfLDS [1] using the rat and/or primate datasets (Extended Data Fig. 1). We improved the performance of pi-VAE by modifying the encoder, which allows for longer time inputs (Extended Data Fig. 1), and this improved version is used throughout, unless noted.

Comparison to autoLFADS

The overall goal of CEBRA and LFADS is different. In LFADS/autoLFADS [7, 3] the primary goal is to build high performance spiking rate predictions and downstream decoding, which are excellent tasks for auto-encoders. It is important to note that LFADS assumes a data generating process that is Poisson, as does pi-VAE [12], and requires trial-data. A major assumption in LFADS is that from the derived factors there is a linear projection to the neural rate predictions. In contrast, as we show, CEBRA can be used on many data types, from spiking neural data, calcium imaging, or behavior-only (such as from pose estimation tools, see Fig. 2b), and CEBRA does not require trials. Another consideration is that one cannot use theoretically-motivated goodness of fit metrics for finding latent embedding spaces that best fit the data. While this issue is partly addressed during parameter selection in autoLFADS, it also requires a substantial computational overhead for hyperparameter tuning. Thus, while LFADS is excellent at several spike-prediction tasks [8], it is not fully suitable for the range of settings that can be used with CEBRA.

Utilizing CEBRA across contexts

Within our framework, we assume that independent latent variables are combined by a non-linear bijective mixing function to produce neural activity. The latent variables are assumed to change over time, or be correlated to the observed auxiliary variables used to train CEBRA. No additional special structure, or implicit generative models during training are needed.

CEBRA allows for minimizing the impact of selected features on the embedding, while testing the role of others. For example, suppose you have neural data from four different animals, each from the hippocampus while the animal navigated a linear track. You hypothesize that the hippocampus encodes a continuous mapping of space along the track. In this scenario the animal ID is not important, but the spatial location of the animal is. Here, the user can specify to obtain an embedding that is invariant to the animal ID, but should incorporate the position information. Another amendable scenario is a hypothesis-free, discovery-driven approach (akin to unsupervised clustering). Here too, CEBRA can be used, with only time as the input (Fig. 1). Collectively, CEBRA can be used for both visualization of data and latent-space based embedding of neural activity for downstream tasks like decoding.

The flexibility in choosing different auxiliary variables during data analysis allows users to leverage the same algorithm for a variety of applications on a given dataset: Discovery-driven analysis by purely self-supervised learning with time-contrastive learning, hypothesis-driven analysis by comparing embedding quality derived from different behavioral variables, or replacing supervised decoding algorithms, e.g., in brain-machine-interface contexts.

Supplementary Note 2

Here we provide theoretical results for consistency and identifiability of models trained within the CEBRA framework. We proceed by showing properties of the InfoNCE loss (Prop. 1), and use them as the basis for showing that encoders trained on this loss function will become bijective under mild assumptions (Prop. 2). We then revisit existing theory on

contrastive learning, and show that CEBRA falls into a category of models for which we can obtain theoretical guarantees on both consistency (Prop 3) across different model runs and identifiability of the ground truth latent distribution for both the discovery-driven (time-contrastive) learning mode (Prop. 6) and the hypothesis-driven mode (Prop. 7). Our results leverage theory by Hyvärinen et al. [2], Wang and Isola [11], Roeder et al. [9], Zimmermann et al. [13].

It should be noted that while consistency results between model runs do not require strong assumptions about the underlying data generating process, understanding the relation between the embedding space given by CEBRA and the underlying *ground truth data generating process* naturally requires such assumptions. However, compared to assumptions in generative models (e.g., VAEs), these assumptions concern the relationship between the ground-truth latent variables, rather than making statements about the signal space.

Notation, data generation, and learning algorithm

We will use the notation presented in the Methods Section. We additionally introduce the latents $\mathbf{u}$ and $\mathbf{v}$ underlying the samples $\mathbf{x}$ and $\mathbf{y}$. We will interchangeably use the distributions $p_\mathcal{D}$, p and q for either the latents $\mathbf{u}$ and $\mathbf{v}$ or their respective samples $\mathbf{x}$ and $\mathbf{y}$ depending on their arguments (we will show after Proposition 1 that this treatment is also formally correct due to the training setup considered here). Definitions, propositions and theorems adapted from other works are cited and denoted by upper-case letters and are otherwise adapted to our notation.

Definition 1 (Data generating process and encoder)**.** *Let $\mathbf{u} \in \mathbb{R}^d, \mathbf{v} \in \mathbb{R}^{d'}$ denote latents corresponding to the samples $\mathbf{x} \in \mathbb{R}^D$ and $\mathbf{y} \in \mathbb{R}^{D'}$ in the respective signal space which are generated according to two differentiable and injective mixing functions $\mathbf{g} : \mathbb{R}^d \mapsto \mathbb{R}^D$ and $\mathbf{g}' : \mathbb{R}^{d'} \mapsto \mathbb{R}^{D'}$,*

$$\mathbf{x} = \mathbf{g}(\mathbf{u}), \quad \mathbf{y} = \mathbf{g}'(\mathbf{v}) \tag{1}$$

and there exist optimal differentiable encoders $\mathbf{f} : \mathbb{R}^D \mapsto \mathbb{R}^E$ and $\mathbf{f}' : \mathbb{R}^{D'} \mapsto \mathbb{R}^E$ such that

$$f(\mathbf{g}(\mathbf{u}))_i = u_i \quad f'(\mathbf{g}'(\mathbf{v}))_j = v_j. \tag{2}$$

We will refer to the composition of the data generators and the encoders as $\mathbf{h} = \mathbf{f} \circ \mathbf{g}$ and $\mathbf{h}' = \mathbf{f}' \circ \mathbf{g}'$. In setups where two models are trained on potentially different mixing functions, we denote the second data generator, feature encoder, and the composition of both as $\tilde{\mathbf{h}} = \tilde{\mathbf{f}} \circ \tilde{\mathbf{g}}$ and $\tilde{\mathbf{h}}' = \tilde{\mathbf{f}}' \circ \tilde{\mathbf{g}}'$.

We consider a marginal distribution $p_\mathcal{D}(\cdot)$, the positive sample conditional distribution $p(\cdot|\cdot)$ and the negative conditional distribution $q(\cdot|\cdot)$. Reference samples $\mathbf{u}$ from the (true) latent space are mapped to signal space by the injective function $\mathbf{g}$, positive/negative samples $\mathbf{v}$ from the (potentially different latent space) are mapped to (a possibly different) signal space $\mathbf{y}$ by the injective function $\mathbf{g}'$. The encoder $\mathbf{f}$ is applied to $\mathbf{x}$ and the encoder $\mathbf{f}'$ is applied to $\mathbf{y}$ to recover the respective latents underlying $\mathbf{x}$ and $\mathbf{y}$. The similarity measure is denoted as ϕ with $\mathbf{f}(\mathbf{x})$ and $\mathbf{f}'(\mathbf{y})$ as its arguments. Note that ϕ does not need to be a fixed function and can also be parameterized by a learnable neural network. As in the Methods, we denote $\psi(\mathbf{x}, \mathbf{y}) = \phi(\mathbf{f}(\mathbf{x}), \mathbf{f}'(\mathbf{y}))$ and additionally introduce $\psi(\mathbf{u}, \mathbf{v}) := \phi(\mathbf{h}(\mathbf{u}), \mathbf{h}'(\mathbf{v}))$ without additional subscripts, as the desired shortcut will be clear from the context and its arguments.

Note that this is a very general setup. We typically would assume that the number of dimensions in the (shared) latent space is the same, $d = d'$, and could further assume that the number of dimensions E of the embedding space is also matching.

We recall the contrastive objective in the limit of unlimited samples from the main text:

Definition 2 (Generalized InfoNCE objective)**.** *In the limit of unlimited negative samples, the InfoNCE objective is a functional*

$$\mathcal{L}[\psi]_{\text{asympt}} = \int p_\mathcal{D}(\mathbf{x}) \left[\log \int q(\mathbf{y}|\mathbf{x}) e^{\psi(\mathbf{x},\mathbf{y})} d\mathbf{y} - \int p(\mathbf{y}|\mathbf{x}) \psi(\mathbf{x}, \mathbf{y}) d\mathbf{y} \right] d\mathbf{x}, \tag{3}$$

depending on the positive sample conditional density $p(\mathbf{y}|\mathbf{x})$, the negative sample density $q(\mathbf{y}|\mathbf{x})$, the marginal density $p_\mathcal{D}(\mathbf{x})$ and the embedding similarity ψ as defined above.

We call this objective "generalized" as it extends the original definition of the InfoNCE loss used in the literature. Oord et al. [6] introduced an objective where the marginal (there: prior) $p_\mathcal{D}$ and the negative sample distribution q matched, which influences the types of functions that can be learned. The discussion of the objective by Wang and Isola [11] makes stronger assumptions about the nature of the conditional distribution p for the positive pair, and only considers uniform choices for the marginal $p_\mathcal{D}$ and negative conditional q.

Overall, in CEBRA, the key difference to prior uses of the InfoNCE objective is the ability to control the properties of the embedding space through p and q and ϕ, and leverage this to retrieve the discovery-driven, hypothesis-driven, and hybrid

modes as demonstrated in the main text. In fact, for hypothesis-driven training, the distributions used for sampling do not even need to be connected to the underlying data generating process—instead, varying p and testing to enforce various neighbourhood relations on the neural data is used as a tool to discover meaningful relations between the auxiliary variables (e.g., behavior) and signal (e.g., neural activity). In the same manner, p and q can be selected such that a particular factor is sampled uniformly, to enforce invariance (e.g. across a subject, or a modality variable).

The loss optimized in practice acts on a limited number of negative samples in each mini-batch:

Definition 3 (Generalized InfoNCE objective with limited batch size)**.** *For a fixed number of negative samples n, the InfoNCE objective is the functional*

$$\mathcal{L}[\psi]_n = \mathop{\mathbb{E}}_{\substack{\mathbf{x}\sim p_{\mathcal{D}}(\mathbf{x}),\ \mathbf{y}_+\sim p(\mathbf{y}|\mathbf{x}) \\ \mathbf{y}_1,\ldots,\mathbf{y}_n\sim q(\mathbf{y}|\mathbf{x})}} \left[-\psi(\mathbf{x}, \mathbf{y}_+) + \log \sum_{i=1}^{n} e^{\psi(\mathbf{x},\mathbf{y}_i)} \right],$$

depending on the positive sample conditional density $p(\mathbf{y}|\mathbf{x})$, the negative sample density $q(\mathbf{y}|\mathbf{x})$, the marginal density $p_{\mathcal{D}}(\mathbf{x})$ and the embedding similarity ψ as defined above.

Both losses can be related due to Theorem 1 by Wang and Isola [11]. In the limit of unlimited samples $n \to \infty$, we obtain for the batch size n:

$$\mathcal{L}[\psi]_{\text{asympt}} = \lim_{n\to\infty} \left(\mathcal{L}[\psi]_n - \log n \right). \tag{4}$$

For a sufficiently large batch size, we can leverage the quantity $\mathcal{L}[\psi]_n - \log n$ as a goodness of fit measure (as outlined in the Methods) that estimates the distance from a "default" embedding. When comparing models with equal batch size n, note that the InfoNCE loss can also directly serve as this metric.

Minimizers of the generalized InfoNCE loss

In this section, we show that optimizing the generalized InfoNCE objective from Def. 2 yields the unique minimizer $\psi(\mathbf{x}, \mathbf{y}) = C(\mathbf{x}) + \log p(\mathbf{y}|\mathbf{x})/q(\mathbf{y}|\mathbf{x})$ or equivalently $\psi(\mathbf{u}, \mathbf{v}) = C'(\mathbf{u}) + \log p(\mathbf{v}|\mathbf{u})/q(\mathbf{v}|\mathbf{u})$ up to an arbitrary constant function $C'(\mathbf{u})$ than can depend on the latents of the reference latents. In this regard, the InfoNCE loss is more flexible than the standard noise constrastive estimation (NCE) loss which has a similar minimizer, but is limited to $C(\mathbf{x}) = C'(\mathbf{u}) = 0$. The minimum loss value is the negative Kullbach-Leibler divergence between the positive and negative distributions. To obtain non-trivial solutions, it is therefore important that p and q differ in a non-trivial way (which is the case for both time-contrastive and behavior-contrastive sampling outlined in the context of CEBRA).

Proposition 1. *Let $p(\cdot|\cdot)$ be the conditional distribution of the positive samples, $q(\cdot|\cdot)$ the conditional distribution of the negative samples and $p_{\mathcal{D}}(\cdot)$ the marginal distribution of the reference samples. The generalized InfoNCE objective (Def. 2) is convex in ψ with the unique minimizer*

$$\psi^*(\mathbf{x}, \mathbf{y}) = \log \frac{p(\mathbf{y}|\mathbf{x})}{q(\mathbf{y}|\mathbf{x})} + C(\mathbf{x}), \quad \textit{with} \quad \mathcal{L}[\psi^*]_{\text{asympt}} = -\mathcal{D}_{\text{KL}}(p(\cdot|\cdot)\|q(\cdot|\cdot)) \tag{5}$$

on the support of $p_{\mathcal{D}}$, where $C : \mathbb{R}^d \to \mathbb{R}$ is an arbitrary mapping.

Proof. We rewrite the objective as

$$\mathcal{L}[\psi]_{\text{asympt}} = \int p_{\mathcal{D}}(\mathbf{x}) \left[\log \int q(\mathbf{y}|\mathbf{x}) e^{\psi(\mathbf{x},\mathbf{y})} d\mathbf{y} - \int p(\mathbf{y}|\mathbf{x}) \psi(\mathbf{x}, \mathbf{y}) d\mathbf{y} \right] d\mathbf{x}, \tag{6}$$

and we can compute the first-order functional derivative (using the method discussed in Cahill 2014[1])

$$\begin{aligned} \delta\mathcal{L}[\psi][h]_{\text{asympt}} &= \frac{d}{d\epsilon} \mathcal{L}[\psi + \epsilon h] \big|_{\epsilon=0} \\ &= \int p_{\mathcal{D}}(\mathbf{x}) \left[\frac{1}{Z_\psi(\mathbf{x})} \int q(\mathbf{y}|\mathbf{x}) e^{\psi(\mathbf{x},\mathbf{y})} h(\mathbf{x}, \mathbf{y}) d\mathbf{y} - \int p(\mathbf{y}|\mathbf{x}) h(\mathbf{x}, \mathbf{y}) d\mathbf{y} \right] d\mathbf{x}, \\ &\text{with } Z_\psi(\mathbf{x}) = \int q(\mathbf{y}'|\mathbf{x}) e^{\psi(\mathbf{x},\mathbf{y}')} d\mathbf{y}'. \end{aligned} \tag{7}$$

The first-order functional derivative vanishes for all functions $h(\mathbf{x}, \mathbf{y})$ whenever

$$p_{\mathcal{D}}(\mathbf{x}) \left[\frac{1}{Z_\psi(\mathbf{x})} q(\mathbf{y}|\mathbf{x}) e^{\psi(\mathbf{x},\mathbf{y})} - p(\mathbf{y}|\mathbf{x}) \right] = 0. \tag{8}$$

[1] http://quantum.phys.unm.edu/523-14/ch15.pdf

This is the case iff at any point $(\mathbf{x}, \mathbf{y})$, either $p_{\mathcal{D}}(\mathbf{x}) = 0$ or

$$Z_\psi(\mathbf{x}) = \frac{q(\mathbf{y}|\mathbf{x})}{p(\mathbf{y}|\mathbf{x})} e^{\psi(\mathbf{x},\mathbf{y})}. \tag{9}$$

Since the left hand side of Eq. (9) is independent of $\mathbf{y}$, the right hand side must be independent of $\mathbf{y}$ as well. Hence all functions $\psi^*(\mathbf{x}, \mathbf{y})$ which are solutions to Eq. (9) are of the form

$$\psi^*(\mathbf{x}, \mathbf{y}) = \log \frac{p(\mathbf{y}|\mathbf{x})}{q(\mathbf{y}|\mathbf{x})} + C(\mathbf{x}), \tag{10}$$

where C is an arbitrary function depending only on $\mathbf{x}$ and not on $\mathbf{y}$. Then by definition of $Z_\psi(\mathbf{x})$,

$$Z_\psi(\mathbf{x}) = \int q(\mathbf{y}'|\mathbf{x}) e^{\psi(\mathbf{x},\mathbf{y}')} d\mathbf{y}' = e^{C(\mathbf{x})} \tag{11}$$

which is consistent when inserted into Eq. (9). Therefore the minimizers of $\mathcal{L}[\psi]_{\text{asympt}}$ form a convex connected set $\mathcal{M}$,

$$\mathcal{M} = \left\{ \psi^* : \psi^*(\mathbf{x}, \mathbf{y}) = \begin{cases} \log \frac{p(\mathbf{y}|\mathbf{x})}{q(\mathbf{y}|\mathbf{x})} + C(\mathbf{x}) & \text{if } p_{\mathcal{D}}(\mathbf{x}) \neq 0 \\ f(\mathbf{x}, \mathbf{y}) & \text{if } p_{\mathcal{D}}(\mathbf{x}) = 0 \end{cases} \right\}, \tag{12}$$

where C, f are arbitrary functions. It can be checked that all minima achieve the same value $L[\psi^*]$ given by

$$\mathcal{L}[\psi^*]_{\text{asympt}} = -\int p_{\mathcal{D}}(\mathbf{x}) \int p(\mathbf{y}|\mathbf{x}) \log \frac{p(\mathbf{y}|\mathbf{x})}{q(\mathbf{y}|\mathbf{x})} d\mathbf{y} = -\int p_{\mathcal{D}}(\mathbf{x}) D_{\text{KL}} \left[p(\cdot|\mathbf{x}) || q(\cdot|\mathbf{x})\right] d\mathbf{x} \leq 0. \tag{13}$$

It is left to show that the objective function is convex. The second-order functional derivative is given by

$$\begin{aligned}
\delta^2 \mathcal{L}[\psi][h]_{\text{asympt}} &= \frac{d^2}{d\epsilon^2} \mathcal{L}[\psi + \epsilon h]\big|_{\epsilon=0} \\
&= \frac{d}{d\epsilon} \int p(\mathbf{x}) \left[\frac{1}{Z_{\psi+\epsilon h}(\mathbf{x})} \int q(\mathbf{y}|\mathbf{x}) e^{\psi(\mathbf{x},\mathbf{y}) + \epsilon h(\mathbf{x},\mathbf{y})} h(\mathbf{x}, \mathbf{y}) d\mathbf{y} - \int p(\mathbf{y}|\mathbf{x}) h(\mathbf{x}, \mathbf{y}) d\mathbf{y} \right] \Bigg|_{\epsilon=0} \\
&= \int p(\mathbf{x}) \left[\mathbb{E}_{g(\mathbf{y}|\mathbf{x})} \left[h(\mathbf{x}, \mathbf{y})^2\right] - \mathbb{E}_{g(\mathbf{y}|\mathbf{x})} \left[h(\mathbf{x}, \mathbf{y})\right]^2 \right], \\
&\text{with } g(\mathbf{y}|\mathbf{x}) = \frac{1}{Z_\psi(\mathbf{x})} q(\mathbf{y}|\mathbf{x}) e^{\psi(\mathbf{x},\mathbf{y})}, \text{ and } \int g(\mathbf{y}|\mathbf{x}) d\mathbf{y} = 1.
\end{aligned} \tag{14}$$

Since $g(\mathbf{y}|\mathbf{x})$ is a probability density function, we can apply Jensen's inequality to the convex function $A \to A^2$ for the random variable $A := h(\mathbf{x}, \mathbf{y})$ to obtain

$$\mathbb{E}_{g(\mathbf{y}|\mathbf{x})} \left[h(\mathbf{x}, \mathbf{y})^2\right] - \left(\mathbb{E}_{g(\mathbf{y}|\mathbf{x})} \left[h(\mathbf{x}, \mathbf{y})\right]\right)^2 \geq 0 \quad \Rightarrow \delta^2 \mathcal{L}[\psi][h]_{\text{asympt}} = \int p(\mathbf{x}) \left[\mathbb{E}_{g(\mathbf{y}|\mathbf{x})} \left[h(\mathbf{x}, \mathbf{y})^2\right] - \left(\mathbb{E}_{g(\mathbf{y}|\mathbf{x})} \left[h(\mathbf{x}, \mathbf{y})\right]\right)^2 \right] \geq 0. \tag{15}$$

and it follows that the InfoNCE loss is convex in ψ.

To prove uniqueness of the minimum: Note that since the mapping $A \to A^2$ is not affine, a necessary and sufficient condition for equality is A to be constant, which holds iff $h(\mathbf{x}, \mathbf{y}) = h(\mathbf{x}, \mathbf{y}') := h(\mathbf{x})$ for all $\mathbf{y}$. The objective is hence strictly convex for all variations involving a variation in $\mathbf{y}$, and the second derivative vanishes for variations that only depend on $\mathbf{x}$. Variations that only depend on $\mathbf{x}$ are represented by the function C which appeared in the set of minimizers $\mathcal{M}$. □

Note that the difference between the minimizer of the NCE loss and the InfoNCE loss is the additional constant function C depending on the reference sample, which makes the loss function more flexible. Another common formulation of the InfoNCE minimizer in the literature is given as $\psi(\mathbf{x}, \mathbf{y}) = \log p(\mathbf{x}, \mathbf{y})/(p_{\mathcal{D}}(\mathbf{x}) p_{\mathcal{D}}(\mathbf{y}))$ which is a special case of our more general solution if $C(\mathbf{x}) = -\log p_{\mathcal{D}}(\mathbf{x})$, the negative distribution is chosen to be the marginal, $q = p_{\mathcal{D}}$, and the learning setup is symmetric.

Let us also confirm that our interchangeable use of the latents $(\mathbf{u}, \mathbf{v})$ and signal variables $(\mathbf{x}, \mathbf{y})$ is formally correct; due to the transformation theorem for any distribution $p_{\mathbf{u}}(\mathbf{u}) = p_{\mathbf{x}}(\mathbf{g}(\mathbf{u})) \det \mathbf{J}_{\mathbf{g}}(\mathbf{u})$ and respectively for $\mathbf{v}, \mathbf{g}', \mathbf{y}$. At the minimizer, we then arrive at

$$\log \frac{p(\mathbf{y}|\mathbf{x})}{q(\mathbf{y}|\mathbf{x})} + C(\mathbf{x}) = \log \frac{p(\mathbf{y}|\mathbf{x}) p_{\mathcal{D}}(\mathbf{x})}{q(\mathbf{y}|\mathbf{x}) p_{\mathcal{D}}(\mathbf{x})} + C(\mathbf{x}) \tag{16}$$

$$= \log \frac{p(\mathbf{v}|\mathbf{u}) p_{\mathcal{D}}(\mathbf{u}) \det \mathbf{J}_{\mathbf{g}}(\mathbf{u}) \det \mathbf{J}_{\mathbf{g}'}(\mathbf{v})}{q(\mathbf{v}|\mathbf{u}) p_{\mathcal{D}}(\mathbf{u}) \det \mathbf{J}_{\mathbf{g}}(\mathbf{u}) \det \mathbf{J}_{\mathbf{g}'}(\mathbf{v})} + C'(\mathbf{u}) \tag{17}$$

$$= \log \frac{p(\mathbf{v}|\mathbf{u})}{q(\mathbf{v}|\mathbf{u})} + C'(\mathbf{u}), \quad \text{with } C(\mathbf{g}(\mathbf{u})) = C'(\mathbf{u}), \tag{18}$$

i.e., the minimizer can be equivalently written in terms of the latents and the signal variables.

Minimizers of the InfoNCE loss become bijective

A property that allows us to weaken some of the conditions given by Hyvärinen et al. [2], Zimmermann et al. [13] and Roeder et al. [9] is the observation that the composition of data generating process and feature encoder becomes bijective for the optimal value of the generalized InfoNCE objective. We introduce the following:

Definition 4 (Diversity condition for bijectivity)**.** *The sampling process composed of distributions p and q is sufficiently diverse if their log-likelihoods satisfy*

$$\operatorname{rank}\left(\left[\frac{\partial^2 \log p(\mathbf{v}|\mathbf{u})}{\partial u_i \partial v_j}\right]_{i\in[d],j\in[d]} - \left[\frac{\partial^2 \log q(\mathbf{v}|\mathbf{u})}{\partial u_i \partial v_j}\right]_{i\in[d],j\in[d]}\right) = d, \tag{19}$$

for all $\mathbf{u}$ in the support of the marginal distribution $p_{\mathcal{D}}$ and $d = d'$.

Def. 4 is a mild condition on the distributions p and q: Intuitively, the condition requires that for all samples $\mathbf{u}$, we can sample positive samples $\mathbf{v}$ that sufficiently vary in all d latent directions, which would be independent from the samples given by the negative distribution q. Suppose q is chosen to be uniform; then the condition is fulfilled for common choices like a Normal distribution with $\log p(\mathbf{v}|\mathbf{u}) = Z(\mathbf{u}) - (\mathbf{u}-\mathbf{v})^\top \mathbf{\Sigma}(\mathbf{u}-\mathbf{v})$ (where $\operatorname{rank}(-\mathbf{\Sigma}) = d$) or a von Mises-Fisher distribution with $\log p(\mathbf{v}|\mathbf{u}) = Z(\mathbf{u}) + \kappa \mathbf{u}^\top \mathbf{v}$ (where $\operatorname{rank} \kappa \mathbf{I} = d$).

We make two additional observations: Firstly, for simple distributions q that do not depend on $\mathbf{u}$, the diversity assumption only affects the positive distribution p as the second term vanishes. Secondly, if p and q are selected to train the network to become invariant to one factor v_i with $p(\mathbf{v}|\mathbf{u}) = p(v_i)p(\mathbf{v}_i|\mathbf{u})$ and $q(\mathbf{v}|\mathbf{u}) = p(v_i)q(\mathbf{v}_i|\mathbf{u})$, the distributions $p(v_i)$ will cancel out in the condition, and reduce the rank by one dimension (which is as intended, as the factor should be discarded during training).

From this diversity condition, we can derive bijectivity of the composition $\mathbf{h} = \mathbf{f} \circ \mathbf{g}$ of the data generating process and feature encoder:

Proposition 2. *Assume that:*

1. *ψ with $\psi(\mathbf{u},\mathbf{v}) = \phi(\mathbf{h}(\mathbf{u}), \mathbf{h}'(\mathbf{v}))$ is a minimizer of the InfoNCE objective (Def. 2) in a learning setup as outlined in Def 1.*
2. *The distributions p and q satisfy the diversity condition for bijectivity (Def. 4).*

Then $\mathbf{h}$ and $\mathbf{h}'$ are bijective on the support of $p_{\mathcal{D}}$.

Proof. By Proposition 1, the minimizer of the InfoNCE loss on the support of $p_{\mathcal{D}}$ is

$$\psi(\mathbf{u},\mathbf{v}) = \log \frac{p(\mathbf{v}|\mathbf{u})}{q(\mathbf{v}|\mathbf{u})} + C(\mathbf{u}) \tag{20}$$

For ψ, we compute the second derivatives and arrange them in matrix form as

$$\left[\frac{\partial^2 \psi(\mathbf{u},\mathbf{v})}{\partial u_i \partial v_j}\right]_{i\in[d],j\in[d]} = \mathbf{J}^\top(\mathbf{u})\mathbf{P}(\mathbf{h}(\mathbf{u}), \mathbf{h}'(\mathbf{v}))\mathbf{J}'(\mathbf{v}) \tag{21}$$

where we used the shorthand $\mathbf{P}(\mathbf{a},\mathbf{b})_{ij} := \partial^2\phi(\mathbf{a},\mathbf{b})/\partial \mathbf{a}_i \mathbf{b}_j$. $\mathbf{J}$ is the Jacobi matrix of $\mathbf{h}$, and $\mathbf{J}'$ is the Jacobi matrix of $\mathbf{h}'$. For the right hand side of the previous equation, we note that

$$\operatorname{rank}(\mathbf{J}^\top(\mathbf{u})\mathbf{P}(\mathbf{h}(\mathbf{u}), \mathbf{h}'(\mathbf{v}))\mathbf{J}'(\mathbf{v})) \le \min\{\operatorname{rank}\mathbf{J}(\mathbf{u}), \operatorname{rank}\mathbf{J}'(\mathbf{v}), \operatorname{rank}\mathbf{P}(\mathbf{h}(\mathbf{u}), \mathbf{h}'(\mathbf{v}))\}, \tag{22}$$

and the rank of the left hand side is given by inserting Eq. 20 into the diversity assumption (2):

$$\operatorname{rank}\left[\frac{\partial^2 \psi(\mathbf{u},\mathbf{v})}{\partial u_i \partial v_j}\right]_{i\in[d],j\in[d]} = \operatorname{rank}\left(\left[\frac{\partial^2(\log p(\mathbf{v}|\mathbf{u}) - \log q(\mathbf{v}|\mathbf{u}) + C(\mathbf{u}))}{\partial u_i \partial v_j}\right]_{i\in[d],j\in[d]}\right) = d.$$

Combining both results gives

$$\operatorname{rank}(\mathbf{J}^\top(\mathbf{u})\mathbf{P}(\mathbf{h}(\mathbf{u}), \mathbf{h}'(\mathbf{v}))\mathbf{J}'(\mathbf{v})) = d \le \min\{\operatorname{rank}\mathbf{J}(\mathbf{u}), \operatorname{rank}\mathbf{J}'(\mathbf{v}), \operatorname{rank}\mathbf{P}(\mathbf{h}(\mathbf{u}), \mathbf{h}'(\mathbf{v}))\}, \tag{23}$$

and implies

$$\operatorname{rank}\mathbf{J}(\mathbf{v}) = \operatorname{rank}\mathbf{J}'(\mathbf{u}) = \operatorname{rank}\mathbf{P}(\mathbf{h}(\mathbf{u}), \mathbf{h}'(\mathbf{v})) = d. \tag{24}$$

Then, both Jacobi matrices have full rank on the support of $p_{\mathcal{D}}$, hence $\mathbf{h}$ and $\mathbf{h}'$ are bijective, concluding the proof. □

Notably, this result is independent of the particular choice of the (potentially learnable) similarity measure ϕ. The similarity measure is implicitly constrained by the requirement that ψ needs to match the log-likelihood ratio of p and q up to a constant.

CEBRA models are consistent

We proceed by showing that CEBRA models are *consistent* under weak assumptions on the data distribution. Consistency entails that the embedding spaces of two different models can be mapped onto each other by some known transformation. In this subsection, we consider the class of *linear* transformations and in the following subsection we will discuss alternative transformations. We denote two independently trained CEBRA models as $\{\mathbf{f}, \mathbf{f}'\}$ and $\{\tilde{\mathbf{f}}, \tilde{\mathbf{f}}'\}$, and make statements about when linear transformations exist such that $\mathbf{f} = \mathbf{L}\tilde{\mathbf{f}}$ and $\mathbf{f}' = \mathbf{M}\tilde{\mathbf{f}}'$ for two full rank matrices $\mathbf{L}$ and $\mathbf{M}$.

We begin by recalling the Canonical Discriminative Form and Diversity Condition in Roeder et al. [9], adapted to our notation:

Definition A (Canonical Discriminative Form, Roeder et al. [9]). *Given a data distribution $p_\mathcal{D}(\mathbf{x}, \mathbf{y}, S)$ with random variables $\mathbf{x}$ and $\mathbf{y}$ and a set S containing the possible values of $\mathbf{y}$ given $\mathbf{x}$,*

$$p_\mathcal{D}(\mathbf{y}|\mathbf{x}, S) > 0 \iff \mathbf{y} \in S, \tag{25}$$

a generalized discriminative model family may be defined by its parameterization of the probability of the target variable $\mathbf{y}$ conditioned on an observed variable $\mathbf{x}$ and the set S that contains not only the true target label $\mathbf{y}$, but also a collection of distractors $\mathbf{y}'$:

$$p_{\mathbf{f},\mathbf{f}'}(\mathbf{y}|\mathbf{x}, S) = \frac{\exp(\mathbf{f}(\mathbf{x})^\top \mathbf{f}'(\mathbf{y}))}{\sum_{\mathbf{y}' \in S} \exp(\mathbf{f}(\mathbf{x})^\top \mathbf{f}'(\mathbf{y}'))}. \tag{26}$$

Note that the feature extractors $\mathbf{f}$ and $\mathbf{f}'$ could be two separate networks, as we already discussed in Suppl. Note 1. Roeder et al. [9] consider $\mathbf{f}$ to be a "data encoder" and $\mathbf{f}'$ to be a "context encoder". This is in contrast to the setup by Hyvärinen et al. [2] which we will revisit in the context of recovering the data generating factors, where $\mathbf{f}(\mathbf{x})$ would play the role of an auxiliary variable while $\mathbf{f}'$ is the feature encoder later used in downstream tasks and analysis.

In CEBRA, the choice and role of both functions can be configured, which is why we will discuss theoretical guarantees for both use cases. We proceed by re-stating two diversity conditions needed for $\mathbf{f}$ or $\mathbf{f}'$ to become consistent:

Definition B (Diversity conditions for consistency, Roeder et al. [9]). *Let $Z(\mathbf{x}, S) := -\log \sum_{\mathbf{y}' \in S} \exp(\mathbf{f}(\mathbf{x})^\top \mathbf{f}'(\mathbf{y}'))$. Assume that for the encoders $(\mathbf{f}, \mathbf{f}', \tilde{\mathbf{f}}, \tilde{\mathbf{f}}')$ for which it holds that $p_{\mathbf{f},\mathbf{f}'} = p_{\tilde{\mathbf{f}},\tilde{\mathbf{f}}'}$*

1. *for any given $\mathbf{y}$, there exist $M+1$ tuples $\{(\mathbf{x}^{(i)}, S^{(i)})\}_{i=1}^{M+1}$, such that $p_\mathcal{D}(\mathbf{x}^{(i)}, \mathbf{y}, S^{(i)}) > 0$, and such that the $((M+1) \times (M+1))$ matrices $\mathbf{M}$ and $\tilde{\mathbf{M}}$ are invertible, where $\mathbf{M}$ consists of columns $[-Z(\mathbf{x}^{(i)}, S^{(i)}); \mathbf{f}(\mathbf{x}^{(i)})]$, and $\tilde{\mathbf{M}}$ consists of columns $[-Z(\mathbf{x}^{(i)}, S^{(i)}); \tilde{\mathbf{f}}(\mathbf{x}^{(i)})]$,*
2. *for any given $\mathbf{x}$, by repeated sampling $S \sim p_D(S|\mathbf{x})$ and picking two points $\mathbf{y}_A, \mathbf{y}_B \in S$, we can construct a set of M distinct tuples $(\mathbf{y}_A^{(i)}, \mathbf{y}_B^{(i)})_{i=1}^{M}$ such that the matrices $\mathbf{L}$ and $\tilde{\mathbf{L}}$ are invertible, where $\mathbf{L}$ consists of columns $(\mathbf{f}'(\mathbf{y}_A(i)) - \mathbf{f}'(\mathbf{y}_B(i)))$, and $\tilde{\mathbf{L}}$ consists of columns $(\tilde{\mathbf{f}}'(\mathbf{y}_A(i)) - \tilde{\mathbf{f}}'(\mathbf{y}_B(i)))$, $i \in 1, \ldots, M$.*

With the diversity condition in place, we recall Theorem 1 from Roeder et al. [9], adapted to our notation:

Theorem A (Roeder et al. [9]). *Under the diversity condition (Def. B), models following the canonical discriminative form (Def. A) are linearly identifiable. That is, for any encoders $\{\mathbf{f}, \mathbf{f}'\}$, and $\{\tilde{\mathbf{f}}, \tilde{\mathbf{f}}'\}$ it holds that*

$$p_{\mathbf{f},\mathbf{f}'} = p_{\tilde{\mathbf{f}},\tilde{\mathbf{f}}'} \quad \implies \quad \mathbf{f}(\mathbf{x}) = \mathbf{L}\tilde{\mathbf{f}}(\mathbf{x}), \quad \mathbf{f}'(\mathbf{y}) = \mathbf{L}\tilde{\mathbf{f}}'(\mathbf{y}) \tag{27}$$

for all samples ($\mathbf{x}$, $\mathbf{y}$) in the support of the data distribution.

Proof. See Theorem 1, Roeder et al. [9], where we replaced the equivalence condition on the right hand side by inserting its definition for clarity. □

We can leverage this result as CEBRA falls into the class of models models described in Definition A:

Proposition 3 (CEBRA models are consistent.). *Assume that two CEBRA models are trained on data from the same latent data distribution, and denote the feature encoders of the trained models as $\mathbf{f}, \mathbf{f}'$ and $\tilde{\mathbf{f}}, \tilde{\mathbf{f}}'$. Further assume that for both models, the similarity measure ϕ is the dot-product similarity and ψ minimizes the generalized InfoNCE loss. Finally assume that the diversity condition (Def. B) holds. Then the feature encoders $\mathbf{f}$, $\mathbf{f}'$ are consistent up to a linear transformation, and $\mathbf{f}(\mathbf{x}) = \mathbf{L}\tilde{\mathbf{f}}(\mathbf{x})$, $\mathbf{f}'(\mathbf{y}) = \mathbf{L}'\tilde{\mathbf{f}}'(\mathbf{y})$, for linear transformations $\mathbf{L}$, $\mathbf{L}'$ for any pair of points $\mathbf{x}, \mathbf{y}$ in the support of the data distribution.*

Proof. The generalized InfoNCE objective with limited samples (Def. 3) can be written as

$$\mathop{\mathbb{E}}_{\substack{\mathbf{x}\sim p(\mathbf{x}),\ \mathbf{y}_+\sim p(\mathbf{y}|\mathbf{x}) \\ \mathbf{y}_1,\dots,\mathbf{y}_n\sim q(\mathbf{y}|\mathbf{x})}}[\log p(\mathbf{y}^+|\mathbf{x},S)], \quad \log p(\mathbf{y}^+|\mathbf{x},S) = -\log\frac{\exp\psi(\mathbf{x},\mathbf{y}_+)}{\sum_{i=1}^n \exp\psi(\mathbf{x},\mathbf{y}_i)} = -\log\frac{\exp(\mathbf{f}(\mathbf{x})^\top\mathbf{f}'(\mathbf{y}_+)/\tau)}{\sum_{i=1}^n \exp(\mathbf{f}(\mathbf{x})^\top\mathbf{f}'(\mathbf{y}_i)/\tau)} \tag{28}$$

which matches the canonical discriminative form (Def. A) with encoders $\mathbf{f}(\cdot)$ and $\mathbf{f}'(\cdot)/\tau$. The composition of the set $S := \{\mathbf{y}_i\}_{i=1}^N$ is given by the distribution $p_{\mathcal{D}}(\mathbf{x})q(\mathbf{y}|\mathbf{x})$ and S fulfills Def. B by assumption. At the minimizer, the values of the loss functions match, from which it follows that $p_{\mathbf{f},\mathbf{f}'} = p_{\tilde{\mathbf{f}},\tilde{\mathbf{f}}'}$. Hence, we apply Theorem A to find that $\mathbf{f}$ and $\mathbf{f}'$ are consistent up to a linear transform, concluding the proof. □

It is worth noting that this result also *holds for datasets with limited samples*, i.e., the objective in Def. 3, as long as the dataset fulfills the diversity condition (Def. B). Checking the diversity condition as well as matching distributions for the two CEBRA models is possible in practice using only the dataset and the trained model.

Both diversity conditions from Roeder et al. [9] depend on the variability of the ground truth latent distribution (and the presence of this variability after mapping the latents to signal space), and on the properties of the encoders $\mathbf{f}$ and $\mathbf{f}'$. While Roeder et al. [9] already discuss that in practice, a randomized neural network will fulfill the diversity conditions, with our diversity criterion for bijectivity (Def. 4) and the bijectivity of $\mathbf{f}$ and $\mathbf{f}'$ that follows, we can strengthen this argument and ensure that both conditions hold upon convergence for minimizers of the generalized InfoNCE objective:

Proposition 4. *Assume that the encoders* $(\mathbf{f}, \mathbf{f}', \tilde{\mathbf{f}}, \tilde{\mathbf{f}}')$ *and hence also the compositions of data generator and encoders* $(\mathbf{h}, \mathbf{h}', \tilde{\mathbf{h}}, \tilde{\mathbf{h}}')$ *minimize the InfoNCE objective. Assume that upon convergence, the diversity condition for bijectivity (Def. 4) holds. Then,* $\mathbf{h}(\mathbf{u}) = \mathbf{A}\mathbf{h}(\mathbf{u})$ *and* $\mathbf{h}'(\mathbf{v}) = \mathbf{B}\tilde{\mathbf{h}}'(\mathbf{v})$ *for all latents* $\mathbf{u}, \mathbf{v}$ *in the support of the data distribution for two full-rank matrices* $\mathbf{A}$, $\mathbf{B}$.

Proof. Both models share the minimizer

$$\mathbf{h}(\mathbf{u})^\top\mathbf{h}'(\mathbf{v}) = \tilde{\mathbf{h}}(\mathbf{u})^\top\tilde{\mathbf{h}}'(\mathbf{v}) = \log\frac{p(\mathbf{v}|\mathbf{u})}{q(\mathbf{v}|\mathbf{u})} + C(\mathbf{u}) \tag{29}$$

and we have

$$\mathbf{h}(\mathbf{u})^\top\mathbf{h}'(\mathbf{v}) = \tilde{\mathbf{h}}(\mathbf{u})^\top\tilde{\mathbf{h}}'(\mathbf{v}) \tag{30}$$

taking derivatives with respect to $\mathbf{v}$, and then to $\mathbf{u}$, we arrive at

$$\mathbf{J}(\mathbf{u})^\top\mathbf{J}'(\mathbf{v}) = \tilde{\mathbf{J}}(\mathbf{u})^\top\tilde{\mathbf{J}}'(\mathbf{v}) \tag{31}$$

where all Jacobian matrices have full rank due to Prop. 2. We can hence derive

$$\mathbf{J}'(\mathbf{v}) = (\mathbf{J}(\mathbf{u})^{-\top}\tilde{\mathbf{J}}(\mathbf{u})^\top)\tilde{\mathbf{J}}'(\mathbf{v}) \quad \mathbf{J}(\mathbf{u})^\top = \tilde{\mathbf{J}}(\mathbf{u})^\top(\tilde{\mathbf{J}}'(\mathbf{v})\mathbf{J}'(\mathbf{v})^{-1}) \tag{32}$$

$$\mathbf{J}'(\mathbf{v}) = \mathbf{A}(\mathbf{u})\tilde{\mathbf{J}}'(\mathbf{v}) \quad \mathbf{J}(\mathbf{u})^\top = \tilde{\mathbf{J}}(\mathbf{u})^\top\mathbf{B}(\mathbf{v}) \tag{33}$$

for some full rank matrices $\mathbf{A}(\mathbf{u})$ and $\mathbf{B}(\mathbf{v})$. Because the left hand side do not depend on the argument of $\mathbf{A}$ and $\mathbf{B}$, both matrices need to be constant, leaving

$$\mathbf{J}'(\mathbf{v}) = \mathbf{A}\tilde{\mathbf{J}}'(\mathbf{v}) \quad \mathbf{J}(\mathbf{u})^\top = \tilde{\mathbf{J}}(\mathbf{u})^\top\mathbf{B} \tag{34}$$

from which it follows that

$$\mathbf{h}(\mathbf{u}) = \mathbf{B}^{-1}\tilde{\mathbf{h}}(\mathbf{u}) \quad \mathbf{h}'(\mathbf{v}) = \mathbf{A}\tilde{\mathbf{h}}'(\mathbf{v}). \tag{35}$$

concluding the proof. □

Note that the previous proposition applies even when the data generating functions (i.e., the datasets) between the model run differ, and merely the latent distributions match. In this case, the same latent $\mathbf{u}_0$ can be mapped to different points $\mathbf{x}_0$ and $\tilde{\mathbf{x}}_0$ and the resulting embedding points would still satisfy $\mathbf{f}(\mathbf{u}_0) = \mathbf{A}\tilde{\mathbf{f}}(\mathbf{u}_0)$. For this reason, the proposition is written w.r.t. the composition $\tilde{\mathbf{h}}$ of data generating process and encoder. If the data generating processes match, it is clear that Eq. 35 can be equivalently written as $\mathbf{f}(\mathbf{x}) = \mathbf{B}^{-1}\tilde{\mathbf{f}}(\mathbf{x})$, $\mathbf{f}'(\mathbf{y}) = \mathbf{A}\tilde{\mathbf{f}}'(\mathbf{y})$, which matches the statement by Roeder et al. [9] (but for our modified diversity condition).

For symmetric encoders, we can give the following result:

Proposition 5. *Assume that the encoders $\mathbf{f} = \mathbf{f}', \tilde{\mathbf{f}} = \tilde{\mathbf{f}}'$ are shared, and $-\phi$ is a norm, $\phi(\mathbf{a}, \mathbf{b}) = -\|\mathbf{a} - \mathbf{b}\|$. Assume that the model minimizes the InfoNCE loss and assume that the co-domain of $\mathbf{f}, \mathbf{f}'$ is a normed space over $\mathbb{R}^d$. Then $\mathbf{h} = \mathbf{L}\tilde{\mathbf{h}}$.*

Proof. We write the data in terms of the underlying latents $\mathbf{x} = \mathbf{g}(\mathbf{u})$ and $\mathbf{y} = \mathbf{g}'(\mathbf{v})$. At the minimizer of the InfoNCE loss it then holds that

$$\|\mathbf{h}(\mathbf{u}) - \mathbf{h}(\mathbf{v})\| + C(\mathbf{u}) = \|\tilde{\mathbf{h}}(\mathbf{u}) - \tilde{\mathbf{h}}(\mathbf{v})\| + \tilde{C}(\mathbf{u}). \tag{36}$$

for all points in the dataset. Inserting $\mathbf{v} = \mathbf{u}$ gives $C(\mathbf{u}) = \tilde{C}(\mathbf{u})$. Because $\mathbf{h}, \tilde{\mathbf{h}}$ bijective, we define points $\mathbf{a} = \tilde{\mathbf{h}}(\mathbf{u})$ and $\mathbf{b} = \tilde{\mathbf{h}}(\mathbf{v})$, and it holds

$$\|\mathbf{h}(\tilde{\mathbf{h}}^{-1}(\mathbf{a})) - \mathbf{h}(\tilde{\mathbf{h}}^{-1}(\mathbf{b}))\| = \|\mathbf{a} - \mathbf{b}\|. \tag{37}$$

Due to the Mazur–Ulam theorem, the map $\mathbf{h} \circ \tilde{\mathbf{h}}^{-1}$ is then affine, concluding the proof. □

Note that all results in this section are also independent of the mixing functions $\mathbf{g}$ and $\mathbf{g}'$. This means that *consistency can be guaranteed irrespective of the exact data modality and generative process*, as long as the *underlying* latent distribution matches. This is given in well-controlled experiments (as one assumes when e.g., repeating experiments).

CEBRA models recover the ground-truth latents

In contrast to the identifiability results in the previous section (which made a connection between two models trained on the same or similar data distribution), in this section we will make a connection between the *ground truth model* and the model trained on the data. To make this connection, assumptions are needed about the ground truth model.

As introduced in Def. 1, we will denote the ground truth model(s) as $\mathbf{g}$ and $\mathbf{g}'$, and data from these models is then encoded with $\mathbf{f}$ and $\mathbf{f}'$, respectively. Our goal is to understand properties of their composition $\mathbf{h} = \mathbf{g} \circ \mathbf{f}$ and $\mathbf{h}' = \mathbf{g}' \circ \mathbf{f}'$, and when this composition reduces to an affine or linear transformation. Based on the property of the model (especially the similarity measure), other guarantees are possible. Towards the end of this subsection we will discuss CEBRA model setups where we obtain guarantees up to permutations and sign-flips and point-wise non-linear transformations by leveraging existing contrastive learning theory [2, 13]. We base our theory on the identifiability proofs of contrastive learning given by Hyvärinen et al. [2] and Zimmermann et al. [13], and give new proofs to complete the theory for the most important usage modes in CEBRA. Note that the theory also extends to settings not explicitly demonstrated in this paper, but integrated into the CEBRA software package (e.g., trainable similarity functions ϕ).

One key property of CEBRA is its distinction of discovery-driven and hypothesis-driven training (or hybrid training, which is a combination of both where the feature encoders need to minimize both the time-contrastive and behavior-contrastive objectives). For time- and behavior-contrastive learning, similar theory applies. A key difference is that in time-contrastive (discovery-driven) learning, an underlying distribution of the latents is inherent to the dataset and "given" by the temporal variation in the data. If it is desirable to recover the ground truth latents, the similarity measure needs to be suitable to allow full InfoNCE minimization, e.g., by model selection on basis of the InfoNCE or goodness of fit metric. Note that a cosine similarity measure is already quite flexible for a wide range of distributions and a good default, and also note that InfoNCE minimization is known to be empirically robust to minor violations between the model assumptions and ground-truth conditional distribution [13]. For hypothesis-driven learning, CEBRA will always be able to find a suitable learning setup that recovers the auxiliary variables: The positive and negative sample distributions can be chosen and selected such that the propositions in this section will hold. Even if empirical distributions are used (e.g., the "time delta" distribution outlined in the Methods), it is possible to check that this distribution matches the similarity measure of the model.

Because we make statements about the relationship between continual and discrete context variables ($\mathbf{c}_t$ and k_t) and the signal space ($\mathbf{s}_t$) for each time-point t, we will again use this notation from the Methods; we use the variable names introduced in Def. 1 to denote the underlying ground-truth latents, and the samples fed to the model (which can, but do not necessarily need to, equal to the signal). We start our discussion with discovery-driven training of CEBRA using time information:

Proposition 6 (Discovery-driven CEBRA)**.** *Assume the learning setup in Def. 1, and that the ground-truth latents $\mathbf{u}_1, \ldots, \mathbf{u}_T$ for each time point follow a uniform marginal distribution and the change between subsequent time steps is given by the conditional distribution of the form*

$$p(\mathbf{u}_{t+\Delta t}|\mathbf{u}_t) = \frac{1}{Z(\mathbf{u}_t)} \exp \delta(\mathbf{u}_{t+\Delta t}, \mathbf{u}_t) \tag{38}$$

where δ is either a (scaled) dot product (and $\mathbf{u}_t \in \mathcal{S}^{n-1} \subset \mathbb{R}^d$ lies on the $(n-1)$-sphere $\mathcal{S}^{n-1}$) or an arbitrary semi-metric (and $\mathbf{u}_t \in \mathcal{U} \subset \mathbb{R}^d$ lies in a convex body $\mathcal{U}$). Assume that the data generating process $\mathbf{g}$ with $\mathbf{s}_t = \mathbf{g}(\mathbf{u}_t)$ is injective. Assume we train a

symmetric CEBRA model with encoder $\mathbf{f} = \mathbf{f}'$ *and the similarity measure including a fixed temperature* $\tau > 0$ *is set to or sufficiently flexible such that* $\phi = \delta$ *for all arguments. Then* $\mathbf{h} = \mathbf{h}' = \mathbf{g} \circ \mathbf{f}$ *is affine.*

Proof. For δ being the dot product, the result follows from the proof of Theorem 2 in Zimmermann et al. [13]. For δ being a semi-metric, the result follows from the proof of Theorem 5 in Zimmermann et al. [13]. □

We will next consider the hypothesis-driven mode in CEBRA. Here, we either choose a parametric or non-parametric positive distribution p to shape the embedding space. Our goal is find an embedding space reflecting the auxiliary variable, in case there is a meaningful relationship between the signal and this variable.

Naturally, the actual signal will depend on additional latents that we do not record as auxiliary information. The full data generating process can be written as

$$\mathbf{s}_t = \mathbf{g}(\mathbf{c}_t, k_t, \mathbf{z}_t) \tag{39}$$

where $\mathbf{z}_t$ are additional latent sources not observed during training. Since $\mathbf{g}$ is an injective function, it follows that $\mathbf{s}_t = \mathbf{s}_{t'}$ implies $\mathbf{c}_t = \mathbf{c}_{t'}$, $k_t = k_{t'}$, $\mathbf{z}_t = \mathbf{z}_{t'}$. Applying hypothesis-driven learning in this setup will force an embedding space representing $\mathbf{c}$ and k, but not $\mathbf{z}$. We can denote the set $G(\mathbf{c}, k) := \{\mathbf{z} \in Z : \mathbf{g}(\mathbf{c}, k, \mathbf{z})\}$ which contains all points in signal space corresponding to a particular set of auxiliary variables $\mathbf{c}, k$. Because $\mathbf{g}$ is injective, there exists a function $\tilde{\mathbf{g}}$, which will retrieve the original auxiliary variables: $\tilde{\mathbf{g}}(\mathbf{g}(\mathbf{c}, k, \mathbf{z})) = (\mathbf{c}, k)^\top$.

Proposition 7 (Hypothesis-driven CEBRA)**.** *Assume a partially observable data generating process, where*

$$\mathbf{y} = \mathbf{g}'(\mathbf{c}, \mathbf{v}) \tag{40}$$

with $\mathbf{g}'$ *injective and with* $\mathbf{c}$ *as an observable context variable, and* $\mathbf{v}$ *as a latent. As in Prop. 6 assume that* $\mathbf{c}$ *lies on a hypersphere if* ϕ *is the dot-product similarity, and on a convex body if* $-\phi$ *is a semi-metric. Then the minimizer of the InfoNCE loss trained with a distribution* $p(\mathbf{c}|\tilde{\mathbf{c}}) = \exp(\phi(\mathbf{c}, \tilde{\mathbf{c}}))/Z(\tilde{\mathbf{c}})$ *and a uniform marginal and negative distribution* $q = p_{\mathcal{D}}$ *will yield a* $\mathbf{h}$ *that recovers* $\mathbf{c}$ *up to an affine transformation.*

Proof. $\mathbf{h}$ is the composition $\mathbf{g} \circ \mathbf{f}$. Per assumption, the similarity function ϕ is sufficiently flexible such that

$$\phi(\mathbf{h}(\mathbf{c}, \mathbf{z}), \mathbf{h}(\tilde{\mathbf{c}}, \mathbf{z})) = \log p(\mathbf{c}, \tilde{\mathbf{c}}) + C(\tilde{\mathbf{c}}) \quad \forall \mathbf{z}. \tag{41}$$

Because $\mathbf{g}$ is injective, $\mathbf{g}(\mathbf{c}, \mathbf{z}) = \mathbf{g}(\mathbf{c}', \mathbf{z}')$ implies that $\mathbf{c} = \mathbf{c}'$ and $\mathbf{z} = \mathbf{z}'$ and there exists a function $\tilde{\mathbf{g}}$ such that $\tilde{\mathbf{g}}(\mathbf{g}(\mathbf{c}, \mathbf{z})) = \mathbf{c}$ for all $\mathbf{z}$. It remains to show that $\mathbf{f} = \mathbf{L}\tilde{\mathbf{g}}$ at the minimizer for a full-rank matrix $\mathbf{L}$. By comparing arguments on the left hand side and right and side, and inserting the form of p, solutions of Eq. 41 need to simplify to

$$\phi(\mathbf{h}(\mathbf{c}), \mathbf{h}(\tilde{\mathbf{c}})) = \phi(\mathbf{c}, \tilde{\mathbf{c}}) + C(\tilde{\mathbf{c}}) \tag{42}$$

By symmetry of ϕ, $C(\tilde{\mathbf{c}}) = 0$ vanishes. Then the result follows again from Theorem 2 in Zimmermann et al. [13] for ϕ being the dot-product similarity, and from Theorem 5 in Zimmermann et al. [13] for $-\phi$ being a semi-metric. □

With our results from Prop. 1 and Prop. 2, it is also possible for us to extend the results for distributions fulfilling the diversity condition for bijectivity (Def. 4). The following proposition enables a statement about distribution where the data generating process and/or feature encoder differs between the reference and positive/negative pairs. An example used in the main text is multi-session training, or training across data modalities. We first need to strengthen the assumption about the positive and negative distributions from Def. 4. Again assume that the latent dimensions $d = d'$ match.

Definition 5 (Strong assumption of diversity for bijectivity)**.** *The sampling process with distributions* p *and* q *satisfies*

$$\left[\frac{\partial^2 \log p(\mathbf{v}|\mathbf{u})}{\partial u_i \partial v_j}\right]_{i\in[d], j\in[d]} - \left[\frac{\partial^2 \log q(\mathbf{v}|\mathbf{u})}{\partial u_i \partial v_j}\right]_{i\in[d], j\in[d]} = \mathbf{L} \tag{43}$$

for some full-rank matrix $\mathbf{L} \in \mathbb{R}^{d\times d}$ *for all* $\mathbf{u} \in \mathbb{R}^d$ *in the support of the marginal distribution* $p_{\mathcal{D}}$*.*

Note that Def. 5 is a special case of the diversity condition for bijectivity in Def. 4. The stronger condition still covers important distributions like a Gaussian conditional, even with an additional mean and covariance $p(\mathbf{v}|\mathbf{u}) = \exp(-(\mathbf{u} - \mathbf{v} - \mu)^\top \mathbf{\Sigma}^{-1}(\mathbf{u} - \mathbf{v} - \mu))$. Likewise, it would cover van Mises-Fisher (vMF) distributions, even with a rotated reference sample and $p(\mathbf{v}|\mathbf{u}) = \exp(\kappa \mathbf{u}^\top \mathbf{Q}\mathbf{v})$. We will cover these selected special cases using the stronger diversity assumption below.

Proposition 8. *Consider a data generating process with uniform marginal and positive/negative distributions satisfying Def. 5. Assume the data is generated by mixing functions* $\mathbf{g} : \mathbb{R}^d \mapsto \mathbb{R}^D$ *and* $\mathbf{g}' \in \mathbb{R}^d \mapsto \mathbb{R}^{D'}$ *and encoded into a shared E-dimensional embedding space with two separate encoders* $\mathbf{f} : \mathbb{R}^D \mapsto \mathbb{R}^E$ *and* $\mathbf{f}' : \mathbb{R}^{D'} \mapsto \mathbb{R}^E$ *and assume that* ϕ *is the dot-product. Then there are* d *dimensions in* $\mathbf{h}$*, and* d *dimensions in* $\mathbf{h}'$ *which represent the latents up to an affine transform.*

Proof. Let us again denote the compositions $\mathbf{h} = \mathbf{g} \circ \mathbf{f} : \mathbb{R}^d \mapsto \mathbb{R}^E$ and $\mathbf{h}' = \mathbf{g}' \circ \mathbf{f}' : \mathbb{R}^d \mapsto \mathbb{R}^E$ and let us denote the Jacobian matrices as $\mathbf{J} : \mathbb{R}^d \mapsto \mathbb{R}^{E\times d}$ and $\mathbf{J}' : \mathbb{R}^d \mapsto \mathbb{R}^{E\times d}$, respectively. Without loss of generality (the indices can be arbitrarily permuted), let us split each $\mathbf{h}$ into two parts, with $\mathbf{h}_1 := [h_1, \ldots, h_d]^\top$ and $\mathbf{h}_2 := [h_{d+1}, \ldots, h_E]^\top$ (in case $E > d$), and respectively for $\mathbf{h}'$. At the minimizer of the InfoNCE loss, we get the condition

$$\mathbf{h}(\mathbf{u})^\top \mathbf{h}'(\mathbf{v})/\tau = \log \frac{p(\mathbf{v}|\mathbf{u})}{q(\mathbf{v}|\mathbf{u})} + C(\mathbf{u}). \tag{44}$$

We take the derivative w.r.t. $\mathbf{v}$ on both sides, with gives

$$\mathbf{J}'(\mathbf{v})^\top \mathbf{h}(\mathbf{u})/\tau = \frac{\partial}{\partial \mathbf{v}} \left[\log \frac{p(\mathbf{v}|\mathbf{u})}{q(\mathbf{v}|\mathbf{u})} \right], \tag{45}$$

with $\mathbf{J}'$ denoting the Jacobian matrix of $\mathbf{h}'$. We now take the derivative w.r.t. $\mathbf{u}$ on both sides, with gives

$$\mathbf{J}(\mathbf{u})^\top \mathbf{J}'(\mathbf{v})/\tau = \frac{\partial^2}{\partial \mathbf{u} \partial \mathbf{v}} \left[\log \frac{p(\mathbf{v}|\mathbf{u})}{q(\mathbf{v}|\mathbf{u})} \right]. \tag{46}$$

and by assumption we can insert Def. 5 and re-arrange to

$$\mathbf{J}(\mathbf{u})\mathbf{J}'(\mathbf{v})^\top = \tau \mathbf{L} \tag{47}$$

for some full-rank matrix $\mathbf{L} \in \mathbb{R}^{d\times d}$. Note that when $E > d$ this condition is underconstrained. Without loss of generality, let us split the Jacobian matrices into two parts:

$$\mathbf{J}(\mathbf{u}) := [\mathbf{J}_1(\mathbf{u}); \mathbf{0}] \quad \mathbf{J}'(\mathbf{v}) := [\mathbf{J}_1'(\mathbf{v}); \mathbf{J}_2'(\mathbf{v})] \tag{48}$$

where $\mathbf{J}_1 : \mathbb{R}^d \mapsto \mathbb{R}^{d\times d}$, $\mathbf{J}_1' : \mathbb{R}^d \mapsto \mathbb{R}^{d\times d}$, $\mathbf{J}_2' : \mathbb{R}^d \mapsto \mathbb{R}^{(E-d)\times d}$. This allows to re-write the condition as

$$\mathbf{J}_1(\mathbf{u})\mathbf{J}_1'(\mathbf{v})^\top = \tau \mathbf{L} \tag{49}$$

which is no longer underconstrained. This equation is valid for any $\mathbf{u}, \mathbf{v}$ in the support of the marginal and the positive/negative distribution, which is why the left hand side cannot depend on $\mathbf{u}$ and $\mathbf{v}$, leaving the final form of the Jacobians:

$$\mathbf{J}(\mathbf{u}) := [\mathbf{J}_1; \mathbf{0}] \quad \mathbf{J}'(\mathbf{v}) := [\mathbf{J}_1'; \mathbf{a}(\mathbf{v})], \tag{50}$$

where $\mathbf{a} : \mathbb{R}^d \mapsto \mathbb{R}^{(E-d)\times d}$ is an arbitrary function that ensures that Eq. 44 can hold. It follows that $\mathbf{h}_1$ and $\mathbf{h}_1'$ are affine transforms, $\mathbf{h}_2$ is a constant, and $\mathbf{h}_2'$ will be a potentially non-linear transform to match the InfoNCE minimizer, concluding the proof. □

We next discuss two special cases that are of interest to users of CEBRA and demonstrate that the previous result is more flexible than the results presented in Zimmermann et al. [13]. Consider a setup where we have a constant "offset" between the latents vs. having a perfectly symmetric p. We are still able to recover the underlying latents for both vMF and Normal conditional distributions:

Corollary 1. *The aforementioned result holds for vMF distributions with a constant offset between* $\mathbf{u}$ *and* $\mathbf{v}$ *on the hypersphere, parameterized by a rotation matrix* $\mathbf{Q}$*, with* $\log p(\mathbf{v}|\mathbf{u}) = \kappa \mathbf{u}^\top \mathbf{Q} \mathbf{v}$*. Assume* $d = d' = k$ *and assume the domain and co-domain of* $\mathbf{h}$, $\mathbf{h}'$ *is the unit sphere.*

Proof. The distributions satisfies constancy of the second derivative, and the condition on the Jacobian matrices is

$$\mathbf{J}_1 \mathbf{J}_1'^\top = (\tau\kappa)\mathbf{Q} \tag{51}$$

and since all vectors $\mathbf{h}(\mathbf{a}) = \mathbf{J}^\top \mathbf{a}$ need to be normalized and $\mathbf{Q}$ is orthogonal, we can consider any column $\mathbf{q}_i$ and the corresponding vector $\mathbf{a}_i$ of $\mathbf{J}_1$

$$\mathbf{J}_1 \mathbf{a}_i = (\tau\kappa)\mathbf{q}_i \Rightarrow \|\mathbf{J}_1 \mathbf{a}_i\|^2 = (\tau\kappa)\|\mathbf{q}_i\|^2 \Rightarrow 1 = (\tau\kappa)1 \tag{52}$$

and it follows $\tau = 1/\kappa$. □

Corollary 2. *The aforementioned result holds for a Gaussian distribution with a constant offset between* $\mathbf{u}$ *and* $\mathbf{v}$ *parameterized, with* $\log p(\mathbf{v}|\mathbf{u}) = \|\mathbf{u} - \mathbf{v} - \mu\|^2$. *Assume* $d = d' = k$.

Proof. We rewrite the log-conditional as

$$\log p(\mathbf{v}|\mathbf{u}) = -\|(\mathbf{u} - \mathbf{v}) - \boldsymbol{\mu}\|^2 \tag{53}$$
$$= -\|\mathbf{u} - \mathbf{v}\|^2 - \|\boldsymbol{\mu}\|^2 + 2(\mathbf{u} - \mathbf{v})^\top \boldsymbol{\mu} \tag{54}$$
$$= -\|\mathbf{u}\|^2 - \|\mathbf{v}\|^2 - \|\boldsymbol{\mu}\|^2 + 2\mathbf{u}^\top \boldsymbol{\mu} + 2\mathbf{v}^\top \boldsymbol{\mu} + 2\mathbf{u}^\top \mathbf{v} \tag{55}$$
$$\tag{56}$$

which gives, at the minimizer of the InfoNCE objective,

$$\mathbf{h}(\mathbf{u})^\top \mathbf{h}'(\mathbf{v})/\tau = 2\mathbf{u}^\top \mathbf{v} + (-\|\mathbf{v}\|^2 + 2\mathbf{v}^\top \boldsymbol{\mu}) + (-\|\mathbf{u}\|^2 + 2\mathbf{u}^\top \boldsymbol{\mu} + C(\mathbf{u})) \tag{57}$$

and we recover

$$\mathbf{h}_1(\mathbf{u}) = \mathbf{J}_1 \mathbf{u}, \tag{58}$$
$$\mathbf{h}_1'(\mathbf{v}) = \mathbf{J}_1' \mathbf{v}, \tag{59}$$
$$C(\mathbf{u}) = \|\mathbf{u}\|^2 - 2\mathbf{u}^\top \boldsymbol{\mu} \tag{60}$$
$$\mathbf{h}_2'(\mathbf{v}) = -\|\mathbf{v}\|^2 + 2\mathbf{v}^\top \boldsymbol{\mu}. \tag{61}$$

□

While the above results extended the theory of Zimmermann et al. [13] to training setups within the CEBRA library and used in the main text (training with dot-product and negative mean squared error as the similarity measure and sampling procedure), we can also leverage previous results from Hyvärinen et al. [2] to further extend the families of possible distributions.

Firstly, for conditional exponential family distributions within an ICA framework, the dot-product similarity can be used in conjunction with non-symmetric encoders $\mathbf{f}$, $\mathbf{f}'$ (i.e., two separate networks) to recover the components up to a linear indeterminacy. We recall Definition 1 from Hyvärinen et al. [2]:

Definition C (Conditionally exponential distributions (Def. 1 from Hyvärinen et al. [2]))**.** *A random variable (independent component)* v_i *is conditionally exponential of order* k *given random vector* $\mathbf{x}$ *if its conditional probability density function can be given in the form*

$$p(v_i|\mathbf{x}) = \frac{Q_i(v_i)}{Z_i(\mathbf{x})} \exp\left[\sum_{j=1}^{k} \tilde{q}_{ij}(v_i)\lambda_{ij}(\mathbf{x})\right] \tag{62}$$

almost everywhere in the support of $\mathbf{x}$, *with* $\tilde{q}_{ij}$, λ_{ij}, Q_i, *and* Z_i *scalar-valued functions. The sufficient statistics* $\tilde{q}_{ij}$ *are assumed linearly independent (over* j, *for each fixed* i*).*

We observe a similar functional form as used in Prop. 8. Values of $\tilde{q}_{ij}(v_i)$ would be represented by $\mathbf{f}'$, and values in $\lambda_{ij}(\mathbf{x})$ would be represented by $\mathbf{f}$. As in the previous proposition, more dimensions in $\mathbf{f}$, $\mathbf{f}'$ as latent variables are required ($E > d$) for representing conditional distributions of the conditional exponential form. In this setup, Theorem 3 from Hyvärinen et al. [2] implies in our context that the sufficient statistics of the latents can be recovered up to a linear transformation.

Secondly, for arbitrary conditional distributions, as long as variability conditions are satisfied within an ICA framework, contrastive learning can recover the underlying components up to a permutation and point-wise invertible transformation [2]. Using this mode applies when a similarity measure ϕ which gives

$$\psi(\mathbf{u}, \mathbf{v}) := \sum_{i=1}^{E} \phi_i(h_i'(\mathbf{v}), \mathbf{h}(\mathbf{u})), \text{ or w.r.t. to the signal variables, } \psi(\mathbf{x}, \mathbf{y}) = \sum_{i=1}^{E} \phi_i(f_i'(\mathbf{y}), \mathbf{f}(\mathbf{x})), \tag{63}$$

is used within the CEBRA framework. In this case, Theorem 1 in Hyvärinen et al. [2] applies and gives an identifiability guarantee of $\mathbf{h}$ to recover the ground truth latents $\mathbf{v}$ up to permutations and component-wise non-linear transformations.

Supplementary Tables

Supplementary Table 1: Consistency statistics related to Fig. 1. Data includes all rats (n=4, with 10 seeds per model run averaged first); one-way ANOVA F(24.5) $p = 1.92x10^{-16}$.

group 1	group 2	P-value	Reject
CEBRA-Behavior	CEBRA-Time	0.0108	**True**
CEBRA-Behavior	conv-pi-VAE w/labels	0.0021	**True**
CEBRA-Behavior	conv-pi-VAE without	0.0	**True**
CEBRA-Behavior	tSNE	0.0	**True**
CEBRA-Behavior	UMAP	0.0	**True**
CEBRA-Behavior	autoLFADS	0.0	**True**
CEBRA-Time	conv-pi-VAE w/labels	0.9988	False
CEBRA-Time	conv-pi-VAE without	0.0001	**True**
CEBRA-Time	tSNE	.4808	False
CEBRA-Time	UMAP	0.0	**True**
CEBRA-Time	autoLFADS	0.1129	False

Supplementary Table 2: Decoding Statistics related to Fig. 2. Data includes all rats (n=4); supervised grouping one way ANOVA F(55) p=4.7e-31; self- and unsupervised, one way ANOVA F(14.7) $p = 1.53x10^{-10}$. Posthoc Tukey HSD Tests:

group 1	group 2	P-value	Reject
CEBRA-Behavior	conv-pi-VAE (MC decoding)	0.9	False
CEBRA-Behavior	conv-pi-VAE (kNN)	0.001	**True**
CEBRA-Behavior	pi-VAE (MC decoding)	0.001	**True**
CEBRA-Behavior	pi-VAE (kNN)	0.001	**True**
CEBRA-Time	autoLFADS	0.0	**True**
CEBRA-Time	PCA	0.0	**True**
CEBRA-Time	tSNE	0.0174	**True**
CEBRA-Time	UMAP	0.0368	**True**

Supplementary Table 3: Related to Fig. 5. One-way ANOVA F(3, 197)=5.88, $p = 0.0007$ and posthoc Tukey HSD tests. Allen Neuropixels dataset, 1 Frame window (below 50 neurons all False):

neuron no.	group 1	group 2	P-value	Reject
50	baseline-bayes	baseline-knn	0.0233	**True**
50	baseline-bayes	CEBRA	0.8918	False
50	baseline-knn	CEBRA	0.0055	True
50	baseline-bayes	CEBRA-joint	0.8238	False
50	baseline-knn	CEBRA-joint	0.004	**True**
50	CEBRA	CEBRA-joint	0.9987	False
100	baseline-bayes	baseline-knn	0.0	**True**
100	baseline-bayes	CEBRA	0.0275	**True**
100	baseline-knn	CEBRA	0.0132	**True**
100	baseline-bayes	CEBRA-joint	0.9977	False
100	baseline-knn	CEBRA-joint	0.0	**True**
100	CEBRA	CEBRA-joint	0.0395	**True**
200	baseline-bayes	baseline-knn	0.0005	**True**
200	baseline-bayes	CEBRA	0.3703	False
200	baseline-knn	CEBRA	0.0	**True**
200	baseline-bayes	CEBRA-joint	0.0058	**True**
200	baseline-knn	CEBRA-joint	0.0	**True**
200	CEBRA	CEBRA-joint	0.1478	False
400	baseline-bayes	baseline-knn	0.0044	**True**
400	baseline-bayes	CEBRA	0.0336	**True**
400	baseline-knn	CEBRA	0.0	**True**
400	baseline-bayes	CEBRA-joint	0.0	**True**
400	baseline-knn	CEBRA-joint	0.0	**True**
400	CEBRA	CEBRA-joint	0.0	**True**
600	baseline-bayes	baseline-knn	0.0125	**True**
600	baseline-bayes	CEBRA	0.6063	False
600	baseline-knn	CEBRA	0.001	**True**
600	baseline-bayes	CEBRA-joint	0.0	**True**
600	baseline-knn	CEBRA-joint	0.0	**True**
600	CEBRA	CEBRA-joint	0.0	**True**
800	baseline-bayes	baseline-knn	0.0006	**True**
800	baseline-bayes	CEBRA	0.0008	**True**
800	baseline-knn	CEBRA	0.0	**True**
800	baseline-bayes	CEBRA-joint	0.0	**True**
800	baseline-knn	CEBRA-joint	0.0	**True**
800	CEBRA	CEBRA-joint	0	**True**
900	baseline-bayes	baseline-knn	0.0004	**True**
900	baseline-bayes	CEBRA	0.0048	**True**
900	baseline-knn	CEBRA	0.0	**True**
900	baseline-bayes	CEBRA-joint	0.0	**True**
900	baseline-knn	CEBRA-joint	0.0	**True**
900	CEBRA	CEBRA-joint	0.0	**True**
1000	baseline-bayes	baseline-knn	0.0	**True**
1000	baseline-bayes	CEBRA	0.0	**True**
1000	baseline-knn	CEBRA	0.0	**True**
1000	baseline-bayes	CEBRA-joint	0.0	**True**
1000	baseline-knn	CEBRA-joint	0.0	**True**
1000	CEBRA	CEBRA-joint	0.0019	**True**

Supplementary Table 4: Related to Fig. 5. One-way ANOVA $F(3, 197)= 1.29$, $p= 0.279$ and posthoc Tukey HSD tests. Allen Neuropixels dataset, 10 Frame window (< 30 neurons= all were False). Note, here CEBRA vs. CEBRA-joint is not significant.

neuron no.	group 1	group 2	P-value	Reject
30	baseline-bayes	baseline-knn	0.016	**True**
30	baseline-bayes	CEBRA	0.1255	False
30	baseline-knn	CEBRA	0.7083	False
30	baseline-bayes	CEBRA-joint	0.0072	**True**
30	baseline-knn	CEBRA-joint	0.9784	False
30	CEBRA	CEBRA-joint	0.4762	False
50	baseline-bayes	baseline-knn	0.0358	**True**
50	baseline-bayes	CEBRA	0.324	False
50	baseline-knn	CEBRA	0.5956	False
50	baseline-bayes	CEBRA-joint	0.1296	False
50	baseline-knn	CEBRA-joint	0.8989	False
50	CEBRA	CEBRA-joint	0.9379	False
100	baseline-bayes	baseline-knn	0.0	**True**
100	baseline-bayes	CEBRA	0.2589	False
100	baseline-knn	CEBRA	0.0002	**True**
100	baseline-bayes	CEBRA-joint	0.372	False
100	baseline-knn	CEBRA-joint	0.0001	**True**
100	CEBRA	CEBRA-joint	0.9941	False
200	baseline-bayes	baseline-knn	0.0	**True**
200	baseline-bayes	CEBRA	0.9976	False
200	baseline-knn	CEBRA	0.0	**True**
200	baseline-bayes	CEBRA-joint	0.7999	False
200	baseline-knn	CEBRA-joint	0.0	**True**
200	CEBRA	CEBRA-joint	0.6964	False
400	baseline-bayes	baseline-knn	0.0004	**True**
400	baseline-bayes	CEBRA	0.2531	False
400	baseline-knn	CEBRA	0.0	**True**
400	baseline-bayes	CEBRA-joint	0.2166	False
400	baseline-knn	CEBRA-joint	0.0	**True**
400	CEBRA	CEBRA-joint	0.9996	False
600	baseline-bayes	baseline-knn	0.0002	**True**
600	baseline-bayes	CEBRA	0.2095	False
600	baseline-knn	CEBRA	0.0	**True**
600	baseline-bayes	CEBRA-joint	0.2884	False
600	baseline-knn	CEBRA-joint	0.0	**True**
600	CEBRA	CEBRA-joint	0.9967	False
800	baseline-bayes	baseline-knn	0.0001	**True**
800	baseline-bayes	CEBRA	0.3691	False
800	baseline-knn	CEBRA	0.0	**True**
800	baseline-bayes	CEBRA-joint	0.1668	False
800	baseline-knn	CEBRA-joint	0.0	**True**
800	CEBRA	CEBRA-joint	0.9524	False
900	baseline-bayes	baseline-knn	0.0003	**True**
900	baseline-bayes	CEBRA	0.3867	False
900	baseline-knn	CEBRA	0.0	**True**
900	baseline-bayes	CEBRA-joint	0.3522	False
900	baseline-knn	CEBRA-joint	0.0	**True**
900	CEBRA	CEBRA-joint	0.9999	False
1000	baseline-bayes	baseline-knn	0.0018	**True**
1000	baseline-bayes	CEBRA	0.4707	False
1000	baseline-knn	CEBRA	0.0001	**True**
1000	baseline-bayes	CEBRA-joint	0.3785	False
1000	baseline-knn	CEBRA-joint	0.0001	**True**
1000	CEBRA	CEBRA-joint	0.9981	False

Supplementary Table 5: Related to Fig. 5. One-way ANOVA F(3, 197) = 15.73, $p = 3.31x10^{-9}$ and posthoc Tukey HSD test. Allen Neuropixels dataset, scene classification with 1 Frame window:

neuron no.	group 1	group 2	P-value	Reject
50	baseline-bayes	baseline-knn	0.4575	False
50	baseline-bayes	CEBRA	0.1986	False
50	baseline-knn	CEBRA	0.9355	False
50	baseline-bayes	CEBRA-joint	0.0	**True**
50	baseline-knn	CEBRA-joint	0.0	**True**
50	CEBRA	CEBRA-joint	0.0	**True**
100	baseline-bayes	baseline-knn	0.0207	**True**
100	baseline-bayes	CEBRA	0.0084	**True**
100	baseline-knn	CEBRA	0.9694	False
100	baseline-bayes	CEBRA-joint	0.0	**True**
100	baseline-knn	CEBRA-joint	0.0	**True**
100	CEBRA	CEBRA-joint	0.0	**True**
200	baseline-bayes	baseline-knn	0.0106	**True**
200	baseline-bayes	CEBRA	0.0001	**True**
200	baseline-knn	CEBRA	0.1269	False
200	baseline-bayes	CEBRA-joint	0.0	**True**
200	baseline-knn	CEBRA-joint	0.0	**True**
200	CEBRA	CEBRA-joint	0.0	**True**
400	baseline-bayes	baseline-knn	0.0047	**True**
400	baseline-bayes	CEBRA	0.0001	**True**
400	baseline-knn	CEBRA	0.239	False
400	baseline-bayes	CEBRA-joint	0.0	**True**
400	baseline-knn	CEBRA-joint	0.0	**True**
400	CEBRA	CEBRA-joint	0.0	**True**
600	baseline-bayes	baseline-knn	0.0013	**True**
600	baseline-bayes	CEBRA	0.0	**True**
600	baseline-knn	CEBRA	0.0032	**True**
600	baseline-bayes	CEBRA-joint	0.0	**True**
600	baseline-knn	CEBRA-joint	0.0	**True**
600	CEBRA	CEBRA-joint	0.0	**True**
800	baseline-bayes	baseline-knn	0.0	**True**
800	baseline-bayes	CEBRA	0.0	**True**
800	baseline-knn	CEBRA	0.0	**True**
800	baseline-bayes	CEBRA-joint	0.0	**True**
800	baseline-knn	CEBRA-joint	0.0	**True**
800	CEBRA	CEBRA-joint	0.0	**True**
900	baseline-bayes	baseline-knn	0.0062	**True**
900	baseline-bayes	CEBRA	0.0	**True**
900	baseline-knn	CEBRA	0.0168	**True**
900	baseline-bayes	CEBRA-joint	0.0	**True**
900	baseline-knn	CEBRA-joint	0.0	**True**
900	CEBRA	CEBRA-joint	0.0	**True**
1000	baseline-bayes	baseline-knn	0.0002	**True**
1000	baseline-bayes	CEBRA	0.0	**True**
1000	baseline-knn	CEBRA	0.0	**True**
1000	baseline-bayes	CEBRA-joint	0.0	**True**
1000	baseline-knn	CEBRA-joint	0.0	**True**
1000	CEBRA	CEBRA-joint	0.0	**True**

Supplementary Table 6: Related to Fig. 5. One-way ANOVA (10 frame window, 1000 neurons) F(3, 16) = 20.22, p = $1.09x10^{-5}$ and posthoc Tukey HSD tests. Allen Neuropixels dataset, Mean frame error, 10 frames

neuron no.	group 1	group 2	P-value	Reject
1000	baseline-bayes	baseline-knn	0.5277	False
1000	baseline-bayes	CEBRA	0.0013	**True**
1000	baseline-knn	CEBRA	0.0001	**True**
1000	baseline-bayes	CEBRA-joint	0.0011	**True**
1000	baseline-knn	CEBRA-joint	0.0001	**True**
1000	CEBRA	CEBRA-joint	0.9996	False

References

[1] Y. Gao, E. Archer, L. Paninski, and J. P. Cunningham. Linear dynamical neural population models through nonlinear embeddings. In *NIPS*, 2016.

[2] A. Hyvärinen, H. Sasaki, and R. E. Turner. Nonlinear ICA using auxiliary variables and generalized contrastive learning. In *The 22nd International Conference on Artificial Intelligence and Statistics*, volume 89 of *Proceedings of Machine Learning Research*, pages 859–868. PMLR, 2019. URL http://proceedings.mlr.press/v89/hyvarinen19a.html.

[3] M. R. Keshtkaran, A. R. Sedler, R. H. Chowdhury, R. Tandon, D. Basrai, S. L. Nguyen, H. Sohn, M. Jazayeri, L. E. Miller, and C. Pandarinath. A large-scale neural network training framework for generalized estimation of single-trial population dynamics. *Nature Methods*, 2022.

[4] D. Kobak, W. Brendel, C. Constantinidis, C. E. Feierstein, A. Kepecs, Z. F. Mainen, X.-L. Qi, R. Romo, N. Uchida, and C. K. Machens. Demixed principal component analysis of neural population data. *eLife*, 5, 2016.

[5] L. McInnes, J. Healy, and J. Melville. Umap: Uniform manifold approximation and projection for dimension reduction. *arXiv preprint arXiv:1802.03426*, 2018.

[6] A. v. d. Oord, Y. Li, and O. Vinyals. Representation learning with contrastive predictive coding. *arXiv preprint arXiv:1807.03748*, 2018.

[7] C. Pandarinath, D. J. O'Shea, J. Collins, R. Józefowicz, S. D. Stavisky, J. C. Kao, E. M. Trautmann, M. T. Kaufman, S. I. Ryu, L. R. Hochberg, J. M. Henderson, K. V. Shenoy, L. F. Abbott, and D. Sussillo. Inferring single-trial neural population dynamics using sequential auto-encoders. *Nature methods*, 15:805 – 815, 2018.

[8] F. Pei, J. Ye, D. Zoltowski, A. Wu, R. H. Chowdhury, H. Sohn, J. E. O'Doherty, K. V. Shenoy, M. T. Kaufman, M. Churchland, et al. Neural latents benchmark'21: Evaluating latent variable models of neural population activity. *arXiv preprint arXiv:2109.04463*, 2021.

[9] G. Roeder, L. Metz, and D. P. Kingma. On linear identifiability of learned representations. *arXiv*, 2020. doi: 10.48550/ARXIV.2007.00810. URL https://arxiv.org/abs/2007.00810.

[10] A. E. Urai, B. Doiron, A. M. Leifer, and A. K. Churchland. Large-scale neural recordings call for new insights to link brain and behavior. *Nature Neuroscience*, 25:11–19, 2022.

[11] T. Wang and P. Isola. Understanding contrastive representation learning through alignment and uniformity on the hypersphere. In *International Conference on Machine Learning*, pages 9929–9939. PMLR, 2020.

[12] D. Zhou and X. Wei. Learning identifiable and interpretable latent models of high-dimensional neural activity using pi-vae. In *Advances in Neural Information Processing Systems 33*, 2020. URL https://proceedings.neurips.cc/paper/2020/hash/510f2318f324cf07fce24c3a4b89c771-Abstract.html.

[13] R. S. Zimmermann, Y. Sharma, S. Schneider, M. Bethge, and W. Brendel. Contrastive learning inverts the data generating process. In *Proceedings of the 38th International Conference on Machine Learning*, volume 139 of *Proceedings of Machine Learning Research*, pages 12979–12990. PMLR, 2021. URL http://proceedings.mlr.press/v139/zimmermann21a.html.
